# The Progress of Tobacco Control Research in Sub-Saharan Africa in the Past 50 Years: A Systematic Review of the Design and Methods of the Studies

**DOI:** 10.3390/ijerph15122732

**Published:** 2018-12-04

**Authors:** Hadii M. Mamudu, Pooja Subedi, Ali E. Alamin, Sreenivas P. Veeranki, Daniel Owusu, Amy Poole, Lazarous Mbulo, A.E. Ogwell Ouma, Adekunle Oke

**Affiliations:** 1 Department of Health Services Management and Policy, College of Public Health, East Tennessee State University, Johnson City, TN 37614, USA; elgasimalial@goldmail.etsu.edu (A.E.A.); ampoole74@gmail.com (A.P.); okeao1@etsu.edu (A.O.); 2 Department of Biostatistics and Epidemiology, East Tennessee State University, Johnson City, TN 37614, USA; subedip@goldmail.etsu.edu; 3 Department of Preventive Medicine and Community Health, University of Texas Medical Branch, Galveston, TX 77555, USA; drveeranki@gmail.com; 4 Tobacco Center of Regulatory Science (GSU TCORS), Georgia State University, Atlanta, GA 30340, USA; dowusu2@gsu.edu; 5 Global Tobacco Control Branch, Office on Smoking and Health, Centers for Disease Control and Prevention, Atlanta, GA 30329, USA; vyp7@cdc.gov; 6 Tobacco Control Division, WHO Regional Office for Africa, P.O.Box 06 Brazzaville, Congo; oumae@who.int

**Keywords:** Sub-Sahara Africa, tobacco control research, methodological design, peer-reviewed publications, research desert, research capacity

## Abstract

Over one billion of the world’s population are smokers, with increasing tobacco use in low- and middle-income countries. However, information about the methodology of studies on tobacco control is limited. We conducted a literature search to examine and evaluate the methodological designs of published tobacco research in Sub-Saharan Africa (SSA) over the past 50 years. The first phase was completed in 2015 using PubMed, Embase, CINAHL, and Cochrane Central Register of Controlled Trials. An additional search was completed in February 2017 using PubMed. Only tobacco/smoking research in SSA countries with human subjects and published in English was selected. Out of 1796 articles, 447 met the inclusion criteria and were from 26 countries, 11 of which had one study each. Over half of the publications were from South Africa and Nigeria. The earliest publication was in 1968 and the highest number of publications was in 2014 (*n* = 46). The majority of publications used quantitative methods (91.28%) and were cross-sectional (80.98%). The commonest data collection methods were self-administered questionnaires (38.53%), interviews (32.57%), and observation (20.41%). Around half of the studies were among adults and in urban settings. We conclud that SSA remains a “research desert” and needs more investment in tobacco control research and training.

## 1. Introduction

What does this paper add?

- Although tobacco control research involving Sub-Saharan Africa (SSA) continues to grow, the region is still a “research desert”.
- There is the need for research capacity building for Sub-Saharan Africa (SSA).
- Cohort studies and randomized control trials involving populations in Sub-Saharan Africa (SSA) are lacking and need to be pursued.

Despite scientific evidence on the health dangers of tobacco use and secondhand tobacco smoke (SHS) exposure [1,2,3,4,5,6], over one billion of the world’s population continue to smoke, with increasing usage in low- and middle-income countries (LMICs) where over 80% of the world’s smokers currently reside [7,8,9,10]. For Sub-Saharan Africa (SSA), it has been estimated that tobacco use will increase up to 37% [8] by 2025 [8], the fastest expected growth among the six regions of the World Health Organization (WHO). The shift of the activities of transnational tobacco companies (TTCs) to LMICs [11,12,13,14,15], alongside general socioeconomic transformation [15,16,17,18] and lag in tobacco control [15,19] has made tobacco use a major public health concern in SSA. A key challenge to the efforts to address this public health issue in SSA has been sparse research, resulting from inadequate tobacco control research capacity [20,21,22]. Moreover, there is limited information and knowledge about how the existing studies on tobacco control were conducted; hence, the need for a review of studies in the region to inform tobacco regulatory science, policy and program development, and advocacy initiatives.

The existing tobacco control research in SSA has ranged from assessment of the prevalence of tobacco use (smoked, smokeless products, and recently, electronic nicotine delivery systems—ENDS) among youths and adults [23,24,25,26,27], impacts of tobacco use [28,29,30], tobacco industry activities to undermine tobacco control policies [31,32], to tobacco control policy development [33,34]. Except for a few countries [23,35], the prevalence studies have reported low [36,37] but increasing tobacco use, and the gender-gap between males and females is consistent with the tobacco epidemic model [38,39]. Additionally, there is increasing research in SSA about the tobacco industry activities not only to promote tobacco use but also to undermine policy. This area of research has involved how the TTCs have undermined policy through lobbying politicians [32,40,41,42,43], promoted tobacco use [9,34,42,44,45], threatened governments with litigations [34,43,44], used economic benefit arguments to promote tobacco [32,46], corporate social responsibility (CSR) programs [44,47], and engaged in smuggling to make tobacco available and easily accessible to both youth and adults [40,48]. Further, studies on the impacts of tobacco use have ranged from the prevalence of tobacco-induced diseases, such as cancer and cardiovascular diseases (CVD) [29,49,50,51], to the negative environmental consequences of tobacco farming [52]. In terms of policy studies, the research has involved tobacco policy development with a primary focus on policies of institutions, opinions, and attitudes toward institutional policies such as those of schools [53]. and development and assessment of the impact of specific policies such as excise tax. In this respect, much of the policy studies have been conducted in South Africa [54,55,56]. The development of the 2003 WHO Framework Convention on Tobacco Control (WHO FCTC), the first international public health treaty, negotiated under the auspices of WHO with almost all countries in SSA as signatories, has created the critical need for research to inform the WHO FCTC adoption and implementation in the region [31,33,43,57]. Thus, given that only a few systematic reviews about tobacco control research in SSA has been conducted [58,59], and none on the nature and scope of existing research, we conducted a systematic review of the designs and methods for tobacco control research utilized by published peer-reviewed studies involving countries in SSA up to February 2017 to inform tobacco regulatory science.

Several designs and methodological approaches to research exist [60,61,62], ranging from randomized controlled trials (RCT) to prospective cohort, cross-sectional studies, and case-control studies. The research methodology has impact on the scientific quality and the interpretation of the results, which is critical for tobacco control research where there is an industry with the goal to obfuscate the science for tobacco control [63,64,65]. Therefore, the objective of this study was to examine research design and methods used by tobacco control studies involving SSA. in order to inform improvement in the quality of such research in the region. Research suggests that scientific evidence is essential for tobacco control [66,67,68]. The evidence from this study will highlight the scientific progress in tobacco control research in SSA over the past 50 years. Countries in SSA are mostly in Stage I of the Tobacco Epidemic Model [38,39], which suggests that research in the region is critically needed to inform preventive and control initiatives to stop or reverse the progression to higher stages of the tobacco epidemic model. In particular, the implementation of the WHO FCTC requires strong research base [67]; hence, the importance of this systematic review of tobacco control research in SSA.

## 2. Methods

### 2.1. Literature Search and Selection

In June and July 2015, two librarians in the James H. Quillen College of Medicine at East Tennessee State University comprehensively searched for tobacco control studies conducted in SSA from four databases—PubMed (via Medline), Embase (via Scopus), CINAHL (via EBSCO), Cochrane Central Register of Control Trials (via EBSCO)—using search terms provided by the authors, including Boolean search term “Africa OR Sub-Saharan Africa AND Tobacco OR Smoke OR Nicotine AND Research AND Studies”. A total of 2209 publications were retrieved and uploaded in Mendeley.com, an online reference management system. Subsequently, in February 2017, one research assistant conducted a follow-up comprehensive search from PubMed (via Medline) using the same search terms. Duplicate articles identified using Mendeley database were discarded and 1796 were included for further consideration. These publications were further screened by two research assistants, starting with titles and abstracts of the articles, and subsequently followed by screening the full text of the articles for inclusion based on predetermined inclusion criteria. Articles were included if they were original tobacco control investigations conducted in any of the countries in the Sub-Saharan Africa (SSA) region and written in English with full text available. The two research assistants and the lead author met to resolve any disagreements during the eligibility screening. The bibliographies of selected articles were inspected and relevant references were retrieved for further screening. Additional articles (*n* = 123) were identified from bibliography of the selected articles. (The full lists of the eligible peer-reviewed publications are in Supplementary Table S1).

### 2.2. Data Extraction

**Figure 1:**
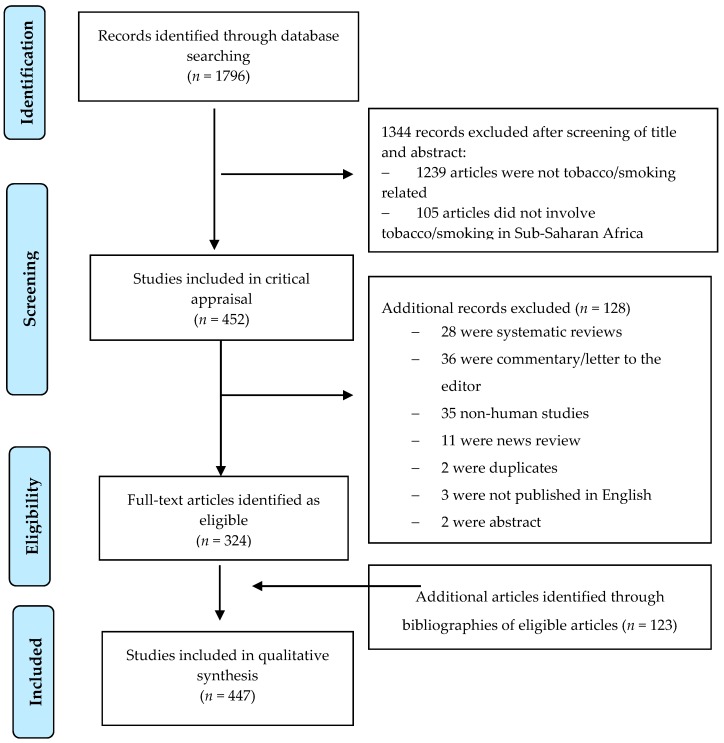
Flow Diagram.

The inclusion criteria set by the authors for the selection of studies were as follows: (1) Only research articles; (2) research on tobacco/smoking in a WHO African region country; (3) non-tobacco industry-sponsored studies or trials; and (4) completed studies. The exclusion criteria were as follows: (1) not tobacco-related; (2) health outcome studies where tobacco was only one of the risk factors considered; and (3) substance abuse studies where tobacco was not the main focus of the study. Two research assistants extracted data from the 447 studies that met the eligibility criteria, independently (Table S1). A predefined form was used for data extraction. Data extracted included author, study site, year of publication, area of research, sample size, sample characteristics, type of sampling, mean age of sample, sex distribution, place of recruitment, location, study design, type of study design, data characteristics, power, eligibility criteria, and use of Global Youth Tobacco Survey (GYTS). Discrepancies in data extracted by the two research assistants were resolved through discussion with the lead author to reach a consensus. Consistent with PRISMA [69], the workflow is presented in Figure 1.

### 2.3. Quality Appraisal

We utilized Grading of Recommendations Assessment, Development, and Evaluation (GRADE: http://www.gradeworkinggroup.org/#pub) Guidelines, which assess the quality of a research based on the type of study design (randomized trials vs. observational studies), the risk of bias, the consistency of the results across studies, and the precision of the overall estimate across studies, for this study. Specifically, we focused on the portion of GRADE about the type of study design, which evaluates the quality of evidence for randomized trial as “high” or “moderate” and for observational studies as “low” or “very low,” with variations across these types of design.

### 2.4. Data Analysis

Descriptive statistics were used to analyze the information extracted from the publications on different aspects of their methods and designs like date of publication, country, type of study, and characteristics of the sample. Frequencies, percentage, and graphs were used to report the findings. Preliminary descriptive analyses were completed during the early stage of data analysis to check if there was inconsistency in data extraction. Where any unexpected value was obtained, the associated research article was reviewed again, and corrections were made.

## 3. Results

### 3.1. The Tobacco Control Environment in SSA

The SSA region of the world consists of 47 countries in the African continent, including large and highly populated ones such as Nigeria and South Africa, and small countries such as Equatorial Guinea, Gambia, Mauritius, and Togo. Although there are differences among these countries in terms of economic development, political systems, religion, and language, almost all of them have been classified as LMICs by the World Bank and have similar cultural orientations and a common political history. Further, despite the differences in native languages, English and French are the national languages of majority of the countries in SSA. In this respect, these countries share several significant similarities to be considered together in a research study.

In terms of tobacco control, the countries in SSA have varied experiences and different levels of advancement, with countries such as South Africa having highly-advanced tobacco control programs and others such as Malawi having very limited progress [70,71,72,73,74]. As of December 2017, only seven out of the 47 countries in the region have comprehensive tobacco control programs [75,76] although all have ratified the WHO FCTC. Moreover, WHO reports [75,76,77,78,79] and other studies [15,80], suggest that countries in SSA generally lag behind those in other regions in terms of progress in tobacco control [77,78,79,81,82]. Although the smoking epidemic is yet to gain a strong foothold in the region [38,39,83], all the countries have experienced the shift in the TTCs’ activities toward them [11,12,13,14,15]. The tobacco industry continues to exert influence in the region although only a few countries, including Democratic Republic of Congo (DRC), Kenya, South Africa, and Tanzania have domestic tobacco companies, with the British American Tobacco (BAT) as the dominant TTC in many countries [71,72,73]. Further, although tobacco farming occurs across countries in SSA, tobacco production is significant economic activity in few countries, particularly those in East Africa and only a handful of countries, especially Malawi and Zimbabwe, are dependent on tobacco production [84]. These similarities in tobacco control environment in SSA suggests that results of research studies can be emulated and easily diffused to reduce the increasing trends of tobacco use [8].

### 3.2. Tobacco Research in Sub-Saharan Africa

**Figure 2:**
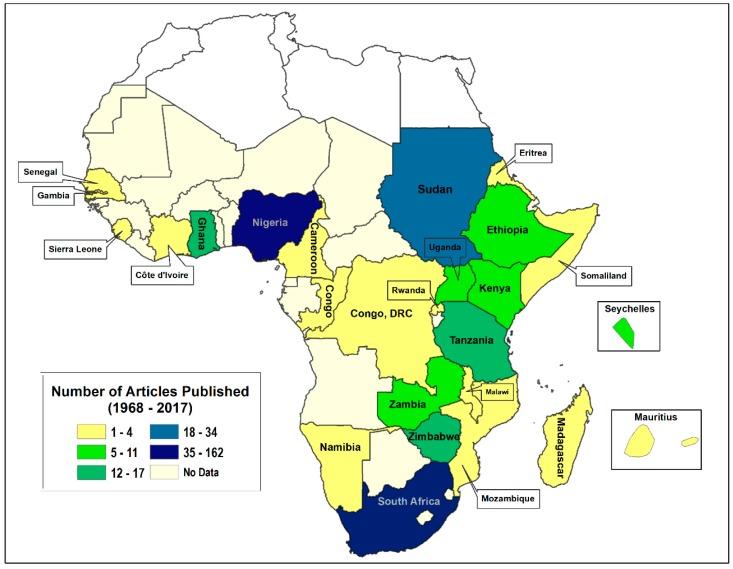
Distribution of peer-reviewed tobacco control publications in Sub-Saharan Africa (up to February 2017; *n* = 447).

The 447 articles reviewed for this study were from 26 countries in SSA (Table S1 shown online; Figure 2), with 36.24% (162) and 21.48% (96) conducted in South Africa and Nigeria, respectively. Additionally, around 9% (43) of the studies included multiple countries or regional data. Of the 26 countries with tobacco control studies, 42.31% (11) had only one peer-reviewed scientific publication. This suggests that, except for South Africa and Nigeria, there was no sustained tobacco control research in many countries in SSA at the time this review was conducted. The distribution of the 447 studies is shown in Figure 2.

### 3.3. Yearly Publications

**Figure 3:**
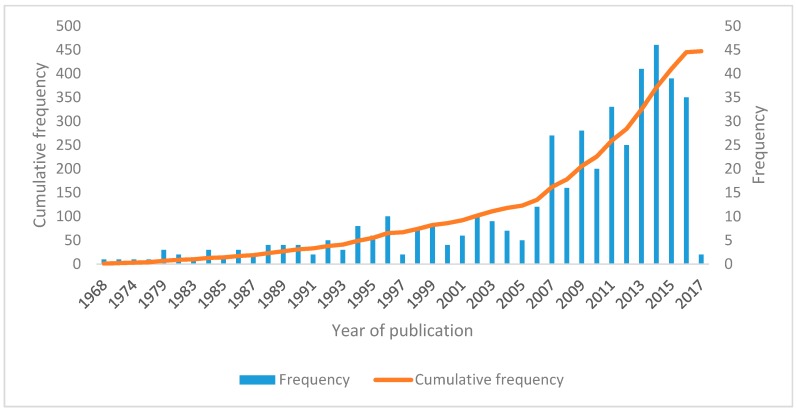
Yearly publication of peer-reviewed tobacco control studies in Sub-Saharan Africa (SSA) (up to February 2017; *n* = 447).

The earliest peer-reviewed journal publication related to tobacco in SSA identified in this study was by Gelfand et al. in 1968 [85] (Figure 3). This study assessed the association between carcinoma of bronchus and the smoking habit among Rhodesian (now Zimbabwean) Africans.

From 1968 to 1991 only one to four articles were published every year, except 1969, 1971–1973, 1975, 1977, 1978, 1980, and 1981 when no article on tobacco was published from the SSA region. The number of peer-reviewed publications was highly sporadic and slow until the early 2000s when it started to increase. In particular, after WHO FCTC was adopted, the number of publications increased dramatically. Indeed, between 2006 and 2016 the cumulative number of publications over the 11-year period was 322. Overall, the largest number of articles (46) was published in 2014. While only 111 articles were published from the year 1968 to 2003 (36 years), a greater number of articles was published (336) from 2004 to 2017 (14 years).

### 3.4. Type of Tobacco Control Research (Scientific Inquiry)

The broad types of scientific inquiries are quantitative, qualitative, and mixed methods [61,62]. Of the 447 peer-reviewed articles, 91.28% (*n* = 408) were quantitative studies, while 7.83% (*n* = 35) were qualitative studies, and the remaining 0.89% (*n* = 4) used mixed methods (Figure 2). Thus, scientific inquiry pertaining to tobacco control research in SSA has been mainly based on quantitative method or approach.

### 3.5. Type of Research Study Designs

**Figure 4:**
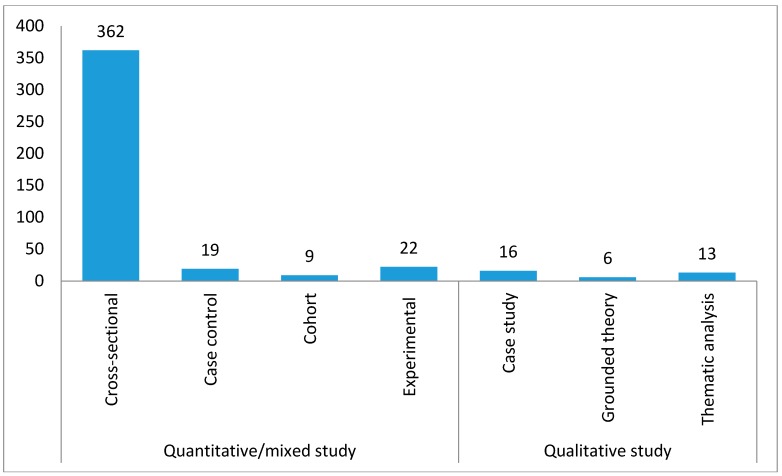
Types of Research Study Design in SSA (up to February 2017; *n* = 447).

Several research designs exist within the broad categories of scientific inquiry in SSA. Overall, 4.9% (*n* = 22) were experimental studies or randomized trials and 95% (*n* = 425) were observational studies. The majority of the studies were cross-sectional in nature (*n* = 362, 80.98%). Around 4% (*n* = 19) used case control studies, 3.58% were case studies (*n* = 16), and 2% of the studies were cohort studies (*n* = 9). Remaining studies used grounded theories (*n* = 6, 1.34%) and thematic analysis (*n* = 13, 2.91%, Figure 4).

### 3.6. Research Data Characteristics

#### 3.6.1. Nature of Data Collection from Study Participants

**Figure 5:**
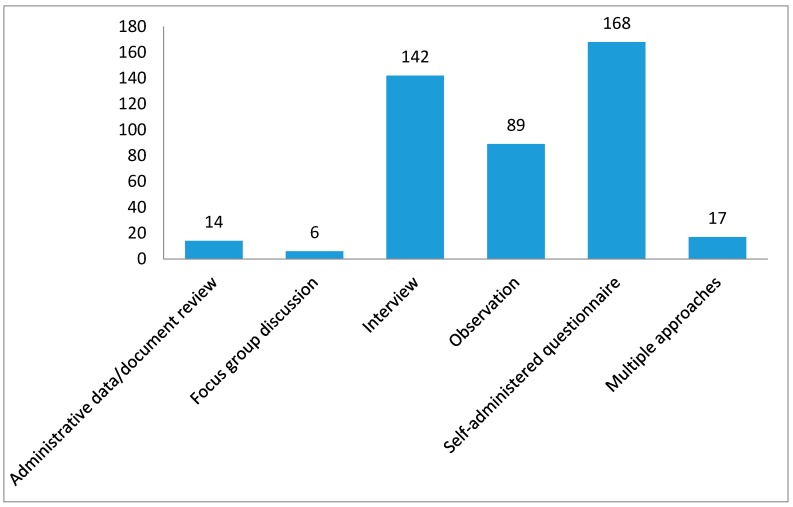
Type of Data used for Tobacco Control Research in SSA (up to February 2017; *n* = 436). Note: All the surveys that involved interviewers asking the survey questions to the respondents were classified as interviews, all the surveys that measured some variables (e.g., anthropometry, blood pressure) or observed any behaviors were classified as observation, even though they also interviewed or used self-administered questionnaires to collect further information.

The rigor of a scientific study depends on the way data are acquired or collected from the study participants. We were able to retrieve information on the underlying data characteristics for 436 peer-reviewed publications in SSA. Of these 436 publications, the majority of the studies (*n* = 168, 38.53%; Figure 5) were generated through self-administered questionnaires, followed by interviews (*n* = 142, 32.57%). Observation methods were also commonly used to collect information (*n* = 89, 20.41%). Other studies used data from administrative records/documents (*n* = 14, 3.21%) and focus group discussions (*n* = 6, 1.38%). Seventeen studies (390%) used multiple approaches to generate data for their studies.

#### 3.6.2. Study Setting

**Figure 6:**
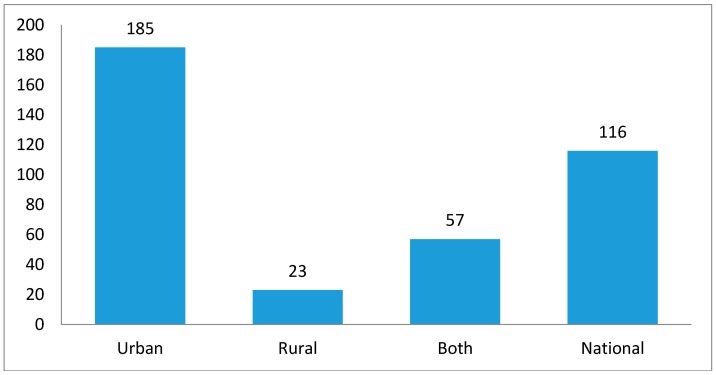
Representativeness of Data for Tobacco Control Research in SSA, (up to February 2017; *n* = 381).

Data on location of the study population were retrieved for 381 publications (85.23%; Figure 6). Among those studies, 185 (48.56%) were conducted in urban settings, 23 (6.04%) were in rural settings, and 57 (14.96%) represented both urban and rural settings. Additionally, 116 (30.45%) of the publications used national level data.

#### 3.6.3. Study Population

**Figure 7:**
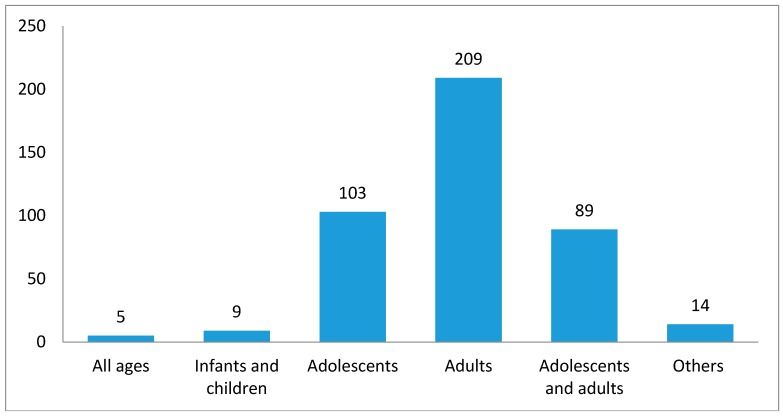
Characteristics of study population for Tobacco Control Research in SSA, (up to February 2017; *n* = 429).

Study population could be characterized for 429 studies. Out of these studies, the majority of the studies were undertaken among adults (*n* = 209, 48.72%; Figure 7), followed by adolescents (*n* = 103, 24.01%; Figure 6). Only nine studies were completed among infants and children (2.10%). Five studies (1.17%) used all age groups, while 89 (20.75%) were based on both adolescents and adults. Additionally, 14 studies (4.04%) collected data from non-human subjects (e.g., cigarettes, snuffs, and documents).

## 4. Discussion

As of June 2018, only two of the 47 the countries in the WHO African region have not ratified the WHO FCTC to implement tobacco control policies and programs in their respective countries. Yet, WHO FCTC Secretariat’s global progress reports on the implementation on the WHO FCTC [45,76,77,78,82,86] and other research studies [19,80] suggest that tobacco control in SSA lags behind other regions of the world. Also, it has been identified that progress in tobacco control in SSA has been confronted with inadequate capacity for research and the low priority given to tobacco control as a health problem [20,21]. Since scientific evidence is central for tobacco control [15,66,67,68], we conducted a systematic review of tobacco control research in SSA, with focus on the research methods to inform tobacco regulatory science and policy and advocacy initiatives in the region.

Overall, 447 peer-reviewed studies on SSA were retrieved for this study, with the first publication occurring in 1968. Research publications on tobacco control were low until 2006, which is after the WHO FCTC came into force as an international treaty in 2005 when there was an upsurge of publications per year. The rapid increase in tobacco control research in SSA between 2003 and 2016 may have been spurred by the WHO FCTC as scientific evidence is essential for tobacco control [15,66,67,68]. In particular, the evidence from this systematic review suggests that the Global Tobacco Surveillance System (GTSS), a collaborative project by organizations, including the WHO, CDC, and Canada Public Institute that was created in 1999 [87,88] has spurred tobacco control research in SSA. Indeed, 34 of the 447 studies relied on data from the Global Youth Tobacco Survey. While this trend of tobacco control research in SSA provides rationale for more investment into research to generate local/domestic data because policymakers are more receptive to such data for developing policies/programs [22,41], the inadequate capacity for tobacco control research needs urgent attention as research training plays a critical role in stopping the global tobacco epidemic [89]. The fact that at least 31% of the peer-reviewed publications examined in this study involved South Africa, where the research infrastructure is well developed, reinforces the point that there is the urgent need for developing tobacco control research capacity in countries across SSA in order to attain the commitment to implement the WHO FCTC and reduce non-communicable diseases (NCDs)-related mortality.

An examination of the research design and methods of the peer-reviewed publications showed that 95% of these studies utilized observational study design and 5% utilized randomized trials. This indicates that, based on the GRADE Guidelines (http://www.gradeworkinggroup.org/#pub), the evidence for tobacco control research in SSA is generally of “low” or “very low” quality, suggesting more investment into high quality research such as randomized trials and cohort studies. This examination further revealed that quantitative research has dominated tobacco control research in SSA, accounting for about 91% of the total publications in the region. This suggests the need for more qualitative and mixed method studies to provide additional deeper insights into the findings from the quantitative studies. Moreover, it was found that research in SSA is primarily cross-sectional in scope, accounting for 81% of all peer-reviewed studies. Additionally, about one-third (38.53%) of studies in SSA have depended on data using subjective self-reports. These kinds of research can be supplemented with investment in research that utilize objective measures as a means to overcome recall, social desirability, and non-response biases.

The findings from this study are consistent with a systemic review of NCD research in SSA [90,91] that shows very few longitudinal studies such as cohort study on tobacco control (retrospective or prospective) have been conducted in SSA. Although cross-sectional studies provide scientific evidence for tobacco control, they can only establish associations and are limited by the inability to make causal claims. This phenomenon limits the ability to holistically understand the etiology of tobacco use and policies/programs to prevent and control user behavior. In this respect, longitudinal studies are needed to strengthen the scientific basis for tobacco control and empower advocates with locally or domestically-generated data. This could potentially accelerate the progress of tobacco control to help prevent the anticipated increase in tobacco use by 37% [8] by 2025 [8] and stop NCDs from becoming the leading causes of death by 2030 [92].

Similar to any systematic review, this study has limitations. Particularly, we utilized four databases to retrieve the 447 peer-reviewed studies from 26 SSA countries, including Nigeria and South Africa, the first and fifth most populous countries, respectively, published up to February 2017 for a deeper examination of the research methods. In addition, we only reviewed papers that were written in English although some countries in this region do not use English as their primary language. It is possible that we may have missed non-English peer-reviewed publications; however, we believe that this number might be small and may have negligible influence on this systematic review. This study also did not examine other components of a peer-reviewed study or synthesize the findings as the focus was on the methodologies for research in SSA. Further, the study did not include synthesizing information on grey literature as the focus was on peer-reviewed scientific publications and future studies should include such information. Nevertheless, this is the first comprehensive review of the research methods of peer-reviewed studies in SSA that presents strong evidence that tobacco control research involving SSA is sparse, the type of design for studies is generally of “low” or “very low” quality (mostly observational studies, there is no known ongoing longitudinal study in SSA such as cohort studies), and the WHO FCTC may have spurred tobacco control research in the region.

## 5. Conclusions

This systematic review suggests that SSA is still a “research desert” with a total of 447 peer-reviewed articles published over the past 50 years (1968–2017). However, the number of studies published yearly has continued to increase, particularly after the adoption of the WHO FCTC. Governments in SSA have committed to implementing the WHO FCTC and reducing NCDs-related mortality. Research is central to achieving these public health goals. This review found that, while the WHO FCTC may have spurred tobacco control research in SSA, the studies are primarily cross-sectional in nature with reliance on survey data. This suggests that consideration should be given to more rigorous, robust, and high-quality studies such as RCTs, longitudinal, and cohort studies as they are critically needed for the SSA region. Additionally, more qualitative and mixed-methods research is need as these studies provide in-depth information for research on a subject-matter where SSA seems to be in a nascent stage. The central challenge to tobacco control research, however, has been the weak capacity, suggesting the need for more investments in research training. A strong tobacco control research in SSA has the potential to reverse the increasing trends in tobacco use and NCDs through policy and programmatic changes, and this systematic review suggests that, except for South Africa, this is lacking across countries in SSA. As such, there is a critical need for investment in tobacco control research and building research capacity and training in research methodology.

## Supplementary Materials

The following are available online at http://www.mdpi.com/1660-4601/15/12/2732/s1, Table S1: List of peer-reviewed publications and data abstraction (up to February 2017).
