# Supplementary material for: The Progress of Tobacco Control Research in Sub-Saharan Africa in the Past 50 Years: A Systematic Review of the Design and Methods of the Studies
Source: Int J Environ Res Public Health. 2018 Dec 4;15(12):2732. doi: 10.3390/ijerph15122732 (PMC6313754; doi:10.3390/ijerph15122732)
Supplement: Supplementary file 1 [file ijerph-15-02732-s001.docx]

| **Table S1: List of peer-reviewed publications and data abstraction (up to February 2017).** | | | | | | | | | | | | | | | | | |
| --- | --- | --- | --- | --- | --- | --- | --- | --- | --- | --- | --- | --- | --- | --- | --- | --- | --- |
| SN | Author | Title | Country | Year | Analytical sample | Sample Characteristics | Type of Sampling | Mean age | Male% | Place of recruitment | Location | Research Study design | Type of research study | Research data characteristics | Power | Eligibility of Participants | Use of GYTS |
| 1 | Gelfand M, Graham AJ, Lightman E. | Carcinoma of bronchus and the smoking habit in Rhodesian Africans. | Zimbabwe | 1968 | 64 | Adults | Purposive sampling | - | 100% | Health facility | Urban | Quantitative | Case control | Observation | No | Yes | No |
| 2 | Arya OP, Bennett FJ. | Smoking amongst university students in Uganda--an analysis of prevalence and attitudes. | Uganda | 1970 | 1328 | Adult students | - | - | - | Schools | - | Quantitative | Cross-sectional | Self-administered questionnaire | No | No | No |
| 3 | Bradshaw E, Schonland M. | Smoking, drinking and oesophageal cancer in African males of Johannesburg, South Africa. | South Africa | 1974 | 1260 | Adults aged 35 or more | Purposive sampling | - | 100% | Health facility | Urban | Quantitative | Case control | Interview | No | No | No |
| 4 | Elegbeleye OO, Femi-Pearse D. | Incidence and variables contributing to onset of cigarette smoking among secondary school children and medical students in Lagos, Nigeria. | Nigeria | 1976 | 2205 | Adolescent and adult students | Convenience sampling | - | 55.41% | Schools | Urban | Quantitative | Cross-sectional | Self-administered questionnaire | No | No | No |
| 5 | van der Burgh C. | Smoking behaviour of White, Black, Coloured and Indian South Africans. Some statistical data on a major public health hazard. | South Africa | 1979 | 14000 | Adults | Multistage stratified sampling | - | - | Community/household | National | Quantitative | Cross-sectional | - | No | No | No |
| 6 | Seftel HC. | The first published chemical analyses of smoke from South African cigarettes. | South Africa | 1979 | 69 brands | Cigarettes | Convenience sampling | - | - | Community/household | - | Quantitative | Cross-sectional | Observation | No | No | No |
| 7 | Ahmed Z, Abuhay M. | The prevalence of cigarette-smoking among secondary school children in Gondar City, Ethiopia. | Ethiopia | 1979 | 538 | Adolescents and youths aged 12 to 23 years | Systematic sampling | - | 47.40% | Schools | Urban | Quantitative | Cross-sectional | Self-administered questionnaire | No | No | No |
| 8 | Yach D. | Economic aspects of smoking in South Africa. | South Africa | 1982 | - | - | - | - | - | - | National | Quantitative | Cross-sectional (Economic analysis) | - | No | - | No |
| 9 | D'Hondt W, Vandewiele M. | Attitudes of Senegalese schoolgoing adolescents towards tobacco smoking. | Senegal | 1982 | 731 | Young adult students aged 17 to 21 years | - | - | 86% | Schools | Urban | Quantitative | Cross-sectional | Interview | No | No | No |
| 10 | Awotedu AA, Higenbottam TW, Onadeko BO. | Tar, nicotine, and carbon monoxide yields of some Nigerian cigarettes. | Nigeria | 1983 | 1000 adults and 140 cigarettes | Adults and Cigarettes | Random sampling | - | - | Community/household | - | Quantitative | Cross-sectional | Interview and Observation | No | No | No |
| 11 | Pobee JO, Larbi EB, Kpodonu J. | The profile of the African smoker: the Ghana smoking studies. | Ghana | 1984 | 4433 | Individuals aged more than 15 years | - | - | - | Community/household and workplace | - | Quantitative | Cross-sectional | - | No | No | No |
| 12 | Ballal SG. | Cigarette smoking and respiratory symptoms among Sudanese doctors. | Sudan | 1984 | 753 | Adults | Convenience sampling | - | 79.50% | Workplace | Both | Quantitative | Cross-sectional | Observation | No | No | No |
| 13 | Zein ZA, Admasu M, Tadesse M, Laeke N, Olango P, Rassu TA, Asfaw T. | Patterns of cigarette-smoking among Ethiopian medical and paramedical students. | Ethiopia | 1984 | 479 | Adult students | - | 21.2 | 81.60% | Schools | Urban | Quantitative | Cross-sectional | Self-administered questionnaire | No | No | No |
| 14 | Nevadomsky JJ. | Drug use among Nigerian university students: prevalence of self-reported use and attitudes to use. | Nigeria | 1985 | 295 | Adult students | - | - | - | Schools | Urban | Quantitative | Cross-sectional | Self-administered questionnaire | No | No | No |
| 15 | Gomo ZA. | The effect of age, sex, alcohol consumption and cigarette smoking on serum concentrations of lipids and apolipoproteins in Zimbabwean blacks. | Zimbabwe | 1986 | 829 | Adults | Random sampling | - | 71.05% | Workplace | Urban | Quantitative | Cross-sectional | Observation | No | No | No |
| 16 | Callander C, Rocke DA. | Smoking habits and attitudes of Durban metropolitan anaesthetists. | South Africa | 1986 | 102 | Adults | Purposive sampling | - | 86.27% | Workplace | Urban | Quantitative | Cross-sectional | Self-administered questionnaire | No | No | No |
| 17 | Harries AD, Chugh KS, Neumann T. | Smoking habits and disease patterns amongst hospital patients in north-east Nigeria. | Nigeria | 1986 | 2346 | Adults aged 18 to 86 years | Convenience sampling | - | 34.95% | Health facility | Urban | Quantitative | Cross-sectional | Self-administered questionnaire | No | No | No |
| 18 | Oleru UG. | Pulmonary impairment in a cotton textile factory in Nigeria: is lifetime alcohol intake with low cigarette smoking a confounding factor? | Nigeria | 1987 | 60 | Adults | Purposive sampling | - | - | Workplace | Urban | Quantitative | Cross-sectional | Observation | No | Yes | No |
| 19 | Onadeko BO, Awotedu AA, Onadeko MO. | Smoking patterns in students of higher institutions of learning in Nigeria. | Nigeria | 1987 | 2317 | Adult students | - | - | 63.88% | Schools | - | Quantitative | Cross-sectional | Self-administered questionnaire | No | No | No |
| 20 | Yach D, Joubert G. | Deaths related to smoking in South Africa in 1984 and projected deaths among coloureds and blacks in the year 2000. | South Africa | 1988 | - | Reported deaths of adults aged 35 years and more | - | - | - | - | National | Quantitative | Cross-sectional | Administrative data/document review | No | No | No |
| 21 | Hnizdo E, Sluis-Cremer GK. | Effect of tobacco smoking on the presence of asbestosis at postmortem and on the reading of irregular opacities on roentgenograms in asbestos-exposed workers. | South Africa | 1988 | 886 | Adults | Purposive sampling | - | - | Workplace | - | Quantitative | Case control | Observation | No | Yes | No |
| 22 | Baddeley GM, Schomer HH, Albrecht CF. | Nicotine gum and psychological support in smoking cessation. A pilot study in South Africa. | South Africa | 1988 | 23 | Adults | Convenience sampling | - | 56.52% | Community/household | - | Quantitative | Non-randomized controlled intervention study | Observation | No | No | No |
| 23 | Ihezue UH. | Drug abuse among medical students at a Nigerian university: Part 1. Prevalence and pattern of use. | Nigeria | 1988 | 728 | - | Purposive sampling | - | 83.37% | Schools | Urban | Quantitative | Cross-sectional | Self-administered questionnaire | No | No | No |
| 24 | McIntyre DE, Taylor SP. | Economic aspects of smoking in South Africa. | South Africa | 1989 | - | - | - | - | - | - | National | Quantitative | Cross-sectional (Economic analysis) | - | No | - | No |
| 25 | Elbeshir EI, Abeen HA, Idris AM, Abbas K. | Snuff dipping and oral cancer in Sudan: a retrospective study. | Sudan | 1989 | 78 | Patients aged 15 to 85 years | Purposive sampling | 60.5 | - | Health facility | Urban | Quantitative | Cross-sectional | Interview | No | No | No |
| 26 | Strebel P, Kuhn L, Yach D. | Determinants of cigarette smoking in the black township population of Cape Town. | South Africa | 1989 | 1993 | Adolescents and adults | Random cluster sampling | 13.8 for school pupils and 34.5 for adults | 46.35% in school and 42.19 among adults | School and community/household | Urban | Quantitative | Cross-sectional | Interview | No | No | No |
| 27 | Pela OA. | Patterns of adolescent psychoactive substance use and abuse in Benin City, Nigeria. | Nigeria | 1989 | 320 | Adolescents and adults aged 14 to 25 years | - | - | 62.50% | Community/household | Urban | Quantitative | Cross-sectional | Observation | No | No | No |
| 28 | Obot IS. | The use of tobacco products among Nigerian adults: a general population survey. | Nigeria | 1990 | 1271 | Adults | Multistage random sampling | - | 89.50% | Community/household | Both | Quantitative | Cross-sectional | Interview | No | No | No |
| 29 | Vermaak WJ, Ubbink JB, Barnard HC, Potgieter GM, van Jaarsveld H, Groenewald AJ. | Vitamin B-6 nutrition status and cigarette smoking. | South Africa | 1990 | 286 | Adults | Convenience sampling | - | 100% | Health facility | - | Quantitative | Cross-sectional | Observation | No | No | No |
| 30 | Hnizdo E, Baskind E, Sluis-Cremer GK. | Combined effect of silica dust exposure and tobacco smoking on the prevalence of respiratory impairments among gold miners. | South Africa | 1990 | 2692 | Adults aged 45 to 54 years | Purposive sampling | - | - | Workplace | - | Quantitative | Cross-sectional | Observation | No | Yes | No |
| 31 | Hnizdo E. | Combined effect of silica dust and tobacco smoking on mortality from chronic obstructive lung disease in gold miners. | South Africa | 1990 | 462 | Adults aged 45 to 54 years | Purposive sampling | - | - | Workplace | - | Quantitative | Nested case control study | Observation | No | Yes | No |
| 32 | Anderson R, Theron AJ, Richards GA, Myer MS, van Rensburg AJ. | Passive smoking by humans sensitizes circulating neutrophils | South Africa | 1991 | 36 | Adults | Purposive sampling | - | 38.89% | - | - | Quantitative | Cross-sectional | Observation | No | No | No |
| 33 | Idris AM, Nair J, Ohshima H, Friesen M, Brouet I, Faustman EM, Bartsch H. | Unusually high levels of carcinogenic tobacco-specific nitrosamines in Sudan snuff (toombak). | Sudan | 1991 | - | Snuffs | Purposive sampling | - | - | Community/household | - | Quantitative | Cross-sectional | Observation | No | No | No |
| 34 | Martin G, Steyn K, Yach D. | Beliefs about smoking and health and attitudes toward tobacco control measures. | South Africa | 1992 | 2006 | Adults aged 18 years and more | Multistage cluster sampling | - | 44.70% | Community/household | National | Quantitative | Cross-sectional | Interview | No | No | No |
| 35 | Idris AM, Nair J, Friesen M, Ohshima H, Brouet I, Faustman EM, Bartsch H. | Carcinogenic tobacco-specific nitrosamines are present at unusually high levels in the saliva of oral snuff users in Sudan. | Sudan | 1992 | 10 | Adults aged 18 to 70 years | Purposive sampling | - | 100% | - | Urban | Quantitative | Cross-sectional | Observation | No | No | No |
| 36 | Orie NN, Ibanga IN. | The effect of nocotiana tabacum (snuff) on blood pressure and pulse rate of Nigerians. | Nigeria | 1992 | 33 | Adults aged 20 to 68 years | Convenience sampling | - | 100% | Community/household | Urban | Quantitative | Cross-sectional | Observation | No | Yes | No |
| 37 | Munodawafa D, Marty PJ, Gwede C. | Drug use and anticipated parental reaction among rural school pupils in Zimbabwe. | Zimbabwe | 1992 | 285 | School going adolescents | Convenience sampling | 15.9 | 47.37% | Schools | Rural | Quantitative | Cross-sectional | Self-administered questionnaire | No | No | No |
| 38 | Adelekan ML, Abiodun OA, Obayan AO, Oni G, Ogunremi OO. | Prevalence and pattern of substance use among undergraduates in a Nigerian University. | Nigeria | 1992 | 636 | Adult students | Multistage stratified sampling | 23 | 69% | Schools | Urban | Quantitative | Cross-sectional | Self-administered questionnaire | No | No | No |
| 39 | Kurtz ME, Azikiwe U, Kurtz JC. | Nigerian urban married women's perceptions of exposure to secondary tobacco smoke. | Nigeria | 1993 | 249 | Adults | Convenience sampling | - | 0% | Community/household | Urban | Quantitative | Cross-sectional | Self-administered questionnaire | No | No | No |
| 40 | Adelekan ML, Abiodun OA, Imouokhome-Obayan AO, Oni GA, Ogunremi OO. | Psychosocial correlates of alcohol, tobacco and cannabis use: findings from a Nigerian university. | Nigeria | 1993 | 636 | Adolescents and adult students aged 11 to 40 years | - | - | 69.18% | Schools | Urban | Quantitative | Cross-sectional | Self-administered questionnaire | No | No | No |
| 41 | Flisher AJ, Ziervogel CF, Chalton DO, Leger PH, Robertson BA. | Risk-taking behaviour of Cape Peninsula high-school students. Part III. Cigarette smoking. | South Africa | 1993 | 7340 | High school students | Cluster sampling | - | - | Schools | Urban | Quantitative | Cross-sectional | Self-administered questionnaire | No | No | No |
| 42 | Yach D, Paterson G. | Tobacco advertising in South Africa with specific reference to magazines. | South Africa | 1994 | 30 | Magazines | Purposive sampling | - | - | - | - | Quantitative | Cross-sectional | Administrative data/document review | No | No | No |
| 43 | Idris AM, Prokopczyk B, Hoffmann D. | Toombak: a major risk factor for cancer of the oral cavity in Sudan. | Sudan | 1994 | 5500 | Adults | Multistage stratified sampling | - | 52.14% | Community/household | Both | Quantitative | Cross-sectional | Interview | No | No | No |
| 44 | Steyn K, Bourne LT, Jooste PL, Fourie JM, Lombard CJ, Yach D. | Smoking in the black community of the Cape Peninsula, South Africa. | South Africa | 1994 | 976 | Individuals aged 15 to 64 years | Multistage stratified sampling | - | - | Community/household | Urban | Quantitative | Cross-sectional | Observation | No | No | No |
| 45 | Murphy SE, Carmella SG, Idris AM, Hoffmann D. | Uptake and metabolism of carcinogenic levels of tobacco-specific nitrosamines by Sudanese snuff dippers. | Sudan | 1994 | 7 | Adults | Purposive sampling | - | - | Health facility | Urban | Quantitative | Cross-sectional | Observation | No | No | No |
| 46 | Abiodun OA, Adelekan ML, Ogunremi OO, Oni GA, Obayan AO. | Psychosocial correlates of alcohol, tobacco and cannabis use amongst secondary school students in Ilorin, Nigeria. | Nigeria | 1994 | 1041 | School going adolescents and young adults aged 10 to 25 years | - | - | 55.52% | Schools | - | Quantitative | Cross-sectional | Self-administered questionnaire | No | No | No |
| 47 | Acuda SW, Eide AH. | Epidemiological study of drug use in urban and rural secondary schools in Zimbabwe. | Zimbabwe | 1994 | 2783 | School going adolescents | Multistage stratified sampling | 15.5 | 44% | Schools | Both | Quantitative | Cross-sectional | Self-administered questionnaire | No | No | No |
| 48 | Abiodun OA, Adelekan ML, Ogunremi OO, Oni GA, Obayan AO. | Pattern of substance use amongst secondary school students in Ilorin, northern Nigeria. | Nigeria | 1994 | 1041 | School going adolescents and young adults aged 10 to 25 years | Multistage stratified sampling | 16.8 | 56.50% | Schools | Both | Quantitative | Cross-sectional | Self-administered questionnaire | No | No | No |
| 49 | Ferrinho P. | Smoking in a poor, rapidly expanding urban community of South Africa. | South Africa | 1994 | 3108 | Children, Adolescent, and adults | Cluster sampling | - | 36.54% | Community/household | Urban | Quantitative | Cross-sectional | Interview | No | No | No |
| 50 | Idris AM, Ahmed HM, Mukhtar BI, Gadir AF, el-Beshir EI. | Descriptive epidemiology of oral neoplasms in Sudan 1970-1985 and the role of toombak. | Sudan | 1995 | 1885 | Adults | - | - | - | - | - | Quantitative | Cross-sectional | Administrative data/document review | No | No | No |
| 51 | Idris AM, Ahmed HM, Malik MO. | Toombak dipping and cancer of the oral cavity in the Sudan: a case-control study. | Sudan | 1995 | 3670 | Cancer patients (including all age groups) | Purposive sampling | - | 63.43% | Health facility | Urban | Quantitative | Case control | Interview | No | Yes | No |
| 52 | Siziya S, Marufu T, Matchaba-Hove RB. | Relationship of casual blood pressure to smoking, education and occupation in a high density town near Harare, Zimbabwe. | Zimbabwe | 1995 | 973 | Individuals aged 5 years and more | - | - | - | Community/household | - | Quantitative | Cross-sectional | Observation | No | No | No |
| 53 | Macigo FG, Mwaniki DL, Guthua SW. | The association between oral leukoplakia and use of tobacco, alcohol and that based on relative risks assessment in Kenya | Kenya | 1995 | 85 cases and 141 controls | Adults | Cluster sampling | | 47.40% | Community/household | - | Quantitative | Cross-sectional | Observation | Yes | Yes | No |
| 54 | Abul Bangura S, Lisk RD. | Tobacco and cannabis smoking in secondary school pupils in Bo, Sierra Leone. | Sierra Leone | 1995 | 713 | School going adolescents and young adults aged 10 to 27 years | Multistage stratified sampling | 17.6 | 56.80% | Schools | Urban | Quantitative | Cross-sectional | Self-administered questionnaire | No | No | No |
| 55 | Eide AH, Acuda SW. | Drug use among secondary school students in Zimbabwe. | Zimbabwe | 1995 | 2581 | School going adolescents and youths aged 12 to 21 years | Multistage stratified sampling | - | 56% | Schools | Both | Quantitative | Cross-sectional | Self-administered questionnaire | No | No | No |
| 56 | Macigo FG, Mwaniki DL, Guthua SW. | Influence of dose and cessation of kiraiku, cigarettes and alcohol use on the risk of developing oral leukoplakia. | Kenya | 1996 | 226 | Adolescents and adults aged 15 years and more | Multistage cluster sampling | - | - | Community/household | Rural | Quantitative | Case control | Interview | No | No | No |
| 57 | Ehrlich RI, Du Toit D, Jordaan E, Zwarenstein M, Potter P, Volmink JA, Weinberg E. | Risk factors for childhood asthma and wheezing. Importance of maternal and household smoking. | South Africa | 1996 | 576 | Children aged 7 to 8 years | Multistage cluster sampling | 7.6 | 51.20% | Schools | Urban | Quantitative | Case control | Observation | No | Yes | No |
| 58 | Idris AM, Warnakulasuriya KA, Ibrahim YE, Nielsen R, Cooper D, Johnson NW. | Toombak-associated oral mucosal lesions in Sudanese show a low prevalence of epithelial dysplasia. | Sudan | 1996 | 281 | Individuals aged 17 to 97 years | Random sampling | 48.4 | 82.08% | Community/household | - | Quantitative | Cross-sectional | Observation | No | No | No |
| 59 | Ibrahim SO, Johannessen AC, Idris AM, Hirsch JM, Vasstrand EN, Magnusson B, Nilsen R. | Immunohistochemical detection of p53 in non-malignant and malignant oral lesions associated with snuff dipping in the Sudan and Sweden. | Sudan | 1996 | 28 | Adults | - | - | - | Health facility | - | Quantitative | Cross-sectional | Observation | No | No | No |
| 60 | Gill GV, Rolfe M, MacFarlane IA, Huddle KR. | Smoking habits of black South African patients with diabetes mellitus. | South Africa | 1996 | 223 | Adults | Convenience sampling | - | - | Health facility | Urban | Quantitative | Cross-sectional | Observation | No | No | No |
| 61 | Lazarus P, Idris AM, Kim J, Calcagnotto A, Hoffmann D. | p53 mutations in head and neck squamous cell carcinomas from Sudanese snuff (toombak) users. | Sudan | 1996 | 4 | Adults | Purposive sampling | - | - | Health facility | Urban | Quantitative | Cross-sectional | Observation | No | Yes | No |
| 62 | Ndom RJ, Adelekan ML. | Psychosocial correlates of substance use among undergraduates in Ilorin University, Nigeria. | Nigeria | 1996 | 859 | Students aged 15 to 40 years | Multistage stratified sampling | 23.7 | 73% | Schools | - | Quantitative | Cross-sectional | Self-administered questionnaire | No | No | No |
| 63 | Khan N, Arnott R. | Substance use among rural secondary schools in Zimbabwe: patterns and prevalence. | Zimbabwe | 1996 | 917 | School going adolescents | Random sampling | - | 64.40% | Schools | Rural | Quantitative | Cross-sectional | Self-administered questionnaire | No | No | No |
| 64 | Eide AH, Acuda SW. | Adolescents' drug use in Zimbabwe--comparing two recent studies. | Zimbabwe | 1996 | 5642 | School going adolescents and adults aged 11 to 23 years | Multistage stratified sampling | - | - | Schools | Both | Quantitative | Cross-sectional (data collected in multiple time points) | Self-administered questionnaire | No | No | No |
| 65 | Reddy P, Meyer-Weitz A, Yach D. | Smoking status, knowledge of health effects and attitudes towards tobacco control in South Africa. | South Africa | 1996 | 2238 | Adults aged 18 and more | Multistage cluster sampling | - | 43% | Community/household | National | Quantitative | Cross-sectional | Self-administered questionnaire | No | Yes | No |
| 66 | Acuda SW, Sebit MB. | Prevalence of psychoactive substance use among psychiatric in-patients in Harare, Zimbabwe. | Zimbabwe | 1997 | 194 | Patients aged 16 to 55 years | Systematic sampling | 33.5 | 43.80% | Health facility | Urban | Quantitative | Cross-sectional | Interview | No | Yes | No |
| 67 | Steyn K, Yach D, Stander I, Fourie JM. | Smoking in urban pregnant women in South Africa. | South Africa | 1997 | 394 | Adults | Convenience sampling | 23 | 0% | Health facility | Urban | Quantitative | Cross-sectional | Self-administered questionnaire | No | No | No |
| 68 | Idris AM, Ibrahim YE, Warnakulasuriya KA, Cooper DJ, Johnson NW, Nilsen R. | Toombak use and cigarette smoking in the Sudan: estimates of prevalence in the Nile state. | Sudan | 1998 | 21594 | Children, adolescents and adults | Multistage cluster sampling | - | 51.30% | Community/household | Both | Quantitative | Cross-sectional | Interview | No | Yes | No |
| 69 | Ibrahim SO, Warnakulasuriya KA, Idris AM, Hirsch JM, Johnson NW, Johannessen AC. | Expression of keratin 13, 14 and 19 in oral hyperplastic and dysplastic lesions from Sudanese and Swedish snuff-dippers: association with human papillomavirus infection. | Sudan | 1998 | 49 | Adults | - | - | - | Health facility | - | Quantitative | Cross-sectional | Observation | No | No | No |
| 70 | Idris AM, Warnakulasuriya KA, Ibrahim YE, Hartley R, Paterson K, Patel B, Nilsen R, Johnson NW. | Characterization of an amorphous deposit in the lamina propria in oral snuff users in the Sudan as collagen. | Sudan | 1998 | 25 | Adults | - | - | - | Health facility | - | Quantitative | Cross-sectional | Observation | No | No | No |
| 71 | Ibrahim SO, Bertelsen B, Kalvenes MB, Idris AM, Vasstrand EN, Nilsen R, Johannessen AC. | Expression of keratin 13, 14 and 19 in oral squamous cell carcinomas from Sudanese snuff dippers: lack of association with human papillomavirus infection. | Sudan | 1998 | 28 | - | Purposive sampling | - | - | Health facility | - | Quantitative | Cross-sectional | Observation | No | No | No |
| 72 | Osim EE, Musabayane CT, Mufunda J. | Lung function of Zimbabwean farm workers exposed to flue curing and stacking of tobacco leaves. | Zimbabwe | 1998 | 50 | Adults | Purposive sampling | - | 100% | Workplace | - | Quantitative | Cross-sectional | Observation | No | No | No |
| 73 | Laforge RG, Velicer WF, Levesque DA, Fava JL, Hill DJ, Schofield PE, Fan D, De Vries H, Shisana WO, Conner M. | Measuring support for tobacco control policy in selected areas of six countries. | South Africa | 1998 | 291 | Adults | - | - | 47.90% | Schools | Urban | Quantitative | Cross-sectional | Self-administered questionnaire | No | No | No |
| 74 | Bayat M, Pillay BJ, Cassimjee MH. | Cigarette smoking behavior among South african Indian high school students. | South Africa | 1998 | 325 | Adults | Convenience sampling | 17.2 | 46.90% | Schools | Both | Quantitative | Cross-sectional | Self-administered questionnaire | No | No | No |
| 75 | Mzileni O, Sitas F, Steyn K, Carrara H, Bekker P. | Lung cancer, tobacco, and environmental factors in the African population of the Northern Province, South Africa. | South Africa | 1999 | 728 | Adults | Purposive sampling | - | 64.69% | Health facility | Urban | Quantitative | Case control | Interview | No | No | No |
| 76 | Jones AM, Kirigia JM. | Health knowledge and smoking among South African women. | South Africa | 1999 | 3489 | Adults | Multistage stratified sampling | - | 0% | Community/household | National | Quantitative | Cross-sectional | Interview | No | No | No |
| 77 | Peltzer K. | Smokeless tobacco use among urban white and black South Africans | South Africa | 1999 | 300 | Adults | Systematic random sampling | - | - | Community/household | Urban | Quantitative | Cross-sectional | Interview | No | No | No |
| 78 | Ibrahim SO, Vasstrand EN, Johannessen AC, Idris AM, Magnusson B, Nilsen R, Lillehaug JR. | Mutations of the p53 gene in oral squamous-cell carcinomas from Sudanese dippers of nitrosamine-rich toombak and non-snuff-dippers from the Sudan and Scandinavia. | Sudan | 1999 | 39 | - | - | - | - | - | - | Quantitative | Cross-sectional | Observation | No | No | No |
| 79 | Smuts CM, Tichelaar HY, Dhansay MA, Faber M, Smith J, Kirsten GF. | Smoking and alcohol use during pregnancy affects preterm infants' docosahexaenoic acid (DHA) status. | South Africa | 1999 | 40 | Newborns (Infants) | Purposive sampling | - | - | Health facility | - | Quantitative | Cross-sectional | Observation | No | Yes | No |
| 80 | Jordaan ER, Ehrlich RI, Potter P. | Environmental tobacco smoke exposure in children: household and community determinants. | South Africa | 1999 | 575 | Children (6-11 years) | Multistage cluster sampling | 7.6 | - | Schools | Urban | Quantitative | Cross-sectional | Observation | No | Yes | No |
| 81 | Eide AH, Butau T, Acuda SW. | Adolescent drug use in Zimbabwe assessed by their teachers. | Zimbabwe | 1999 | 442 | Adults | - | - | 50% | Workplace | Both | Quantitative | Cross-sectional | Self-administered questionnaire | No | No | No |
| 82 | Eide AH, Butau T, Acuda SW. | Use of alcohol and tobacco among secondary school teachers in Zimbabwe. | Zimbabwe | 1999 | 426 | Adults | Multistage stratified sampling | - | 49.08% | Workplace | Both | Quantitative | Cross-sectional | Self-administered questionnaire | No | No | No |
| 83 | Chantornvong S, Collin J, Dodgson R, Lee K, McCargo D, Seddon D, Vaughan P, Woelk G. | Political economy of tobacco control in low-income and middle-income countries: lessons from Thailand and Zimbabwe. Global Analysis Project Team. | Zimbabwe | 2000 | - | Documents and adults | - | - | - | - | - | Qualitative | Case study | Administrative data/document review and interview | No | No | No |
| 84 | Loro LL, Vintermyr OK, Ibrahim SO, Idris AM, Johannessen AC. | Apoptosis and expression of Bax and Bcl-2 in snuff- and non-snuff associated oral squamous cell carcinomas. | Sudan | 2000 | 19 | - | - | - | - | Health facility | - | Quantitative | Cross-sectional | Observation | No | No | No |
| 85 | Peltzer K, Cherian L. | Substance use among urban and rural secondary school pupils in South Africa. | South Africa | 2000 | 400 | Adults | - | - | 44% | Schools | Both | Quantitative | Cross-sectional | Self-administered questionnaire | No | No | No |
| 86 | Warren CW, Riley L, Asma S, Eriksen MP, Green L, Blanton C, Loo C, Batchelor S, Yach D. | Tobacco use by youth: a surveillance report from the Global Youth Tobacco Survey project. | South Africa and Zimbabwe | 2000 | South Africa: 7074, Zimbabwe: 2594 | School going adolescents aged 13 to 15 years | Multistage cluster sampling | - | - | Schools | National | Quantitative | Cross-sectional | Self-administered questionnaire | No | Yes | Yes |
| 87 | Peltzer K, Phaswana N, Malaka D. | Smokeless tobacco use among adults in the Northern Province of South Africa: qualitative data from focus groups. | South Africa | 2001 | 72 | Adults | Convenience sampling | - | 45.83% | Community/household | - | Qualitative | Thematic analysis | Focus group discussion | No | No | No |
| 88 | Macigo FG, Mwaniki DL, Guthua SW, Njeru EK. | Influence of cigarette filters on the risk of developing oral leukoplakia in a Kenyan population. | Kenya | 2001 | 226 | Adolescents and adults aged 15 years and more | Multistage cluster sampling | - | - | Community/household | Rural | Quantitative | Case control | Interview | No | No | No |
| 89 | Peltzer K. | Tobacco smoking in Black and White South Africans. | South Africa | 2001 | 500 | Adults aged 18 years and more | Systematic random sampling | - | - | Community/household | Urban | Quantitative | Cross-sectional | Interview | No | No | No |
| 90 | Steyn K, Hoffman M, Levitt NS, Lombard CJ, Fourie JM. | Community-based tobacco control program: the Mamre study, a demonstration project. | South Africa | 2001 | 2212 | Adolescents and adults aged 15 years and more | Multistage stratified sampling | - | - | Community/household | Urban | Quantitative | Interventional study | Observation | No | No | No |
| 91 | Walraven GE, Nyan OA, Van Der Sande MA, Banya WA, Ceesay SM, Milligan PJ, McAdam KP. | Asthma, smoking and chronic cough in rural and urban adult communities in The Gambia | Gambia | 2001 | 5389 | Adolescents and adults aged 15 years or more | Cluster sampling | - | 41.34% | Community/household | Both | Quantitative | Cross-sectional | Self-administered questionnaire | No | No | No |
| 92 | Adelekan ML, Makanjuola AB, Ndom RJ, Fayeye JO, Adegoke AA, Amusan O, Idowu AI. | 5-yearly monitoring of trends of substance use among secondary school students in Ilorin, Nigeria, 1988-1998. | Nigeria | 2001 | 727 | School going adolescents | Multistage stratified sampling | - | 58% | Schools | Urban | Quantitative | Cross-sectional (data collected at multiple time points) | Self-administered questionnaire | No | No | No |
| 93 | van Walbeek C. | Recent trends in smoking prevalence in South Africa--some evidence from AMPS data. | South Africa | 2002 | - | Individuals aged 16 years and more | - | - | - | Community/household | National | Quantitative | Cross-sectional (data collected in multiple time points) | - | No | No | No |
| 94 | WHO ASSIST Working Group.. | The Alcohol, Smoking and Substance Involvement Screening Test (ASSIST): development, reliability and feasibility. | Zimbabwe | 2002 | 236 | Adults | Convenience sampling | 34 | 53.60% | Health facility | - | Quantitative | Cross-sectional | Interview | No | Yes | No |
| 95 | Bovet P, Ross AG, Gervasoni JP, Mkamba M, Mtasiwa DM, Lengeler C, Whiting D, Paccaud F. | Distribution of blood pressure, body mass index and smoking habits in the urban population of Dar es Salaam, Tanzania, and associations with socioeconomic status. | Tanzania | 2002 | 9254 | Adults aged 25 to 65 years | Census | 35.5 | 39% | Community/household | Urban | Quantitative | Cross-sectional | Observation | No | Yes | No |
| 96 | Steyn K, Bradshaw D, Norman R, Laubscher R, Saloojee Y. | Tobacco use in South Africans during 1998: the first demographic and health survey. | South Africa | 2002 | 13826 | Adolescents and adults aged more than 15 years | Multistage cluster sampling | - | 41.61% | Community/household | National | Quantitative | Cross-sectional | Observation | No | Yes | No |
| 97 | Ibrahim SO, Lillehaug JR, Dolphine O, Johnson NW, Warnakulasuriya KA, Vasstrand EN. | Mutations of the cell cycle arrest gene p21WAF1, but not the metastasis-inducing gene S100A4, are frequent in oral squamous cell carcinomas from Sudanese toombak dippers and non-snuff-dippers from the Sudan, Scandinavia, USA and UK. | Sudan | 2002 | 39 | - | - | - | - | Health facility | - | Quantitative | Cross-sectional | Observation | No | No | No |
| 98 | Steptoe A, Wardle J, Cui W, Baban A, Glass K, Tsuda A, Vinck J. | An international comparison of tobacco smoking, beliefs and risk awareness in university students from 23 countries. | South Africa | 2002 | 786 | Individuals aged 17-30 years | - | - | 46.69% | Schools | National | Quantitative | Cross-sectional | Self-administered questionnaire | No | No | No |
| 99 | Fatoye FO, Morakinyo O. | Substance use amongst secondary school students in rural and urban communities in south western Nigeria. | Nigeria | 2002 | 562 | School going adolescents and adults aged 15 to 21 years | Multistage cluster sampling | 17 | - | Schools | Both | Quantitative | Cross-sectional | Self-administered questionnaire | No | No | No |
| 100 | Morojele NK, Flisher AJ, Muller M, Ziervogel CF, Reddy P, Lombard CJ. | Measurement of risk and protective factors for drug use and anti-social behavior among high school students in South Africa. | South Africa | 2002 | 123 | School going adolescents | Purposive sampling | 14.33 | 74% | Schools | Urban | Quantitative | Cross-sectional | Self-administered questionnaire | No | No | No |
| 101 | Peltzer K, Malaka DW, Phaswana N. | Sociodemographic factors, religiosity, academic performance, and substance use among first-year university students in South Africa. | South Africa | 2002 | 799 | University students aged 16 to 49 years | Random sampling | 20.1 | 55.20% | Schools | Both | Quantitative | Cross-sectional | Self-administered questionnaire | No | No | No |
| 102 | Jagoe K, Edwards R, Mugusi F, Whiting D, Unwin N. | Tobacco smoking in Tanzania, East Africa: population based smoking prevalence using expired alveolar carbon monoxide as a validation tool. | Tanzania | 2002 | 605 | Adolescents and adults aged 15 and more | Stratified random sampling | 42.9 | 52.52% | Community/household | Urban | Quantitative | Cross-sectional | Observation | No | Yes | No |
| 103 | Panday S, Reddy SP, Bergström E. | A qualitative study on the determinants of smoking behaviour among adolescents in South Africa. | South Africa | 2003 | 60 | Adolescents aged 13 and 14 years | Purposive sampling | - | - | Schools | Both | Qualitative | Grounded theory | Focus group discussion | No | No | No |
| 104 | Morakinyo J, Odejide AO. | A community based study of patterns of psychoactive substance use among street children in a local government area of Nigeria. | Nigeria | 2003 | 180 | Adolescents aged 8 to 18 years | Multistage cluster sampling | 14.6 | 96.70% | Community/household | Urban | Quantitative | Cross-sectional | Interview | No | Yes | No |
| 105 | Ahmed HG, Idris AM, Ibrahim SO. | Study of oral epithelial atypia among Sudanese tobacco users by exfoliative cytology. | Sudan | 2003 | 300 | Adults aged 18 years and more | Purposive sampling | - | 97% | Community/household | Urban | Quantitative | Cross-sectional | Observation | No | No | No |
| 106 | King G, Flisher AJ, Mallett R, Graham J, Lombard C, Rawson T, Morojele NK, Muller M. | Smoking in Cape Town: community influences on adolescent tobacco use. | South Africa | 2003 | 1256 | Adolescents | Multistage stratified sampling | - | 43.70% | Schools | Urban | Quantitative | Cross-sectional | Self-administered questionnaire | No | Yes | No |
| 107 | Fatoye FO. | Psychosocial correlates of substance use amongst secondary school students in south western Nigeria. | Nigeria | 2003 | 562 | School going adolescents | - | - | 47.32% | Schools | Urban | Quantitative | Cross-sectional | Self-administered questionnaire | No | No | No |
| 108 | Swart D, Reddy P, Ruiter RA, de Vries H. | Cigarette use among male and female grade 8-10 students of different ethnicity in South African schools. | South Africa | 2003 | 6045 | Adolescents | Multistage cluster sampling | - | 47.40% | Schools | National | Quantitative | Cross-sectional | Self-administered questionnaire | No | Yes | Yes |
| 109 | Ibeh CC, Ele PU. | Prevalence of cigarette smoking in young Nigerian females. | Nigeria | 2003 | 1113 | School going adolescents and youths aged 12 to 20 years | Multistage cluster sampling | 16.06 | 0% | Schools | - | Quantitative | Cross-sectional | Self-administered questionnaire | No | No | No |
| 110 | Madu SN, Matla MQ. | Illicit drug use, cigarette smoking and alcohol drinking behaviour among a sample of high school adolescents in the Pietersburg area of the Northern Province, South Africa. | South Africa | 2003 | 435 | School going adolescents aged 15 to 19 years | Multistage stratified cluster sampling | 17.25 | 44% | Schools | Both | Quantitative | Cross-sectional | Self-administered questionnaire | No | No | No |
| 111 | Peltzer K. | Smokeless tobacco and cigarette use among black secondary school students in South Africa. | South Africa | 2003 | 712 | School going adolescents and young adults aged 13 to 23 years | Multistage stratified sampling | - | 46.07% | Schools | Rural | Quantitative | Cross-sectional | Self-administered questionnaire | No | No | No |
| 112 | Gilbert AR, Pinget C, Bovet P, Cornuz J, Shamlaye C, Paccaud F. | The cost effectiveness of pharmacological smoking cessation therapies in developing countries: a case study in the Seychelles. | Seychelles | 2004 | - | - | - | - | - | - | - | Quantitative | Interventional study | - | No | No | No |
| 113 | Sitas F, Urban M, Bradshaw D, Kielkowski D, Bah S, Peto R. | Tobacco attributable deaths in South Africa. | South Africa | 2004 | 5340 | Reported adult deaths above the age of 25 years | Purposive sampling | - | - | - | National | Quantitative | Case control | Administrative data/document review | No | Yes | No |
| 114 | Ayo-Yusuf OA, Swart TJ, Pickworth WB. | Nicotine delivery capabilities of smokeless tobacco products and implications for control of tobacco dependence in South Africa. | South Africa | 2004 | - | Smokeless tobacco | Purposive sampling | - | - | Community/household | - | Quantitative | Cross-sectional | Observation | No | No | No |
| 115 | Maassen IT, Kremers SP, Mudde AN, Joof BM. | Smoking initiation among Gambian adolescents: social cognitive influences and the effect of cigarette sampling. | Gambia | 2004 | 282 | Adolescents aged 14 to 18 years | Cluster sampling | 16 | 63.80% | Schools | National | Quantitative | Cross-sectional | Self-administered questionnaire | No | No | No |
| 116 | Mpabulungi L, Muula AS. | Tobacco use among high shool students in Kampala, Uganda: questionnaire study. | Uganda | 2004 | 2789 | Adolescents | Multistage cluster sampling | - | - | Schools | Urban | Quantitative | Cross-sectional | Self-administered questionnaire | No | Yes | Yes |
| 117 | Astrøm AN, Ogwell EA. | Use of tobacco in Kenya: sources of information, beliefs and attitudes toward tobacco control measures among primary school students. | Kenya | 2004 | 1130 | Adolescents | Multistage random cluster sampling | 14.1 | 52% | Schools | Urban | Quantitative | Cross-sectional | Self-administered questionnaire | No | No | No |
| 118 | Nollen NL, Adewale S, Okuyemi KS, Ahluwalia JS, Parakoyi A. | Workplace tobacco policies and smoking cessation practices of physicians. | Nigeria | 2004 | 373 | Adults | Convenience sampling | 33 | 84% | Workplace | Urban | Quantitative | Cross-sectional | Self-administered questionnaire | No | No | No |
| 119 | Everett K, Odendaal HJ, Steyn K. | Doctors' attitudes and practices regarding smoking cessation during pregnancy. | South Africa | 2005 | 15 | Adults | Purposive sampling | - |  | Workplace | Urban | Qualitative | Thematic analysis | Interview | No | No | No |
| 120 | Lawoyin TO, Ajumobi OO, Abdul MM, Abdul Malik JO, Adegoke DA, Agbedeyi OA. | Drug use among senior secondary school students in rural Nigeria. | Nigeria | 2005 | 394 | Adolescents and youths aged 14 to 24 years | Multistage cluster sampling | - | 55.08% | Schools | Rural | Quantitative | Cross-sectional | Interview | No | No | No |
| 121 | Pampel FC. | Patterns of tobacco use in the early epidemic stages: Malawi and Zambia, 2000-2002. | Malawi and Zambia | 2005 | 9744 in Zambia and 16176 in Malawi | Women aged 15-49 years and men aged 15 to 59 years | Multistage stratified cluster sampling | - | 78.14% in Zambia and 81.57% in Malawi | - | National | Quantitative | Cross-sectional | Interview | No | No | No |
| 122 | Panday S, Reddy SP, Ruiter RA, Bergström E, de Vries H. | Determinants of smoking cessation among adolescents in South Africa. | South Africa | 2005 | 1267 | Adolescents aged 14 to 16 years | Multistage stratified sampling | 16.06 | 51% | Schools | Urban | Quantitative | Cross-sectional | Self-administered questionnaire | No | Yes | No |
| 123 | Kaduri P, Gilreath T, King G, Mbwambo J, Kilonzo G, Flisher AJ, Matthews SA. | Social networks' influence on tobacco use among students in Dar es Salaam, Tanzania. | Tanzania | 2005 | 3816 | Students aged 10 to 20 years | Multistage stratified sampling | 14.7 | 50.40% | Schools | Both | Quantitative | Cross-sectional | Self-administered questionnaire | No | No | No |
| 124 | Swart D, Panday S, Reddy SP, Bergström E, de Vries H. | Access point analysis: what do adolescents in South Africa say about tobacco control programmes? | South Africa | 2006 | 117 | Adolescents | Purposive sampling | - | 48.71% | Schools | Both | Qualitative | Thematic analysis | Focus group discussion | No | No | No |
| 125 | Ayo-Yusuf O, Peltzer K, Mufamadi J. | Traditional healers' perceptions of smokeless tobacco use and health in the Limpopo Province of South Africa. | South Africa | 2006 | 28 | Adults aged 35 to 83 years | Random sampling | 55 | 32.10% | Workplace | Both | Qualitative | Thematic analysis | Interview | No | No | No |
| 126 | Morojele NK, Brook JS. | Substance use and multiple victimisation among adolescents in South Africa. | South Africa | 2006 | 1474 | Adolescents aged 12 to 17 years | Multistage stratified sampling | 14.68 | 45.50% | Community/household | Urban | Quantitative | Cross-sectional | Interview | No | Yes | No |
| 127 | Steyn K, de Wet T, Saloojee Y, Nel H, Yach D. | The influence of maternal cigarette smoking, snuff use and passive smoking on pregnancy outcomes: the Birth To Ten Study. | South Africa | 2006 | 1593 | Adults | Purposive sampling (all the pregnant women visiting services for antenatal or birth were enrolled) | 25.6 | 0% | Health facility | Urban | Quantitative | Cross-sectional | Interview | No | No | No |
| 128 | Ugheoke AJ, Ebomoyi MI, Iyawe VI. | Influence of smoking on respiratory symptoms and lung function indices in sawmill workers in Benin City, Nigeria. | Nigeria | 2006 | 300 | Adult factory workers aged 18 to 50 years | Multistage stratified sampling | - | - | Workplace | Urban | Quantitative | Cross-sectional | Interview | No | No | No |
| 129 | Mpabulungi L, Muula AS. | Tobacco use among high school students in a remote district of Arua, Uganda. | Uganda | 2006 | 1528 | Adolescents | Multistage cluster sampling | - | 65.80% | Schools | Rural | Quantitative | Cross-sectional | Self-administered questionnaire | No | Yes | Yes |
| 130 | Faeh D, Viswanathan B, Chiolero A, Warren W, Bovet P. | Clustering of smoking, alcohol drinking and cannabis use in adolescents in a rapidly developing country. | Seychelles | 2006 | 1442 | School going adolescents aged 11 to 17 years | Multistage cluster sampling | - | 48.67% | Schools | National | Quantitative | Cross-sectional | Self-administered questionnaire | No | No | Yes |
| 131 | Bovet P, Viswanathan B, Faeh D, Warren W. | Comparison of smoking, drinking, and marijuana use between students present or absent on the day of a school-based survey. | Seychelles | 2006 | 1426 | School going adolescents aged 11 to 17 years | Multistage cluster sampling | - | 49.89% | Schools | National | Quantitative | Cross-sectional | Self-administered questionnaire | No | No | Yes |
| 132 | Taiwo T, Goldstein S. | Drug use and its association with deviant behaviour among rural adolescent students in South Africa. | South Africa | 2006 | 357 | School going adolescents aged 11 to 19 years | Multistage cluster sampling | 15.8 | 45.90% | Schools | Rural | Quantitative | Cross-sectional | Self-administered questionnaire | No | Yes | No |
| 133 | Amoateng AY, Barber BK, Erickson LD. | Family predictors of adolescent substance use: the case of high school students in the Cape Metropolitan Area, Cape Town, South Africa. | South Africa | 2006 | 1800 | School going adolescents | Purposive sampling | 15.95 | - | Schools | Urban | Quantitative | Cross-sectional | Self-administered questionnaire | No | No | No |
| 134 | Brook JS, Morojele NK, Brook DW, Zhang C, Whiteman M. | Personal, interpersonal, and cultural predictors of stages of cigarette smoking among adolescents in Johannesburg, South Africa. | South Africa | 2006 | 731 | Adolescents aged 12 to 17 years | Stratified random sampling | 14.5 | 47% | Community/household | Urban | Quantitative | Cross-sectional | Interview | No | Yes | No |
| 135 | Fernander AF, Flisher AJ, King G, Noubary F, Lombard C, Price M, Chalton D. | Gender differences in depression and smoking among youth in Cape Town, South Africa. | South Africa | 2006 | 623 | Adolescents | Multistage stratified random sampling | - | 42% | Schools | Urban | Quantitative | Cross-sectional | Self-administered questionnaire | No | No | No |
| 136 | Otañez MG, Mamudu H, Glantz SA. | Global leaf companies control the tobacco market in Malawi. | Malawi | 2007 | 124 government officials and 45 documents | Government officials and documents | Purposive sampling | - | - | Workplace | - | Qualitative | Case study | Interview and Administrative data/documents | No | No | No |
| 137 | Jackson DJ, Batiste E, Rendall-Mkosi K. | Effect of smoking and alcohol use during pregnancy on the occurrence of low birthweight in a farming region in South Africa. | South Africa | 2007 | 400 | Newborns | Purposive sampling | - | - | Health facility | - | Quantitative | Case control | Administrative data/document review and interview | Yes | No | No |
| 138 | Mufunda J, Debesay A, Mosazghi A, Nyarango P, Usman A, Mebrahtu G, Kosia A, Equbamichael M, Yohannes E, Ghebrat Y, Paulos E, Rizzo S, Masjuan M, Gebremichael A. | Prevalence of tobacco use in Eritrea: results from a noncommunicable disease risk factor survey. | Eritrea | 2007 | 2304 | Adolescents and adults aged 15 to 64 years | Multistage cluster sampling | - | 48.74% | Community/household | National | Quantitative | Cross-sectional | Interview | No | No | No |
| 139 | Gureje O, Degenhardt L, Olley B, Uwakwe R, Udofia O, Wakil A, Adeyemi O, Bohnert KM, Anthony JC. | A descriptive epidemiology of substance use and substance use disorders in Nigeria during the early 21st century. | Nigeria | 2007 | 6752 | Adults aged 18 years and more | Multistage stratified sampling | - | 48.98% | Community/household | Both | Quantitative | Cross-sectional | Interview | No | Yes | No |
| 140 | Tanimowo MO, Onaolapo YA. | The pattern of tobacco use among non-pulmonary tuberculosis patients attending a chest clinic in south-western Nigeria. | Nigeria | 2007 | 104 | Adult patients | Random sampling | - | - | Health facility | Urban | Quantitative | Cross-sectional | Interview | No | No | No |
| 141 | van Walbeek C, Blecher E, van Graan M. | Effects of the Tobacco Products Control Amendment Act of 1999 on restaurant revenues in South Africa--a survey approach. | South Africa | 2007 | 1101 | Adults | Convenience sampling | - | 65% | Recreational facility | Both | Quantitative | Cross-sectional | Interview | No | No | No |
| 142 | Ureme SO, Ibeagha ID, Maduka IG, Ibeagbulam OG. | The concentrations of methaemoglogin, carboxyhaemoglobin and some haematological parameters in tobacco snuff addicts in Igbo of Nigeria. | Nigeria | 2007 | 100 | Adults aged 25 to 65 years | Purposive sampling | - | 65% | Community/household | Rural | Quantitative | Cross-sectional | Observation | No | Yes | No |
| 143 | den Boon S, Verver S, Marais BJ, Enarson DA, Lombard CJ, Bateman ED, Irusen E, Jithoo A, Gie RP, Borgdorff MW, Beyers N. | Association between passive smoking and infection with Mycobacterium tuberculosis in children. | South Africa | 2007 | 1344 | Children | Random sampling | | 50.52% | Community/household | Urban | Quantitative | Cross-sectional | Observation | No | Yes | No |
| 144 | Ahmed HG, Mahgoob RM. | Impact of Toombak dipping in the etiology of oral cancer: gender-exclusive hazard in the Sudan. | Sudan | 2007 | 82 | Adult patients aged 18 to 76 years | Purposive sampling | 48 | - | Health facility | - | Quantitative | Cross-sectional | Observation | No | No | No |
| 145 | Munyati SS, Redzo N, Dauya E, Matambo R, Makamure B, Bandason T, Butterworth AE, Gwanzura L, Rusakaniko S, Mason PR, Corbett EL. | Human immunodeficiency virus, smoking and self-rated health in Harare, Zimbabwe. | Zimbabwe | 2007 | 6111 | Adults | Convenience sampling | - | - | Workplace | Urban | Quantitative | Cross-sectional | Observation | No | No | No |
| 146 | Nwhator SO, Winfunke-Savage K, Ayanbadejo P, Jeboda SO. | Smokers' melanosis in a Nigerian population: a preliminary study. | Nigeria | 2007 | 253 | Adult factory workers | Purposive sampling | - | - | Workplace | Both | Quantitative | Cross-sectional | Observation | No | Yes | No |
| 147 | Unverdorben M, der Bijl A, Potgieter L, Liang Q, Meyer BH, Roethig HJ. | Effects of levels of cigarette smoke exposure on symptom-limited spiroergometry. | South Africa | 2007 | 18 | Adults | Convenience sampling | - | - | - | - | Quantitative | Randomized controlled trial crossover study | Observation | No | No | No |
| 148 | Ayo-Yusuf OA, Reddy PS, van Wyk PJ, van den Borne BW. | Household smoking as a risk indicator for caries in adolescents' permanent teeth. | South Africa | 2007 | 1873 | Adolescents | Multistage cluster sampling | 14.6 | 49.33% | Schools | National | Quantitative | Cross-sectional | Observation | No | No | No |
| 149 | Cheyip MY, Nelson G, Ross MH, Murray J. | South African platinum mine employees reduce smoking in 5 years. | South Africa | 2007 | 25274 | Adults | Convenience sampling | 40 | 97.40% | Workplace | National | Quantitative | Cross-sectional (data collected in multiple time points) | Observation | No | No | No |
| 150 | Reddy P, Resnicow K, Omardien R, Kambaran N. | Prevalence and correlates of substance use among high school students in South Africa and the United States. | South Africa | 2007 | 7773 (in South Africa) | Adolescents | Multistage cluster sampling | - | - | Schools | National | Quantitative | Cross-sectional | Self-administered questionnaire | No | No | No |
| 151 | Panday S, Reddy SP, Ruiter RA, Bergström E, de Vries H. | Determinants of smoking among adolescents in the Southern Cape-Karoo region, South Africa. | South Africa | 2007 | 3378 | Adolescents | Multistage cluster sampling | 16.23 | 58.20% | Schools | Both | Quantitative | Cross-sectional | Self-administered questionnaire | No | Yes | No |
| 152 | Muula AS, Mpabulungi L. | Cigarette smoking prevalence among school-going adolescents in two African capital cities: Kampala Uganda and Lilongwe Malawi. | Uganda and Malawi | 2007 | 1820 in Malawi and 2789 in Uganda | Adolescents aged 13 - 17 years | Multistage cluster sampling | 14 | 47.8% in Malawi and 46.4% in Uganda | Schools | Urban | Quantitative | Cross-sectional | Self-administered questionnaire | No | No | Yes |
| 153 | Rudatsikira E, Abdo A, Muula AS. | Prevalence and determinants of adolescent tobacco smoking in Addis Ababa, Ethiopia. | Ethiopia | 2007 | 1868 | Adolescents aged 13 to 15 years | Multistage cluster sampling | 15 | 43.70% | Schools | Urban | Quantitative | Cross-sectional | Self-administered questionnaire | No | Yes | Yes |
| 154 | Muula AS, Siziya S. | Prevalence and determinants of ever smoked cigarettes among school-going adolescents in Lusaka, Zambia. | Zambia | 2007 | 2175 | Adolescents aged 13 to 15 years | Multistage cluster sampling | - | - | Schools | Urban | Quantitative | Cross-sectional | Self-administered questionnaire | No | Yes | Yes |
| 155 | Panday S, Reddy SP, Ruiter RA, Bergström E, de Vries H. | Nicotine dependence and withdrawal symptoms among occasional smokers. | South Africa | 2007 | 554 | Adolescents | Multistage stratified sampling | 16.3 | 59.40% | Schools | Both | Quantitative | Cross-sectional | Self-administered questionnaire | No | Yes | No |
| 156 | Siziya S, Rudatsikira E, Muula AS, Ntata PR. | Predictors of cigarette smoking among adolescents in rural Zambia: results from a cross sectional study from Chongwe [corrected] district. | Zambia | 2007 | 1882 | Adolescents | Multistage cluster sampling | - | - | Schools | Rural | Quantitative | Cross-sectional | Self-administered questionnaire | No | Yes | Yes |
| 157 | Omokhodion FO, Faseru BO. | Perception of cigarette smoking and advertisement among senior secondary school students in Ibadan, Southwestern Nigeria. | Nigeria | 2007 | 1223 | School going adolescents | Multistage cluster sampling | - | 53% | Schools | Urban | Quantitative | Cross-sectional | Self-administered questionnaire | No | No | No |
| 158 | Siziya S, Ntata PR, Rudatsikira E, Makupe CM, Umar E, Muula AS. | Sex differences in prevalence rates and predictors of cigarette smoking among in-school adolescents in Kilimanjaro, Tanzania. | Tanzania | 2007 | 2323 | School going adolescents | Multistage cluster sampling | - | 47% | Schools | - | Quantitative | Cross-sectional | Self-administered questionnaire | No | No | Yes |
| 159 | Siziya S, Rudatsikira E, Muula AS. | Cigarette smoking among school-going adolescents in Kafue, Zambia. | Zambia | 2007 | 1872 | School going adolescents | Multistage cluster sampling | - | 47.60% | Schools | - | Quantitative | Cross-sectional | Self-administered questionnaire | No | Yes | Yes |
| 160 | Makanjuola AB, Daramola TO, Obembe AO. | Psychoactive substance use among medical students in a Nigerian university. | Nigeria | 2007 | 906 | Adult medical students | Purposive sampling | 22.4 | 69% | Schools | - | Quantitative | Cross-sectional | Self-administered questionnaire | No | No | No |
| 161 | El Sony A, Slama K, Salieh M, Elhaj H, Adam K, Hassan A, Enarson DA. | Feasibility of brief tobacco cessation advice for tuberculosis patients: a study from Sudan. | Sudan | 2007 | 48 | Adults | Convenience sampling | - | - | Health facility | Both | Quantitative | Quasi-experimental | Self-administered questionnaire and interview | No | Yes | No |
| 162 | Groenewald P, Vos T, Norman R, Laubscher R, van Walbeek C, Saloojee Y, Sitas F, Bradshaw D; South African Comparative Risk Assessment Collaborating Group.. | Estimating the burden of disease attributable to smoking in South Africa in 2000. | South Africa | 2007 | - | Individuals aged 15 years and more | Multistage cluster sampling | - | 43.70% | Community/household | National | Quantitative | Cross-sectional | Self-administered questionnaire | No | No | No |
| 163 | Stein L, Urban MI, Weber M, Ruff P, Hale M, Donde B, Patel M, Sitas F. | Effects of tobacco smoking on cancer and cardiovascular disease in urban black South Africans. | South Africa | 2008 | 9690 | Adults | - | 52 | 35% | Health facility | Urban | Quantitative | Case control | Interview | No | No | No |
| 164 | Ramin B, Kam D, Feleke B, Jacob B, Jha P. | Smoking, HIV and non-fatal tuberculosis in an urban African population. | Ethiopia | 2008 | 153 | Adults aged 18 to 65 years | Purposive sampling | - | 100% | Health facility | Urban | Quantitative | Case control | Interview | No | Yes | No |
| 165 | Pampel F. | Tobacco use in sub-Sahara Africa: estimates from the demographic health surveys. | Namibia, Malawi, Uganda, Zambia, Ghana, Kenya, Mozambique, Nigeria, Madagascar, Tanzania, Lesotho, Ethiopia, Rwanda, Zimbabwe | 2008 | Namibia: 9618, Malawi: 31221, Uganda: 20236, Zambia: 9727, Ghana: 10505, Kenya: 11766, Mozambique: 15077, Nigeria: 9831, Madagascar: 10295, Tanzania: 12960, Lesotho: 9749, Ethiopia: 12901, Rwanda: 15981, Zimbabwe: 16067 | Men aged 15 to 54 years and women aged 15 to 49 years | Multistage stratified cluster sampling | - | - | Community/household | National | Quantitative | Cross-sectional | Interview | No | Yes | No |
| 166 | Williams CT, Grier SA, Marks AS. | "Coming to town": the impact of urbanicity, cigarette advertising, and network norms on the smoking attitudes of black women in Cape Town, South Africa. | South Africa | 2008 | 975 | Individuals aged 16 to 65 years | Multistage stratified sampling | - | 0% | Community/household | Urban | Quantitative | Cross-sectional | Interview | No | No | No |
| 167 | Bloch M, Althabe F, Onyamboko M, Kaseba-Sata C, Castilla EE, Freire S, Garces AL, Parida S, Goudar SS, Kadir MM, Goco N, Thornberry J, Daniels M, Bartz J, Hartwell T, Moss N, Goldenberg R. | Tobacco use and secondhand smoke exposure during pregnancy: an investigative survey of women in 9 developing nations | Democratic Republic of Congo and Zambia | 2008 | 1756 | Adults aged 18 to 46 years | Convenience sampling | Congo: 27.32 and Zambia: 24.99 | 0% | Health facility | Both | Quantitative | Cross-sectional | Interview | No | Yes | No |
| 168 | Ward CL, Mertens JR, Flisher AJ, Bresick GF, Sterling SA, Little F, Weisner CM. | Prevalence and correlates of substance use among South African primary care clinic patients. | South Africa | 2008 | 2618 | Adults | Multistage cluster stratified sampling | - | 43.10% | Health facility | Urban | Quantitative | Cross-sectional | Interview | No | No | No |
| 169 | Aina OF, Olorunshola DA. | Alcohol and substance use portrayals in Nigerian video tapes: an analysis of 479 films and implications for public drug education. | Nigeria | 2008 | 479 | Movies | Random sampling | - | - | Community/household | Urban | Quantitative | Cross-sectional | Observation | No | No | No |
| 170 | Simen-Kapeu A, La Ruche G, Kataja V, Yliskoski M, Bergeron C, Horo A, Syrjänen K, Saarikoski S, Lehtinen M, Dabis F, Sasco AJ. | Tobacco smoking and chewing as risk factors for multiple human papillomavirus infections and cervical squamous intraepithelial lesions in two countries (Côte d'Ivoire and Finland) with different tobacco exposure | Cote d'Ivoire | 2008 | 2198 | Adults aged 20 to 50 years | - | - | 0% | Health facility | Urban | Quantitative | Cross-sectional | Observation | No | Yes | No |
| 171 | Ayo-Yusuf OA, Reddy PS, van den Borne BW. | Association of snuff use with chronic bronchitis among South African women: implications for tobacco harm reduction. | South Africa | 2008 | 4464 | Adults aged 25 years or older | - | - | 0% | Community/household | National | Quantitative | Cross-sectional | Observation | No | No | No |
| 172 | Ayo-Yusuf OA, Reddy PS, van den Borne BW. | Adolescents' sense of coherence and smoking as longitudinal predictors of self-reported gingivitis. | South Africa | 2008 | 970 | School going adolescents | Multistage cluster sampling | 14.4 | - | Schools | Rural | Quantitative | Cohort | Observation | No | Yes | No |
| 173 | Viswanathan B, Warren CW, Jones NR, Asma S, Bovet P. | Linking Global Youth Tobacco Survey (GYTS) data to the WHO Framework Convention on Tobacco Control (FCTC): the case for the Seychelles. | Seychelles | 2008 | 1321 | School going adolescents aged 13 to 15 years | Multistage cluster sampling | - | - | Schools | National | Quantitative | Cross-sectional | Self-administered questionnaire | No | Yes | Yes |
| 174 | Siziya S, Rudatsikira E, Muula AS. | Antismoking messages and current cigarette smoking status in Somaliland: results from the Global Youth Tobacco Survey 2004. | Somaliland | 2008 | 1122 | School going adolescents aged 13 to 15 years | Multistage cluster sampling | - | 63.30% | Schools | National | Quantitative | Cross-sectional | Self-administered questionnaire | No | No | Yes |
| 175 | Kaduri P, Kitua H, Mbatia J, Kitua AY, Mbwambo J. | Smokeless tobacco use among adolescents in Ilala Municipality, Tanzania. | Tanzania | 2008 | 1010 | School going adolescents | Random sampling | - | 50.70% | Schools | Urban | Quantitative | Cross-sectional | Self-administered questionnaire | No | No | No |
| 176 | Abasiubong F, Atting I, Bassey E, Ekott J. | A comparative study of use of psychoactive substances amongst secondary school students in two local Government Areas of Akwa Ibom State, Nigeria. | Nigeria | 2008 | 254 | School going adolescents and adults aged 11 to 30 years | Random sampling | - | 62.99% | Schools | Urban | Quantitative | Cross-sectional | Self-administered questionnaire | No | No | No |
| 177 | Smith EA, Palen LA, Caldwell LL, Flisher AJ, Graham JW, Mathews C, Wegner L, Vergnani T. | Substance use and sexual risk prevention in Cape Town, South Africa: an evaluation of the HealthWise program. | South Africa | 2008 | 2176 | School going adolescents | Random sampling | 14 | 49% | Schools | Urban | Quantitative | Randomized control trial | Self-administered questionnaire | No | No | No |
| 178 | Resnicow K, Reddy SP, James S, Gabebodeen Omardien R, Kambaran NS, Langner HG, Vaughan RD, Cross D, Hamilton G, Nichols T. | Comparison of two school-based smoking prevention programs among South African high school students: results of a randomized trial. | South Africa | 2008 | 5266 | Adolescents | Multistage random cluster sampling | 14.7 | 58.10% | Schools | Both | Quantitative | Randomized controlled trial | Self-administered questionnaire | No | No | No |
| 179 | Owusu-Dabo E, Lewis S, McNeill A, Anderson S, Gilmore A, Britton J. | Smoking in Ghana: a review of tobacco industry activity. | Ghana | 2009 | 300 | Documents | Purposive sampling | - | - | - | National | Qualitative | Case study | Administrative data/document review | No | Yes | No |
| 180 | Patel P, Okechukwu CA, Collin J, Hughes B. | Bringing 'Light, Life and Happiness': British American Tobacco and musicsponsorship in sub-Saharan Africa. | Sub-saharan Africa | 2009 | 126 | - | - | - | - | - | - | Qualitative | Case study | Administrative data/document review | No | Yes | No |
| 181 | Petersen Z, Nilsson M, Everett K, Emmelin M. | Possibilities for transparency and trust in the communication between midwives and pregnant women: the case of smoking. | South Africa | 2009 | 12 | Adults | Purposive sampling | - | 0% | Health facility | Urban | Qualitative | Grounded theory | Interview | No | Yes | No |
| 182 | Otañez MG, Mamudu HM, Glantz SA. | Tobacco companies' use of developing countries' economic reliance on tobacco to lobby against global tobacco control: the case of Malawi. | Malawi | 2009 | 124 | Adults and industrial documents | Purposive sampling | - | - | Workplace | National | Qualitative | Case study | Interview and Administrative data/documents | No | Yes | No |
| 183 | Okeh UM. | Statistical measure of association between smoking and lung cancer in Abakaliki, Ebonyi State Nigeria. | Nigeria | 2009 | 1673 | Adults | - | - | - | Community/household | Urban | Quantitative | Cross-sectional | - | No | No | No |
| 184 | Patrick ME, Collins LM, Smith E, Caldwell L, Flisher A, Wegner L. | A prospective longitudinal model of substance use onset among South African adolescents. | South Africa | 2009 | 1118 | School going adolescents | Random sampling | - | 49.10% | Schools | Urban | Quantitative | Cohort | - | No | No | No |
| 185 | Bissessur S, Naidoo S. | Areca nut and tobacco chewing habits in Durban, KwaZulu Natal. | South Africa | 2009 | 101 | Adults | Convenience sampling | 35 | 44.55% | Community/household | Urban | Quantitative | Cross-sectional | Interview | No | Yes | No |
| 186 | Owusu-Dabo E, Lewis S, McNeill A, Gilmore A, Britton J. | Smoking uptake and prevalence in Ghana | Ghana | 2009 | 6258 | Individuals aged 14 years and more | Multistage cluster sampling | - | 36.30% | Community/household | Both | Quantitative | Cross-sectional | Interview | No | Yes | No |
| 187 | van Heerden MS, Grimsrud AT, Seedat S, Myer L, Williams DR, Stein DJ. | Patterns of substance use in South Africa: results from the South African Stress and Health study. | South Africa | 2009 | 4351 | Adults aged 18 years or more | Multistage stratified sampling | - | 39.80% | Community/household | National | Quantitative | Cross-sectional | Interview | No | No | No |
| 188 | Brook JS, Pahl K, Morojele NK. | The Relationship Between Receptivity to Media Models of Smoking and Nicotine Dependence Among South African Adolescents. | South Africa | 2009 | 731 | Adolescents aged 12 to 17 years | Stratified random sampling | 14.55 | 47% | Community/household | Urban | Quantitative | Cross-sectional | Interview | No | Yes | No |
| 189 | Mbatia J, Jenkins R, Singleton N, White B. | Prevalence of alcohol consumption and hazardous drinking, tobacco and drug use in urban Tanzania, and their associated risk factors. | Tanzania | 2009 | 899 | Adolescents and adults aged 15 to 59 years | Systematic random sampling | - | 44% | Community/household | Urban | Quantitative | Cross-sectional | Interview | No | No | No |
| 190 | Ndetei DM, Khasakhala LI, Ongecha-Owuor FA, Kuria MW, Mutiso V, Kokonya DA. | Prevalence of substance abuse among patients in general medical facilities in Kenya. | Kenya | 2009 | 2770 | Adults | Systematic sampling | - | 42.80% | Health facility | Both | Quantitative | Cross-sectional | Interview | No | Yes | No |
| 191 | Amdzaranda PA, Fatoye FO, Oyebanji AO, Ogunro AS, Fatoye GK. | Factors associated with psychoactive substance use among a sample of prison inmates in Ilesa, Nigeria. | Nigeria | 2009 | 303 | Adolescents and adults aged 15 to 70 years | - | 31.2 | 96.40% | Prison | - | Quantitative | Cross-sectional | Interview | No | No | No |
| 192 | Addo J, Smeeth L, Leon DA. | Smoking patterns in Ghanaian civil servants: changes over three decades. | Ghana | 2009 | 1015 | Adults aged 25 years and more | Random sampling | - | - | Workplace | Urban | Quantitative | Cross-sectional | Interview | No | Yes | No |
| 193 | Peer N, Bradshaw D, Laubscher R, Steyn K. | Trends in adult tobacco use from two South African Demographic and Health Surveys conducted in 1998 and 2003. | South Africa | 2009 | 13286 in first DHS and 8115 in second DHS | Adolescents and adults aged 15 and more | Multistage cluster sampling | - | 41-42% | Community/household | National | Quantitative | Cross-sectional (data collected in multiple time points) | Interview | No | Yes | No |
| 194 | Iwegbue CM, Nwajei GE, Eguavoen O. | Metal distribution in some brands of cigarette ash in Nigeria. | Nigeria | 2009 | 15 | Cigarettes | Purposive sampling | - | - | Community/household | - | Quantitative | Cross-sectional | Observation | No | No | No |
| 195 | Nwhator SO, Olagundoye O. | Do smokers benefit from dental hygiene oral prophylaxis? A Nigerian pilot study. | Nigeria | 2009 | 21 | Adults | Convenience sampling | - | - | Workplace | - | Quantitative | Non-randomized interventional study | Observation | No | No | No |
| 196 | Jaquet A, Ekouevi DK, Aboubakrine M, Bashi J, Messou E, Maiga M, Traore HA, Zannou M, Guehi C, Ba-Gomis FO, Minga A, Allou G, Eholie SP, Dabis F, Bissagnene E, Sasco AJ. | Tobacco use and its determinants in HIV-infected patients on antiretroviral therapy in West African countries | Cote d'Ivoire, Benin and Mali | 2009 | 2920 | Adults | Systematic sampling | 38 | 29.00% | Health facility | Urban | Quantitative | Cross-sectional | Self-administered questionnaire | No | Yes | No |
| 197 | Peltzer K. | Prevalence and correlates of substance use among school children in six African countries. | Kenya, Namibia, Swaziland, Uganda, Zambia, Zimbabwe | 2009 | 20765 | Adolescents | Multistage cluster random sampling | - | 47.70% | Schools | National | Quantitative | Cross-sectional | Self-administered questionnaire | No | Yes | No |
| 198 | Amemori M, Mumghamba EG, Ruotoistenmäki J, Murtomaa H. | Smoking and drinking habits and attitudes to smoking cessation counselling among Tanzanian dental students. | Tanzania | 2009 | 109 | Adult dental students | Convenience sampling | - | 76.10% | Schools | Urban | Quantitative | Cross-sectional | Self-administered questionnaire | No | No | No |
| 199 | Rudatsikira E, Muula AS, Siziya S. | Exposure to environmental tobacco smoke among adolescents in Kampala-Uganda, 2002. | Uganda | 2009 | 2427 | School going adolescents aged 13 to 15 years | Multistage cluster sampling | - | 47.20% | Schools | National | Quantitative | Cross-sectional | Self-administered questionnaire | No | Yes | Yes |
| 200 | Zulu R, Siziya S, Muula AS, Rudatsikira E. | Associations of advertisement-promotion-sponsorship-related factors with current cigarette smoking among in-school adolescents in Zambia. | Zambia | 2009 | 2378 | Adolescents | Multistage cluster sampling | - | 49.20% | Schools | National | Quantitative | Cross-sectional | Self-administered questionnaire | No | No | Yes |
| 201 | Ndetei DM, Khasakhala LI, Mutiso V, Ongecha-Owuor FA, Kokonya DA. | Patterns of drug abuse in public secondary schools in Kenya. | Kenya | 2009 | 1296 | Adolescents and young adults aged 13 to 24 years | Multistage stratified sampling | 17 | 62.50% | Schools | Urban | Quantitative | Cross-sectional | Self-administered questionnaire | No | No | No |
| 202 | Odeyemi KA, Osibogun A, Akinsete AO, Sadiq L. | The Prevalence and Predictors of Cigarette Smoking among Secondary School Students in Nigeria. | Nigeria | 2009 | 1183 | School going adolescents | Multistage stratified sampling | - | - | Schools | Both | Quantitative | Cross-sectional | Self-administered questionnaire | No | No | No |
| 203 | Aina BA, Oyerinde OO, Joda AE, Dada OO. | Cigarette smoking among healthcare professional students of University of Lagos and Lagos University Teaching Hospital (LUTH), Idi-Araba, Lagos, Nigeria. | Nigeria | 2009 | 433 | Students aged 16 and more | Purposive sampling | - | 46.88% | Schools | Urban | Quantitative | Cross-sectional | Self-administered questionnaire | No | No | No |
| 204 | Aina BA, Onajole AT, Lawal BM, Oyerinde OO. | Promoting cessation and a tobacco free future: willingness of pharmacy students at the University of Lagos, Nigeria. | Nigeria | 2009 | 291 | Students aged 16 to 30 years | Purposive sampling | - | 37.50% | Schools | Urban | Quantitative | Cross-sectional | Self-administered questionnaire | No | No | No |
| 205 | Desalu OO, Adekoya AO, Elegbede AO, Dosunmu A, Kolawole TF, Nwogu KC. | Knowledge of and practices related to smoking cessation among physicians in Nigeria. | Nigeria | 2009 | 436 | Adults | Multistage stratified clustered random sampling | 30.6 | 75.70% | Workplace | Both | Quantitative | Cross-sectional | Self-administered questionnaire | No | No | No |
| 206 | Ayo-Yusuf OA, van den Borne B, Reddy PS, van Wyk PJ, Severson HH. | Longitudinal association of smoking-related attitude to oral health with adolescents' smoking onset. | South Africa | 2009 | 422 | School going adolescents | Random sampling | 13.9 | 46.10% | Schools | Urban | Quantitative | Cohort | Self-administered questionnaire | No | No | No |
| 207 | Owusu-Dabo E, McNeill A, Lewis S, Gilmore A, Britton J. | Status of implementation of Framework Convention on Tobacco Control (FCTC) in Ghana: a qualitative study | Ghana | 2010 | 20 | Adults (Members of National Steering Committee for tobacco control in Ghana) | Purposive sampling | - | - | Workplace | National | Qualitative | Case study | Interview | No | No | No |
| 208 | Girma E, Assefa T, Deribew A. | Cigarette smokers' intention to quit smoking in Dire Dawa town Ethiopia: an assessment using the Transtheoretical Model | Ethiopia | 2010 | 384 | Adults | Multistage random sampling | 30.95 | 92.40% | Community/household | Both | Qualitative | Grounded theory | Interview | No | No | No |
| 209 | Petersen Z, Steyn K, Everett-Murphy K, Emmelin M. | Pregnant women's responses to a tailored smoking cessation intervention: turning hopelessness into competence. | South Africa | 2010 | 13 | Individuals aged 16 to 40 years | Purposive sampling | - | 0% | Health facility | Urban | Qualitative | Thematic analysis | Interview | No | No | No |
| 210 | Nakkash R, Khalil J. | Health warning labelling practices on narghile (shisha, hookah) waterpipe tobacco products and related accessories. | South Africa | 2010 | 2 | Waterpipe tobacco | Convenience sampling | - | - | Community/household | - | Qualitative | Case study | Observation | No | No | No |
| 211 | Raw M, McNeill A, Murray R. | Case studies of tobacco dependence treatment in Brazil, England, India, South Africa and Uruguay. | South Africa | 2010 | - | - | Convenience sampling | - | - | - | National | Qualitative | Case study | Self-administered questionnaire and key informant interview | No | No | No |
| 212 | Adebiyi AO, Faseru B, Sangowawa AO, Owoaje ET. | Tobacco use amongst out of school adolescents in a Local Government Area in Nigeria. | Nigeria | 2010 | 215 | Adolescents | Multistage cluster sampling | - | 53% | Community/household | Urban | Quantitative | Cross-sectional | Interview | No | Yes | No |
| 213 | Desalu OO, Iseh KR, Olokoba AB, Salawu FK, Danburam A. | Smokeless tobacco use in adult Nigerian population. | Nigeria | 2010 | 1776 | Adults | Multistage cluster sampling | 40.4 | 50.70% | Community/household | Urban | Quantitative | Cross-sectional | Interview | No | No | No |
| 214 | Ayo-Yusuf OA, Szymanski B. | Factors associated with smoking cessation in South Africa. | South Africa | 2010 | 1896 | Adults aged more than 18 years | Multistage cluster sampling | - | - | Community/household | National | Quantitative | Cross-sectional | Interview | No | Yes | No |
| 215 | Pahl K, Brook DW, Morojele NK, Brook JS. | Nicotine dependence and problem behaviors among urban South African adolescents. | South Africa | 2010 | 731 | Adolescents aged 12 to 17 years | Stratified random sampling | 14.55 | 47.30% | Community/household | Urban | Quantitative | Cross-sectional | Interview | No | Yes | No |
| 216 | Omole OB, Ngobale KN, Ayo-Yusuf OA. | Missed opportunities for tobacco use screening and brief cessation advice in South African primary health care: a cross-sectional study. | South Africa | 2010 | 500 | Adults | Systematic sampling | 47.9 | 24.5 | Health facility | - | Quantitative | Cross-sectional | Interview | No | Yes | No |
| 217 | Mariner DC, Ashley M, Shepperd CJ, Mullard G, Dixon M. | Mouth level smoke exposure using analysis of filters from smoked cigarettes: a study of eight countries. | South Africa | 2010 | 860 | Adults | Purposive sampling | - | - | Community/household | - | Quantitative | Cross-sectional | Observation | No | Yes | No |
| 218 | Jalouli J, Ibrahim SO, Sapkota D, Jalouli MM, Vasstrand EN, Hirsch JM, Larsson PA. | Presence of human papilloma virus, herpes simplex virus and Epstein-Barr virus DNA in oral biopsies from Sudanese patients with regard to toombak use. | Sudan | 2010 | 423 | Adults | Convenience sampling | - | 77.78% | Health facility | Urban | Quantitative | Cross-sectional | Observation | No | No | No |
| 219 | Oshodi OY, Aina OF, Onajole AT. | Substance use among secondary school students in an urban setting in Nigeria: prevalence and associated factors. | Nigeria | 2010 | 402 | Students aged 11 to 20 years | Multistage stratified sampling | 15.9 | 43.50% | Schools | Urban | Quantitative | Cross-sectional | Self-administered questionnaire | No | No | No |
| 220 | Rudatsikira E, Muula AS, Siziya S. | Current use of smokeless tobacco among adolescents in the Republic of Congo | Republic of Congo | 2010 | 3034 | Adolescents | Multistage cluster sampling | 13 | - | Schools | National | Quantitative | Cross-sectional | Self-administered questionnaire | No | No | Yes |
| 221 | Peltzer K. | Leisure time physical activity and sedentary behavior and substance use among in-school adolescents in eight African countries. | Botswana, Kenya, Namibia, Senegal, Swaziland, Uganda, Zambia, and Zimbabwe | 2010 | 24,593 | School going adolescents aged 13 to 15 years | Multistage cluster sampling | - | 57.10% | Schools | National | Quantitative | Cross-sectional | Self-administered questionnaire | No | Yes | No |
| 222 | Bandason T, Rusakaniko S. | Prevalence and associated factors of smoking among secondary school students in Harare Zimbabwe. | Zimbabwe | 2010 | 650 | School going adolescents | Multistage stratified sampling | 15.9 | 53% | Schools | Urban | Quantitative | Cross-sectional | Self-administered questionnaire | No | Yes | No |
| 223 | Doku D, Koivusilta L, Raisamo S, Rimpelä A. | Do socioeconomic differences in tobacco use exist also in developing countries? A study of Ghanaian adolescents. | Ghana | 2010 | 1165 | Adolescents | Random sampling | 15.8 | 41.50% | Schools | Both | Quantitative | Cross-sectional | Self-administered questionnaire | No | Yes | No |
| 224 | Resnicow K, Zhang N, Vaughan RD, Reddy SP, James S, Murray DM. | When intraclass correlation coefficients go awry: a case study from a school-based smoking prevention study in South Africa. | South Africa | 2010 | 5266 | Adolescents | Multistage random cluster sampling | 14.1 | 51% | Schools | Both | Quantitative | Randomized controlled trial | Self-administered questionnaire | No | No | No |
| 225 | Chomba E, Tshefu A, Onyamboko M, Kaseba-Sata C, Moore J, McClure EM, Moss N, Goco N, Bloch M, Goldenberg RL. | Tobacco use and secondhand smoke exposure during pregnancy in two African countries: Zambia and the Democratic Republic of the Congo. | Zambia and Democratic Republic of Congo | 2010 | 1756 | Adults aged 18-46 years | Convenience sampling | 26.1 | 0% | Health facility | Urban | Quantitative | Cross-sectional | Self-administered questionnaire | No | Yes | No |
| 226 | Everett-Murphy K, Steyn K, Mathews C, Petersen Z, Odendaal H, Gwebushe N, Lombard C. | The effectiveness of adapted, best practice guidelines for smoking cessation counseling with disadvantaged, pregnant smokers attending public sector antenatal clinics in Cape Town, South Africa. | South Africa | 2010 | 979 | Pregnant women | Convenience sampling | - | 0% | Health facility | Urban | Quantitative | Quasi-experimental | Interview | Yes | Yes | No |
| 227 | Tumwine J. | Implementation of the framework convention on tobacco control in Africa: current status of legislation. | - | 2011 | - | Documents | Purposive sampling | - | - | - | - | Qualitative | Case study | Administrative data/document review | No | Yes | No |
| 228 | Owusu-Dabo E, Lewis S, McNeill A, Gilmore A, Britton J | Support for smoke-free policy, and awareness of tobacco health effects and use of smoking cessation therapy in a developing country | Ghana | 2011 | 6258 | Adolescents and adults aged 14 years and more | Multistage cluster sampling | - | - | Community/household | Both | Qualitative | Case study | Interview | No | Yes | No |
| 229 | Titeca K, Joossens L, Raw M. | Blood cigarettes: cigarette smuggling and war economies in central and eastern Africa | Uganda, Democratic Republic of Congo, and Sudan | 2011 | 404 | - | Purposive sampling | - | - | Community/household | National | Qualitative | Thematic analysis | Interview | No | No | No |
| 230 | Everett-Murphy K, Paijmans J, Steyn K, Matthews C, Emmelin M, Peterson Z. | Scolders, carers or friends: South African midwives' contrasting styles of communication when discussing smoking cessation with pregnant women. | South Africa | 2011 | 24 | Adults | Purposive sampling | 38 | 0% | Workplace | Urban | Qualitative | Thematic analysis | Interview | No | No | No |
| 231 | Otañez M, Glantz SA. | Social responsibility in tobacco production? Tobacco companies' use of green supply chains to obscure the real costs of tobacco farming. | Tanzania and Malawi | 2011 | 160 | Adults and documents | - | - | - | - | National | Qualitative | Case study | Interview and Administrative data/document review | No | No | No |
| 232 | Adeyemi BF, Olusanya AA, Lawoyin JO. | Oral squamous cell carcinoma, socioeconomic status and history of exposure to alcohol and tobacco. | Nigeria | 2011 | 138 | Children and adult patients | Purposive sampling | - | 52.20% | Health facility | Urban | Quantitative | Case control | Administrative data/document review | No | Yes | No |
| 233 | Desalu OO. | Prevalence of chronic bronchitis and tobacco smoking in some rural communities in Ekiti state, Nigeria. | Nigeria | 2011 | 391 | Adults aged 35 years or more | Multistage cluster sampling | 55.5 | 34.50% | Community/household | Rural | Quantitative | Cross-sectional | Interview | No | Yes | No |
| 234 | Brook DW, Rubenstone E, Zhang C, Morojele NK, Brook JS. | Environmental stressors, low well-being, smoking, and alcohol use among South African adolescents. | South Africa | 2011 | 2195 | Adolescents aged 12 to 17 years | Multistage stratified sampling | 14.6 | 45.70% | Community/household | National | Quantitative | Cross-sectional | Interview | No | Yes | No |
| 235 | Desalu OO, Onyedum CC, Adewole OO, Fawibe AE, Salami AK. | Secondhand smoke exposure among nonsmoking adults in two Nigerian cities. | Nigeria | 2011 | 585 | Adults | Multistage stratified sampling | 32.9 | 65.80% | Community/household | Urban | Quantitative | Cross-sectional | Interview | No | Yes | No |
| 236 | Araújo C, Silva-Matos C, Damasceno A, Gouveia ML, Azevedo A, Lunet N. | Manufactured and hand-rolled cigarettes and smokeless tobacco consumption in Mozambique: regional differences at early stages of the tobacco epidemic | Mozambique | 2011 | 12891 | Adults aged 25 to 64 years | Multistage stratified sampling | - | 39.12% | Community/household | National | Quantitative | Cross-sectional | Interview | No | Yes | No |
| 237 | Lunet N, Araújo C, Silva-Matos C, Damasceno A, Gouveia L, Azevedo A. | Changing patterns of tobacco consumption in Mozambique: evidence from a migrant study. | Mozambique | 2011 | 12891 | Adults aged 25 to 64 years | Multistage stratified sampling | - | 39.12% | Community/household | National | Quantitative | Cross-sectional | Interview | No | Yes | No |
| 238 | Padrão P, Silva-Matos C, Damasceno A, Lunet N. | Association between tobacco consumption and alcohol, vegetable and fruit intake across urban and rural areas in Mozambique. | Mozambique | 2011 | 12614 | Adults aged 25 to 64 years | Multistage stratified sampling | - | 39.02% | Community/household | National | Quantitative | Cross-sectional | Interview | No | Yes | No |
| 239 | Shapiro AE, Tshabangu N, Golub JE, Martinson NA. | Intention to quit smoking among human immunodeficiency virus infected adults in Johannesburg, South Africa. | South Africa | 2011 | 150 | Adults aged 18 and more | - | 37 | 66% | Health facility | Urban | Quantitative | Cross-sectional | Interview | No | Yes | No |
| 240 | Hauli KA, Ndetei DM, Jande MB, Kabangila R. | The prevalence of substance use among psychiatric patients: the case study of Bugando Medical centre, Mwanza (northern Tanzania). | Tanzania | 2011 | 184 | Adolescents and adults aged 13 and more | Purposive sampling | 34.8 | 70.70% | Health facility | Urban | Quantitative | Cross-sectional | Interview | No | Yes | No |
| 241 | Brunet L, Pai M, Davids V, Ling D, Paradis G, Lenders L, Meldau R, van Zyl Smit R, Calligaro G, Allwood B, Dawson R, Dheda K. | High prevalence of smoking among patients with suspected tuberculosis in South Africa. | South Africa | 2011 | 424 | Adults | Purposive sampling | 39.5 | 67% | Health facility | Urban | Quantitative | Cross-sectional | Interview | No | Yes | No |
| 242 | Fakier N, Wild LG. | Associations among sleep problems, learning difficulties and substance use in adolescence. | South Africa | 2011 | 703 | Adolescents and young adults aged 13 to 20 years | Purposive sampling | - | - | Schools | Urban | Quantitative | Cross-sectional | Interview | No | Yes | No |
| 243 | Zatu MC, Van Rooyen JM, Schutte AE. | Smoking and vascular dysfunction in Africans and Caucasians from South Africa. | South Africa | 2011 | 630 | Adults aged 20 to 70 years | - | 41.6 for africans and 40.4 for caucasians | 45.71% | - | Urban | Quantitative | Cross-sectional | Observation | No | Yes | No |
| 244 | Jalouli MM, Jalouli J, Sapkota D, Ibrahim SO, Sand L, Hirsch JM. | Differential expression of apoptosis, cell cycle regulation and intermediate filament genes in oral squamous cell carcinomas associated with toombak use in Sudan. | Sudan | 2011 | 26 | Adult patients | Convenience sampling | 54.9 | 88% | Health facility | Urban | Quantitative | Cross-sectional | Observation | No | No | No |
| 245 | Bolliger CT, Issa JS, Posadas-Valay R, Safwat T, Abreu P, Correia EA, Park PW, Chopra P. | Effects of varenicline in adult smokers: a multinational, 24-week, randomized, double-blind, placebo-controlled study. | South Africa | 2011 | 588 | Adults aged 18 to 75 years | Random sampling | - | 60.37% | Health facility | - | Quantitative | Randomized control trial | Observation | No | Yes | No |
| 246 | Viswanathan B, Plumettaz C, Gedeon J, Bovet P. | Impact of a smoking ban in public places: a rapid assessment in the Seychelles. | Seychelles | 2011 | 38 hospitality venues and 163 adults | Hospitality venues and adults | Convenience sampling | - | - | Community/household and recreational facilities | Urban | Quantitative | Cross-sectional | Observation and Interview | No | No | No |
| 247 | Agbenyikey W, Wellington E, Gyapong J, Travers MJ, Breysse PN, McCarty KM, Navas-Acien A. | Secondhand tobacco smoke exposure in selected public places (PM2.5 and air nicotine) and non-smoking employees (hair nicotine) in Ghana | Ghana | 2011 | - | - | Convenience sampling | - | - | - | Urban | Quantitative | Cross-sectional | Self-administered questionnaire | No | No | No |
| 248 | El-Amin Sel-T, Nwaru BI, Ginawi I, Pisani P, Hakama M. | The role of parents, friends and teachers in adolescents' cigarette smoking and tombak dipping in Sudan. | Sudan | 2011 | 4277 | Adolescents | Multistage cluster sampling | - | 53% | Schools | Both | Quantitative | Cross-sectional | Self-administered questionnaire | No | No | Yes |
| 249 | Fawibe AE, Shittu AO. | Prevalence and characteristics of cigarette smokers among undergraduates of the University of Ilorin, Nigeria. | Nigeria | 2011 | 1754 | Individuals aged 16-43years | Stratified random sampling | 21.6 | 65.50% | Schools | Urban | Quantitative | Cross-sectional | Self-administered questionnaire | No | Yes | No |
| 250 | Uti OG, Sofola OO. | Smoking cessation counseling in dentistry: attitudes of Nigerian dentists and dental students. | Nigeria | 2011 | 136 | Adults | Convenience sampling | - | 52.20% | School and workplace | Urban | Quantitative | Cross-sectional | Self-administered questionnaire | No | Yes | No |
| 251 | Peltzer K. | Early smoking initiation and associated factors among in-school male and female adolescents in seven African countries | Botswana, Kenya, Namibia, Senegal, Tanzania, Uganda, and Zimbabwe | 2011 | 17725 | Adolescents aged 13-15 years old | - | - | 50% | Schools | National | Quantitative | Cross-sectional | Self-administered questionnaire | Yes | Yes | No |
| 252 | Deressa W, Azazh A. | Substance use and its predictors among undergraduate medical students of Addis Ababa University in Ethiopia | Ethiopia | 2011 | 622 | Adolescents and adults aged 15 or more | Convenience sampling | - | 68.50% | Schools | Urban | Quantitative | Cross-sectional | Self-administered questionnaire | No | No | No |
| 253 | Steyl T, Phillips J. | Actual and perceived substance use of health science students at a university in the Western Cape, South Africa. | South Africa | 2011 | 201 | Adult medical students | Convenience sampling | 22.16 | - | Schools | Urban | Quantitative | Cross-sectional | Self-administered questionnaire | No | No | No |
| 254 | Mashita RJ, Themane MJ, Monyeki KD, Kemper HC. | Current smoking behaviour among rural South African children: Ellisras Longitudinal Study. | South Africa | 2011 | 1654 | Adolescents | Multistage cluster sampling | - | 51.63% | Schools | Rural | Quantitative | Cross-sectional | Self-administered questionnaire | No | No | No |
| 255 | Alwan H, Viswanathan B, Rousson V, Paccaud F, Bovet P. | Association between substance use and psychosocial characteristics among adolescents of the Seychelles. | Seychelles | 2011 | 1417 | School going adolescents aged 11 to 17 years | Multistage cluster sampling | 14 | 47.77% | Schools | National | Quantitative | Cross-sectional | Self-administered questionnaire | No | Yes | No |
| 256 | Gedikondele JS, Longo-Mbenza B, Nzanza JM, Luila EL, Reddy P, Buso D | Nose and throat complications associated with passive smoking among Congolese school children | Democratic Republic of Congo | 2011 | 381 | Adolescents | Multistage stratified sampling | 9.8 | 50.90% | Schools | Urban | Quantitative | Cross-sectional | Self-administered questionnaire | No | No | No |
| 257 | Heydari G, Talischi F, Masjedi MR, Alguomani H, Joossens L, Ghafari M. | Comparison of tobacco control policies in the Eastern Mediterranean countries based on Tobacco Control Scale scores. | Djibouti, Somalia, and Sudan | 2011 | - | Adults | Purposive sampling | - | - | Workplace | - | Quantitative | Cross-sectional | Self-administered questionnaire | No | No | No |
| 258 | Sharp EH, Coffman DL, Caldwell LL, Smith EA, Wegner L, Vergnani T, Mathews C. | Predicting substance use behavior among South African adolescents: The role of leisure experiences across time. | South Africa | 2011 | 1118 | School going adolescents | Multistage cluster sampling | 13.98 | 49% | Schools | Urban | Quantitative | Cohort | Self-administered questionnaire | No | No | No |
| 259 | Peltzer K. | Determinants of exposure to second-hand tobacco smoke (SHS) among current non-smoking in-school adolescents (aged 11-18 years) in South Africa: results from the 2008 GYTS study. | South Africa | 2011 | 6412 | Adolescents aged 11 to 18 years | Multistage cluster sampling | - | 44.60% | Schools | National | Quantitative | Cross-sectional | Self-administered questionnaire | No | Yes | Yes |
| 260 | Nwafor CC, Ibeh CC, Aguwa EN, Chukwu JN. | Assessment of pattern of cigarette smoking and associated factors among male students in public secondary schools in Anambra State, Nigeria. | Nigeria | 2012 | 850 | School going adolescents | Multistage cluster sampling | 16 | 100% | Schools | Both | Mixed | Cross-sectional | Self-administered questionnaire and Focus group discussion | No | No | No |
| 261 | Petersen Z, Nilsson M, Steyn K, Emmelin M. | Identifying with a process of change: a qualitative assessment of the components included in a smoking cessation intervention at antenatal clinics in South Africa. | South Africa | 2012 | - | - | Purposive sampling | - | 0% | Health facility | Urban | Qualitative | Thematic analysis | Focus group discussion | No | No | No |
| 262 | Troost JP, Barondess DA, Storr CL, Wells JE, Obaid Al-Hamzawi A, Andrade LH, Bromet E, Bruffaerts R, Florescu S, de Girolamo G, de Graaf R, Gureje O, Haro JM, Hu C, Huang Y, Karam AN, Kessler RC, Lepine JP, Matschinger H, Medina-Mora ME, O'Neill S, Posada-Villa J, Sagar R, Takeshima T, Tomov T, Williams DR, Anthony JC. | An updated global picture of cigarette smoking persistence among adults. | Nigeria and South Africa | 2012 | - | Adults | Multistage cluster sampling | - | - | Community/household | - | Quantitative | Cross-sectional | Interview | No | No | No |
| 263 | John RM, Mamudu HM, Liber AC. | Socioeconomic implications of tobacco use in Ghana | Ghana | 2012 | 9484 | Adults (Women aged 15-49 years and men aged 15-59 years) | Multistage cluster sampling | - | 48.16% | Community/household | National | Quantitative | Cross-sectional | Interview | No | Yes | No |
| 264 | Tafawa AO, Viswanath K, Kawachi I, Williams DR. | Mass media exposure, social stratification, and tobacco consumption among Nigerian adults. | Nigeria | 2012 | 47805 | Adults | Multistage stratified cluster sampling | - | 32% | Community/household | National | Quantitative | Cross-sectional | Interview | No | Yes | No |
| 265 | Harper S, McKinnon B. | Global socioeconomic inequalities in tobacco use: internationally comparable estimates from the World Health Surveys. | Burkina Faso, Chad, Comoros, The Republic of Congo, Cote d'Ivore, Ethiopia, Ghana, Kenya, Malawi, Mali, Mauritania, Mauritius, Namibia, Senegal, South Africa, Swaziland, Zambia, and Zimbabwe | 2012 | - | Adults aged 18 or more | Stratified random sampling | Burkina Faso: 37.0, Chad: 36.5, Comoros: 40.7, Congo: 35.3, Cote d'Ivore: 34.7, Ethiopia: 35.6, Ghana: 40.1, Kenya: 35.3, Malawi: 35.5, Mali: 40.1, mauritania: 37.5, Mauritius: 41.2, Namibia: 37.4, Senegal: 37.9, South Africa: 37.6, Swaziland: 38.9, Zambia: 35.4, and Zimbabwe: 37.1 | Burkina Faso: 47.4, Chad: 50, Comoros: 49.1, Congo: 47.7, Cote d'Ivore: 57.8, Ethiopia: 50.3, Ghana: 45.5, Kenya: 49.6, Malawi: 44, Mali: 65.9, mauritania: 44, Mauritius: 49.6, Namibia: 42.6, Senegal: 55.5, South Africa: 47.6, Swaziland: 47, Zambia: 47.3, and Zimbabwe: 39.9 | Community/household | National | Quantitative | Cross-sectional | Interview | No | Yes | No |
| 266 | Mutihir J, Musa J, Daru P, Nyango D, Audu M. | Substance abuse among antenatal patients at jos university teaching hospital, north central Nigeria. | Nigeria | 2012 | 557 | Individuals aged 15 to 48 years | Purposive sampling | 29.1 | 0% | Health facility | Urban | Quantitative | Cross-sectional | Interview | No | Yes | No |
| 267 | Iliyasu Z, Gajida AU, Abubakar IS, Shittu O, Babashani M, Aliyu MH. | Patterns and predictors of cigarette smoking among HIV-infected patients in northern Nigeria. | Nigeria | 2012 | 296 | Adults | Systematic sampling | 37.5 | 71.60% | Health facility | Urban | Quantitative | Cross-sectional | Interview | No | Yes | No |
| 268 | Vogelsang M, Wang Y, Veber N, Mwapagha LM, Parker MI. | The cumulative effects of polymorphisms in the DNA mismatch repair genes and tobacco smoking in oesophageal cancer risk. | South Africa | 2012 | 1160 | Adults | - | - | - | - | - | Quantitative | Cross-sectional | Observation | No | No | No |
| 269 | Ureme S, Njoku O, Ejezie F, Ibeh B, Ikekpazu E, Mba M. | Plasma concentration of ascorbic Acid and some hematological parameters in tobacco snuffers among the igbos of southeastern Nigeria. | Nigeria | 2012 | 100 | Adults | Purposive sampling | - | 60% | Community/household | Urban | Quantitative | Cross-sectional | Observation | No | Yes | No |
| 270 | Aabye MG, Hermansen TS, Ruhwald M, Praygod G, Faurholt-Jepsen D, Jeremiah K, Faurholt-Jepsen M, Range N, Friis H, Changalucha J, Andersen AB, Ravn P. | Negative effect of smoking on the performance of the QuantiFERON TB gold in tube test. | Tanzania | 2012 | 172 | Adults | Purposive sampling | - | - | Health facility | National | Quantitative | Cross-sectional | Observation | No | Yes | No |
| 271 | Oni T, Gideon HP, Bangani N, Tsekela R, Seldon R, Wood K, Wilkinson KA, Goliath RT, Ottenhoff TH, Wilkinson RJ. | Smoking, BCG and employment and the risk of tuberculosis infection in HIV-infected persons in South Africa. | South Africa | 2012 | 335 | Adults | Systematic sampling | - | 15.10% | Health facility | Urban | Quantitative | Cross-sectional | Observation | No | Yes | No |
| 272 | Merghani TH, Saeed A, Alawad A. | Changes in plasma IL4, TNFá and CRP in response to regular passive smoking at home among healthy school children in Khartoum, Sudan. | Sudan | 2012 | 135 | School going adolescents | Random sampling | - | 100% | Schools | Urban | Quantitative | Cross-sectional | Observation | No | Yes | No |
| 273 | Ibrahim MM, Khalil AA, Khan UA. | Offspring sex ratios among male tobacco smokers in Khartoum, Sudan. | Sudan | 2012 | 458 | Adults | Convenience sampling | - | 100% | Community/household | Urban | Quantitative | Cross-sectional | Self-administered questionnaire | No | No | No |
| 274 | Ehizele AO, Azodo CC, Ojehanon PI, Akhionbare O, Umoh AO, Adeghe HA | Prevalence of tobacco use among dental patients and their knowledge of its health effects | Nigeria | 2012 | 400 | Adults | Convenience sampling | 29.8 | 58% | Health facility | Urban | Quantitative | Cross-sectional | Self-administered questionnaire | No | No | No |
| 275 | Rantao M, Ayo-Yusuf OA. | Dual use of cigarettes and smokeless tobacco among South African adolescents. | South Africa | 2012 | 1878 | Adolescents | Multistage cluster sampling | 14.6 | 49.70% | Schools | Both | Quantitative | Cross-sectional | Self-administered questionnaire | No | Yes | No |
| 276 | Senkubuge F, Ayo-Yusuf OA, Louwagie GM, Okuyemi KS. | Water pipe and smokeless tobacco use among medical students in South Africa. | South Africa | 2012 | 722 | Individuals aged 17 to 50 years | Purposive sampling | 23 | 47% | Schools | Urban | Quantitative | Cross-sectional | Self-administered questionnaire | No | Yes | No |
| 277 | Poms LW, Fleming LC, Jacobsen KH. | Parenting practices and tobacco use in middle school students in low- and middle-income countries. | Botswana, Kenya, Mauritius, Namibia, Senegal, Seychelles, Tanzania, Uganda, Zimbabwe, and Djibouti | 2012 | 106041 | Adolescents | Multistage cluster sampling | - | - | Schools | National | Quantitative | Cross-sectional | Self-administered questionnaire | No | No | No |
| 278 | Agaku I, Akinyele AO, Omaduvie UT. | Evaluation of factors influencing intention to quit smokeless and cigarette tobacco use among Nigerian adolescents. | Nigeria | 2012 | 536 | School going adolescents | Multistage cluster sampling | - | - | Schools | Both | Quantitative | Cross-sectional | Self-administered questionnaire | No | No | No |
| 279 | Gadalla YM, Adil AM, Mustafa BM, Abdo H. | Prevalence of smoking among school adolescents in Khartoum State. | Sudan | 2012 | 910 | School going adolescents aged 11 to 17 years | Multistage cluster sampling | - | 100% | Schools | Urban | Quantitative | Cross-sectional | Self-administered questionnaire | No | Yes | No |
| 280 | Reda AA, Moges A, Yazew B, Biadgilign S. | Determinants of cigarette smoking among school adolescents in eastern Ethiopia: a cross-sectional stud | Ethiopia | 2012 | 1721 | Adolescents and youths aged 15 to 25 years | Proportional stratified sampling | 16.4 | 50.10% | Schools | Urban | Quantitative | Cross-sectional | Self-administered questionnaire | No | No | No |
| 281 | Doku D, Raisamo S, Wiium N. | The role of tobacco promoting and restraining factors in smoking intentions among Ghanaian youth. | Ghana | 2012 | 1338 | Adolescents and young adults aged 12 to 20 years | Random sampling | - | - | Schools | Both | Quantitative | Cross-sectional | Self-administered questionnaire | No | Yes | No |
| 282 | Doku D, Koivusilta L, Raisamo S, Rimpelä A. | Tobacco use and exposure to tobacco promoting and restraining factors among adolescents in a developing country. | Ghana | 2012 | 1165 | Adolescents aged 13 to 18 years | Stratified random sampling | - | 41.46% | Schools | Both | Quantitative | Cross-sectional | Self-administered questionnaire | No | Yes | No |
| 283 | Pretorius E. | Ultrastructural changes in platelet membranes due to cigarette smoking. | South Africa | 2012 | 35 | Adults aged 20 to 60 years | Purposive sampling | - | - | - | - | Quantitative | Cross-sectional | Observation | No | Yes | No |
| 284 | Gilreath TD, Chaix B, King G, Matthews S, Flisher AJ. | Multi-level influence of school norms on tobacco use in South Africa: an ecometric consideration of group differences. | South Africa | 2012 | 1277 | Adolescents | Multistage cluster sampling | 15.7 | 43.10% | Schools | Urban | Quantitative | Cross-sectional | Self-administered questionnaire | No | No | No |
| 285 | Saito J, Nonaka D, Mizoue T, Kobayashi J, Jayatilleke AC, Shrestha S, Kikuchi K, Haque SE, Yi S, Ayi I, Jimba M. | Limited potential of school textbooks to prevent tobacco use among students grade 1-9 across multiple developing countries: a content analysis study. | Benin, Ghana, Niger, and Zambia | 2013 | - | Textbooks | Purposive sampling | - | - | - | National | Qualitative | Thematic analysis | Observation | No | Yes | No |
| 286 | Oladele D, Clark AM, Richter S, Laing L. | Critical realism: a practical ontology to explain the complexities of smoking and tobacco control in different resource settings. | Nigeria | 2013 | 42 | Adults | Purposive sampling | - | - | Community/household | Urban | Qualitative | Grounded theory | Observation, Indepth interview, focus group discussion | No | Yes | No |
| 287 | Sturm R, An R, Maroba J, Patel D. | The effects of obesity, smoking, and excessive alcohol intake on healthcare expenditure in a comprehensive medical scheme. | South Africa | 2013 | 69380 | Adults | Convenience sampling | - | 50% | - | - | Quantitative | Cross-sectional | Administrative data/document review and Self-administered questionnaire | No | Yes | No |
| 288 | Yawson AE, Baddoo A, Hagan-Seneadza NA, Calys-Tagoe B, Hewlett S, Dako-Gyeke P, Mensah G, Minicuci N, Naidoo N, Chatterji S, Kowal P, Biritwum R | Tobacco use in older adults in Ghana: sociodemographic characteristics, health risks and subjective wellbeing. | Ghana | 2013 | 4252 | Adults aged 50 years and above | - | - | - | Community/household | National | Quantitative | Cross-sectional | Interview | No | No | No |
| 289 | Lo TQ, Oeltmann JE, Odhiambo FO, Beynon C, Pevzner E, Cain KP, Laserson KF, Phillips-Howard PA. | Alcohol use, drunkenness and tobacco smoking in rural western Kenya. | Kenya | 2013 | 72292 | Adults | Census | - | 43.10% | Community/household | Rural | Quantitative | Cross-sectional | Interview | No | No | No |
| 290 | Trenz RC, Scherer M, Duncan A, Harrell PT, Moleko AG, Latimer WW. | Latent class analysis of polysubstance use, sexual risk behaviors, and infectious disease among South African drug users. | South Africa | 2013 | 409 | Adults aged 18 to 40 years | Convenience sampling | 26.66 | 49.60% | Community/household | Urban | Quantitative | Cross-sectional | Interview | No | Yes | No |
| 291 | Adepoju EG, Olowookere SA, Adeleke NA, Afolabi OT, Olajide FO, Aluko OO. | A population based study on the prevalence of cigarette smoking and smokers' characteristics at osogbo, Nigeria. | Nigeria | 2013 | 759 | Adults | Multistage cluster sampling | 42.1 | 48% | Community/household | Urban | Quantitative | Cross-sectional | Interview | No | No | No |
| 292 | Doku D, Darteh EK, Kumi-Kyereme A. | Socioeconomic inequalities in cigarette smoking among men: evidence from the 2003 and 2008 Ghana demographic and health surveys. | Ghana | 2013 | 19166 | Individuals aged 15 to 59 years | Multistage cluster sampling | - | 100% | Community/household | National | Quantitative | Cross-sectional | Interview | No | Yes | No |
| 293 | Borzekowski DL, Cohen JE. | International reach of tobacco marketing among young children. | Nigeria | 2013 | 385 | Children aged 5 or 6 years | Multistage cluster sampling | - | 50.10% | Community/household | Both | Quantitative | Cross-sectional | Interview | No | No | No |
| 294 | Ayo-Yusuf OA, Olutola BG. | 'Roll-your-own' cigarette smoking in South Africa between 2007 and 2010. | South Africa | 2013 | 6019 | Individuals aged 16 or more | Multistage probability sampling | - | 48.20% | Community/household | National | Quantitative | Cross-sectional | Interview | No | Yes | No |
| 295 | Padrão P, Damasceno A, Silva-Matos C, Carreira H, Lunet N. | Tobacco consumption in Mozambique: use of distinct types of tobacco across urban and rural settings. | Mozambique | 2013 | 3304 | Adults aged 25 to 64 years | Multistage stratified sampling | - | 41.79% | Community/household | National | Quantitative | Cross-sectional | Interview | No | No | No |
| 296 | Reda AA, Kotz D, Biadgilign S. | Adult tobacco use practice and its correlates in eastern Ethiopia: a cross-sectional study | Ethiopia | 2013 | - | Adolescents and adults aged 15 years and more | Random sampling | 35 | 75.10% | Community/household | Rural | Quantitative | Cross-sectional | Interview | No | No | No |
| 297 | Louwagie GM, Ayo-Yusuf OA. | Tobacco use patterns in tuberculosis patients with high rates of human immunodeficiency virus co-infection in South Africa. | South Africa | 2013 | 1926 | Adults | - | - | 52.30% | Health facility | Urban | Quantitative | Cross-sectional | Interview | No | Yes | No |
| 298 | Waweru P, Anderson R, Steel H, Venter WD, Murdoch D, Feldman C. | The prevalence of smoking and the knowledge of smoking hazards and smoking cessation strategies among HIV- positive patients in Johannesburg, South Africa. | South Africa | 2013 | 207 | Adults | Convenience sampling | 39.9 | 47.80% | Health facility | Urban | Quantitative | Cross-sectional | Interview | No | No | No |
| 299 | Maina WK, Kitonyo R, Ogwell AE. | Using findings from a public opinion poll to build political support for tobacco control policy in Kenya. | Kenya | 2013 | 2021 | Adults | Multistage stratified sampling | - | 49% | Community/household | National | Quantitative | Cross-sectional | Interview | No | Yes | No |
| 300 | Odukoya OO, Sekoni AO, Onajole AT, Upadhyay RP. | Alcohol consumption and cigarette smoking pattern among brothel-based female sex workers in two local government areas in Lagos state, Nigeria. | Nigeria | 2013 | 323 | Adults | Purposive sampling | 28.1 | 0% | Workplace | Both | Quantitative | Cross-sectional | Interview | No | Yes | No |
| 301 | Anass MA, G Ahmed H. | A Case-Control Study of Oral Epithelial Proliferative Markers among Sudanese Toombak Dippers Using Micronuclei Assay, Argyrophilic Nucleolar Organizer Region, Papanicolaou and Crystal Violet Methods. | Sudan | 2013 | 210 | Individuals aged 16 to 94 years | - | 33 | - | - | - | Quantitative | Cross-sectional | Observation | No | No | No |
| 302 | Orisakwe OE, Igweze ZN, Okolo KO, Ajaezi GC. | Heavy metal hazards of Nigerian smokeless tobacco. | Nigeria | 2013 | 30 | Smokeless tobacco | - | - | - | Community/household | - | Quantitative | Cross-sectional | Observation | No | No | No |
| 303 | Peer N, Bradshaw D, Laubscher R, Steyn N, Steyn K. | Urban-rural and gender differences in tobacco and alcohol use, diet and physical activity among young black South Africans between 1998 and 2003. | South Africa | 2013 | 5252 | Adolescents and young adults aged 15 to 24 years | Multistage cluster sampling | - | 47% | Community/household | National | Quantitative | Cross-sectional | Observation | No | No | No |
| 304 | Du Plooy JN, Buys A, Duim W, Pretorius E. | Comparison of platelet ultrastructure and elastic properties in thrombo-embolic ischemic stroke and smoking using atomic force and scanning electron microscopy. | South Africa | 2013 | - | Adults | Purposive sampling | - | - | Health facility | - | Quantitative | Cross-sectional | Observation | No | Yes | No |
| 305 | PrayGod G, Range N, Faurholt-Jepsen D, Jeremiah K, Faurholt-Jepsen M, Aabye MG, Magnussen P, Changalucha J, Andersen AB, Wells JC, Friis H. | Sex, smoking, and socioeconomic status are associated with body composition among tuberculosis patients in a deuterium dilution cross-sectional study in Mwanza, Tanzania. | Tanzania | 2013 | 201 | Individuals aged 15 or more | Purposive sampling | 36.8 | 62.20% | Health facility | Urban | Quantitative | Cross-sectional | Observation | No | Yes | No |
| 306 | Akaji EA, Folaranmi N. | Tobacco use and oral health of inmates in a Nigerian prison. | Nigeria | 2013 | 230 | Adults | Systematic sampling | 28.48 | 97.40% | Prison | Urban | Quantitative | Cross-sectional | Observation | No | No | No |
| 307 | Merghani TH, Saeed AM. | The relationship between regular second-hand smoke exposure at home and indictors of lung function in healthy school boys in Khartoum. | Sudan | 2013 | 135 | Adolescents | Multistage cluster sampling | 11.5 | 100% | Schools | Urban | Quantitative | Cross-sectional | Observation | No | Yes | No |
| 308 | Peer N, Bradshaw D, Laubscher R, Steyn N, Steyn K. | Urban-rural and gender differences in tobacco and alcohol use, diet and physical activity among young black South Africans between 1998 and 2003. | South Africa | 2013 | 5252 | Individuals aged 15 and more | Multistage cluster sampling | - | 47% | Community/household | National | Quantitative | Cross-sectional | Observation | No | No | No |
| 309 | Monyeki KD, Kemper HC, Amusa LO, Motshwane M. | Advertisement and knowledge of tobacco products among Ellisras rural children aged 11 to 18 years: Ellisras Longitudinal study. | South Africa | 2013 | 1654 | Adolescents | - | - | 51.63% | - | Rural | Quantitative | Cross-sectional | Self-administered questionnaire | No | No | No |
| 310 | Ahmed HG. | Survey on knowledge and attitudes related to the relation between tobacco, alcohol abuse and cancer in the northern state of Sudan. | Sudan | 2013 | 207 | Adults | Convenience sampling | 34.12 | 97% | Community/household | Both | Quantitative | Cross-sectional | Self-administered questionnaire | No | No | No |
| 311 | Lam C, Martinson N, Hepp L, Ambrose B, Msandiwa R, Wong ML, Apelberg B, Tamplin S, Golub JE. | Prevalence of tobacco smoking in adults with tuberculosis in South Africa. | South Africa | 2013 | 707 | Adults aged 18 years and more | Convenience sampling | - | 46% | Health facility | Urban | Quantitative | Cross-sectional | Self-administered questionnaire | No | Yes | No |
| 312 | Odukoya OO, Odeyemi KA, Oyeyemi AS, Upadhyay RP. | Determinants of smoking initiation and susceptibility to future smoking among school-going adolescents in Lagos State, Nigeria. | Nigeria | 2013 | 973 | School going adolescents and young adults aged 10-21 years | Random sampling | 14.2 | 52.90% | Schools | Urban | Quantitative | Cross-sectional | Self-administered questionnaire | No | No | No |
| 313 | Mbatchou Ngahane BH, Luma H, Mapoure YN, Fotso ZM, Afane Ze E. | Correlates of cigarette smoking among university students in Cameroon | Cameroon | 2013 | 3000 | Adults | Convenience sampling | 23.3 | 62% | Schools | Urban | Quantitative | Cross-sectional | Self-administered questionnaire | No | No | No |
| 314 | Daniels KE, Roman NV. | A descriptive study of the perceptions and behaviors of waterpipe use by university students in the Western Cape, South Africa. | South Africa | 2013 | 389 | Adult university students | Multistage cluster sampling | 22.2 | 36% | Schools | - | Quantitative | Cross-sectional | Self-administered questionnaire | No | No | No |
| 315 | Carney T, Myers BJ, Louw J, Lombard C, Flisher AJ. | The relationship between substance use and delinquency among high-school students in Cape Town, South Africa. | South Africa | 2013 | 1470 | School going adolescents | Multistage cluster sampling | 14.1 | 56.40% | Schools | Urban | Quantitative | Cross-sectional | Self-administered questionnaire | No | No | No |
| 316 | Agaku IT, Adisa AO, Akinyamoju AO, Agboola SO. | A cross-country comparison of the prevalence of exposure to tobacco advertisements among adolescents aged 13-15 years in 20 low and middle income countries. | Botswana, Lesotho, Rwanda, Syechelles, South Africa, and Togo | 2013 | Botswana: 2207, Lesotho: 3426, Rwanda: 2284, Syechelles: 1508, South Africa: 8602, and Togo: 4262 | School going adolescents aged 13 to 15 years | Multistage cluster sampling | - | Botswana: 41.6%, Lesotho: 37.5%, Rwanda: 47.2%, Syechelles: 49.4%, South Africa: 42.3%, and Togo: 59.6% | Schools | National | Quantitative | Cross-sectional | Self-administered questionnaire | No | No | Yes |
| 317 | Mamudu HM, Veeranki SP, John RM. | Tobacco use among school-going adolescents (11-17 years) in Ghana | Ghana | 2013 | 9990 | Adolescents aged 11 to 17 years | Multistage cluster sampling | - | 54.60% | Schools | National | Quantitative | Cross-sectional | Self-administered questionnaire | No | Yes | Yes |
| 318 | Atoyebi OA, Ibirongbe DO, Babatunde OA, Atoyebi OE. | To start and quit smoking cigarettes: an evaluation of students in a Nigerian city. | Nigeria | 2013 | 41 | Adults | Multistage random sampling | 21.78 | 100% | Schools | Urban | Quantitative | Cross-sectional | Self-administered questionnaire | No | Yes | No |
| 319 | Gebreslassie M, Feleke A, Melese T. | Psychoactive substances use and associated factors among Axum University students, Axum Town, North Ethiopia. | Ethiopia | 2013 | 764 | Adolescents and adults aged 15 to 30 years | Multistage stratified random sampling | 22.3 | 58.80% | Schools | Urban | Quantitative | Cross-sectional | Self-administered questionnaire | No | Yes | No |
| 320 | Awopeju O, Erhabor G, Awosusi B, Awopeju O, Adewole O, Irabor I. | Smoking prevalence and attitudes regarding its control among health professional students in South-Western Nigeria. | Nigeria | 2013 | 675 | Adult university students | Purposive sampling | - | 51.10% | Schools | Urban | Quantitative | Cross-sectional | Self-administered questionnaire | No | Yes | No |
| 321 | Elamin OE, Elamin SE, Dafalla BA, El-Amin ME, Elsiddig AA. | Cigarette smoking among medical students in The National Ribat University, Sudan. | Sudan | 2013 | 414 | Young adults aged 18 and more | Purposive sampling | - | 37% | Schools | Urban | Quantitative | Cross-sectional | Self-administered questionnaire | No | No | No |
| 322 | Ayo-Yusuf OA, Rantao MM. | Influence of rural non-smoking adolescents' sense of coherence and exposure to household smoking on their commitment to a smoke-free lifestyle. | South Africa | 2013 | 1767 | Adolescents | Multistage cluster sampling | 14.6 | 49.90% | Schools | Rural | Quantitative | Cohort | Self-administered questionnaire | No | No | No |
| 323 | Leykin Y, Aguilera A, Pérez-Stable EJ, Muñoz RF. | Prompting Depression Treatment Seeking among Smokers: A Comparison of Participants from Six Countries in an Internet Stop Smoking RCT. | South Africa | 2013 | 336 | Adults aged 18 and more | Convenience sampling | 34.4 | 51.80% | Community/household | - | Quantitative | Randomized control trial | Self-administered questionnaire | No | Yes | No |
| 324 | Winkler V, Ott JJ, Cowan M, Becher H. | Smoking prevalence and its impacts on lung cancer mortality in Sub-Saharan Africa: an epidemiological study. | Sub-Saharan Africa (Benin, Malawi, Mozambique, Niger, Sierra Leone, and Swaziland) | 2013 | 23733 | Adults more than 25 (more than 15 in one country-Niger) | - | - | - | Community/household | National | Quantitative | Cross-sectional | Interview | No | No | No |
| 325 | Mamudu HM, John RM, Veeranki SP, Ouma AE. | The odd man out in Sub-Saharan Africa: understanding the tobacco use prevalence in Madagascar. | Sub-Saharan Africa and Madagascar | 2013 | 25961 in Madagascar | Individuals aged 15 and more | Multistage cluster sampling | - | - | Community/household | National | Quantitative | Cross-sectional | Interview | No | No | No |
| 326 | Sorsdahl K, Myers B, Ward CL, Matzopoulos R, Mtukushe B, Nicol A, Cuijpers P, Stein DJ. | Adapting a blended motivational interviewing and problem-solving intervention to address risky substance use amongst South Africans. | South Africa | 2014 | 20 | Adult patients aged 18 or more | Purposive sampling | 31 | 55% | Health facility | Urban | Mixed | Non-randomized non control intervention study and Thematic analysis | Interview | No | Yes | No |
| 327 | Murphy K, Steyn K, Mathews C. | The midwife's role in providing smoking cessation interventions for pregnant women: The views of midwives working with high risk, disadvantaged women in public sector antenatal services in South Africa. | South Africa | 2014 | 81 for survey and 24 for interviews | Adults | Convenience sampling | 38.6 | - | Workplace | Urban | Mixed | Cross-sectional | Self-administered questionnaire and Indepth interviews | No | No | No |
| 328 | Singh A, Owusu-Dabo E, Britton J, Munafò MR, Jones LL. | "Pictures don't lie, seeing is believing": exploring attitudes to the introduction of pictorial warnings on cigarette packs in Ghana | Ghana | 2014 | 85 | Adolescents and adults aged 15 and older | Purposive sampling | 38 | 71.76% | School, Hospital, Brothels and abattoirs | Urban | Qualitative | Thematic analysis | Focus group discussion | No | No | No |
| 329 | Tam J, van Walbeek C. | Tobacco control in Namibia: the importance of government capacity, media coverage and industry interference. | Namibia | 2014 | 13 | Adults | Snowball sampling | - | - | Government organizations | National | Qualitative | Case study | Interview | No | No | No |
| 330 | Omole OB, Ayo-Yusuf OA, Ngobale KN. | Implementing tobacco dependence treatment during clinical consultations: a qualitative study of clinicians' experiences, perceptions and behaviours in a South African primary health care setting. | South Africa | 2014 | 19 | Adult physicians and nurses | Purposive sampling | 39.6 | 47.37% | Workplace |  | Qualitative | Thematic analysis | Interview | No | No | No |
| 331 | Egbe CO, Petersen I, Meyer-Weitz A, Oppong Asante K. | An exploratory study of the socio-cultural risk influences for cigarette smoking among Southern Nigerian youth. | Nigeria | 2014 | 27 | Adults | Snowball sampling | - | - | Community/household | Both | Qualitative | Thematic analysis | Interview and Focus group discussion | No | No | No |
| 332 | Ayo-Yusuf OA, Olufajo O, Agaku IT. | Exposure to secondhand smoke and voluntary adoption of smoke-free home and car rules among non-smoking South African adults. | South Africa | 2014 | 3094 | Individuals aged 16 years or more | Multistage stratified probability sampling | - | - | Community/household | National | Quantitative | Cross-sectional | - | No | No | No |
| 333 | van Walbeek C. | Measuring changes in the illicit cigarette market using government revenue data: the example of South Africa. | South Africa | 2014 | - | - | - | - | - | - | National | Quantitative | Cross-sectional (data collected in multiple time points) | Administrative data/document review | No | No | No |
| 334 | Kruse GR, Bangsberg DR, Hahn JA, Haberer JE, Hunt PW, Muzoora C, Bennett JP, Martin JN, Rigotti NA. | Tobacco use among adults initiating treatment for HIV infection in rural Uganda. | Uganda | 2014 | 496 | Adults aged 18 or more | Purposive sampling | 35 | 31% | Health facility | Rural | Quantitative | Cohort | Interview | No | Yes | No |
| 335 | Olowookere SA, Adepoju EG, Gbolahan OO. | Awareness and attitude to the law banning smoking in public places in Osun State, Nigeria. | Nigeria | 2014 | 520 | Adults aged more than 18 years | Convenience sampling | 42.2 | 46% | Community/household | Urban | Quantitative | Cross-sectional | Interview | No | No | No |
| 336 | Wherry AE, McCray CA, Adedeji-Fajobi TI, Sibiya X, Ucko P, Lebina L, Golub JE, Cohen JE, Martinson NA. | A comparative assessment of the price, brands and pack characteristics of illicitly traded cigarettes in five cities and towns in South Africa. | South Africa | 2014 | 40 adults | Adults aged more than 18 years and Cigarettes | Convenience sampling | - | 98.50% | Community/household | Urban | Quantitative | Cross-sectional | Interview | No | No | No |
| 337 | Dutra LM, Williams DR, Gupta J, Kawachi I, Okechukwu CA. | Human rights violations and smoking status among South African adults enrolled in the South Africa Stress and Health (SASH) study. | South Africa | 2014 | 2095 | Adults | Multistage cluster sampling | - | 36% | Community/household | National | Quantitative | Cross-sectional | Interview | No | No | No |
| 338 | Agaku IT, Filippidis FT, Vardavas CI, Odukoya OO, Awopegba AJ, Ayo-Yusuf OA, Connolly GN. | Poly-tobacco use among adults in 44 countries during 2008-2012: evidence for an integrative and comprehensive approach in tobacco control. | Nigeria | 2014 | 9765 | Individuals aged 15 and more | Multistage cluster sampling | - | - | Community/household | National | Quantitative | Cross-sectional | Interview | No | No | No |
| 339 | Ayo-Yusuf OA, Odukoya OO, Olutola BG. | Sociodemographic correlates of exclusive and concurrent use of smokeless and smoked tobacco products among Nigerian men | Nigeria | 2014 | 15453 | Individuals aged 15 to 59 years | Multistage cluster sampling | 31.8 | 100% | Community/household | National | Quantitative | Cross-sectional | Interview | No | No | No |
| 340 | Peer N, Lombard C, Steyn K, Levitt N. | Differential patterns of tobacco use among black men and women in Cape Town: the cardiovascular risk in black South Africans study. | South Africa | 2014 | 1099 | Adults aged 25 - 74 years | Multistage cluster sampling | - | 35.67% | Community/household | Urban | Quantitative | Cross-sectional | Interview | No | Yes | No |
| 341 | Borzekowski DL, Cohen JE. | Young children's perceptions of health warning labels on cigarette packages: a study in six countries. | Nigeria | 2014 | 385 | Children aged 5 or 6 years | Multistage cluster sampling | - | 50.10% | Community/household | Both | Quantitative | Cross-sectional | Interview | No | No | No |
| 342 | Ayo-Yusuf OA, Agaku IT. | Intention to switch to smokeless tobacco use among South African smokers: results from the 2007 South African Social Attitudes Survey. | South Africa | 2014 | 678 | Individuals aged 16 years or more | Multistage stratified probability | - | 48.70% | Community/household | National | Quantitative | Cross-sectional | Interview | No | Yes | No |
| 343 | Dutra LM, Williams DR, Kawachi I, Okechukwu CA. | Racial and non-racial discrimination and smoking status among South African adults 10 years after apartheid. | South Africa | 2014 | 4240 | Adults | Multistage stratified sampling | - | 46% | Community/household | National | Quantitative | Cross-sectional | Interview | No | No | No |
| 344 | Ayo-Yusuf OA, Olutola BG. | Epidemiological association between osteoporosis and combined smoking and use of snuff among South African women | South Africa | 2014 | 2050 | Adults aged 40 or more | Multistage stratified sampling | - | 0% | Community/household | National | Quantitative | Cross-sectional | Interview | No | No | No |
| 345 | Okpataku CI, Kwanashie HO, Ejiofor JI, Olisah VO. | Prevalence and socio-demographic risk factors associated with psychoactive substance use in psychiatric out-patients of a tertiary hospital in Nigeria. | Nigeria | 2014 | 207 | Adult patients | Systematic sampling | - | - | Health facility | Urban | Quantitative | Cross-sectional | Interview | No | No | No |
| 346 | Louwagie GM, Wouters E, Ayo-Yusuf OA. | Poverty and substance use in South African tuberculosis patients. | South Africa | 2014 | 1005 | Adults | Systematic sampling | 40.8 | 100% | Health facility | Urban | Quantitative | Cross-sectional | Interview | No | Yes | No |
| 347 | Green AC, Kaai SC, Fong GT, Driezen P, Quah AC, Burhoo P. | Investigating the effectiveness of pictorial health warnings in Mauritius: findings from the ITC Mauritius survey. | Mauritius | 2014 | 668 | Adults aged 18 years and more | Multistage stratified sampling | 44.2 | 94.30% | Community/household | National | Quantitative | Interventional study | Interview | No | Yes | No |
| 348 | Louwagie GM, Okuyemi KS, Ayo-Yusuf OA. | Efficacy of brief motivational interviewing on smoking cessation at tuberculosis clinics in Tshwane, South Africa: a randomized controlled trial. | South Africa | 2014 | 409 | Adult patients | Purposive sampling | - | 89.98% | Health facility | Urban | Quantitative | Randomized control trial | Interview | No | Yes | No |
| 349 | Griffiths B, Lesosky M, Ntsekhe M. | Self-reported use of evidence-based medicine and smoking cessation 6 - 9 months after acute coronary syndrome: a single-centre perspective. | South Africa | 2014 | 164 | Adults | Purposive sampling | 58.6 | 59.10% | Health facility | Urban | Quantitative | Cross-sectional | Interview and Administrative data/document review | No | No | No |
| 350 | Pefura-Yone EW, Fodjeu G, Kengne AP, Roche N, Kuaban C. | Prevalence and determinants of chronic obstructive pulmonary disease in HIV infected patients in an African country with low level of tobacco smoking. | Cameroon | 2014 | 922 | Adults aged 18 years and more | Systematic sampling | 42.6 | 32.30% | Health facility for cases and Community/household for controls | Urban | Quantitative | Case control | Observation | Yes | Yes | No |
| 351 | Sattar S, Van Schalkwyk C, Claassens M, Dunbar R, Floyd S, Enarson DA, Godfrey-Faussett P, Ayles H, Beyers N. | Symptom reporting among prevalent tuberculosis cases who smoke, are HIV-positive or have hyperglycaemia. | Zambia and South Africa | 2014 | 894 | Adults aged 18 years and more | Multistage cluster sampling | - | 48% | Community/household | Both | Quantitative | Cross-sectional | Observation | No | No | No |
| 352 | Vogelsang M, Paccez JD, Schäfer G, Dzobo K, Zerbini LF, Parker MI. | Aberrant methylation of the MSH3 promoter and distal enhancer in esophageal cancer patients exposed to first-hand tobacco smoke. | South Africa | 2014 | 84 | Adults | Purposive sampling | 60.31 | 50% | Health facility | Urban | Quantitative | Cross-sectional | Observation | No | No | No |
| 353 | Koegelenberg CF, Noor F, Bateman ED, van Zyl-Smit RN, Bruning A, O'Brien JA, Smith C, Abdool-Gaffar MS, Emanuel S, Esterhuizen TM, Irusen EM. | Efficacy of varenicline combined with nicotine replacement therapy vs varenicline alone for smoking cessation: a randomized clinical trial. | South Africa | 2014 | 446 | Adults | Purposive sampling | 46.3 | 38.34% | Health facility | Urban | Quantitative | Randomized control trial | Observation | No | Yes | No |
| 354 | Atari DO. | Gender differences in the prevalence and determinants of tobacco use among school-aged adolescents (11-17 years) in Sudan and South Sudan. | Sudan, South Sudan | 2014 | 4277 | Adolescents | Multistage cluster random sampling | - | 48.30% | Schools | National | Quantitative | Cross-sectional | Self-administered questionnaire | No | Yes | Yes |
| 355 | Agaku IT, Ayo-Yusuf OA. | Awareness of nicotine replacement therapy among South African smokers and their interest in using it for smoking cessation when provided for free | South Africa | 2014 | 689 | Individuals aged 16 years or more | Multistage probability sampling | - | 66.47% | Community/household | National | Quantitative | Cross-sectional | Self-administered questionnaire | No | Yes | No |
| 356 | Agaku IT, Ayo-Yusuf OA, Vardavas CI, Connolly G. | Predictors and patterns of cigarette and smokeless tobacco use among adolescents in 32 countries, 2007-2011. | Botswana, Lesotho, Madagascar, Rwanda, Seychelles, South Africa and Togo | 2014 | - | Adolescents aged 13 to 15 years | Multistage cluster sampling | - | - | Schools | National | Quantitative | Cross-sectional | Self-administered questionnaire | No | Yes | Yes |
| 357 | Agaku IT, Adisa AO, Omaduvie UT, Vardavas CI. | The relationship between proximity of tobacco retail outlets to schools and tobacco use among school personnel in sub-Saharan Africa. | Burkina Faso, Eritrea, Ghana, Guinea Bissau, Lesotho, Malawi, Mauritania, Mauritius, Namibia, Niger, Democratic Republic of Congo, Rwanda, Senegal, Seychelles, Sierra Leone, South Africa, Swaziland, Togo, Uganda | 2014 | 12201 | Adults | Multistage cluster sampling | - | Burkina Faso 70.1, Eritrea 63.6, Ghana 67.5, Guinea Bissau 68.1, Lesotho 33, Malawi 59.9, Mauritania 45.7, Mauritius 44.4, Namibia 44.4, Niger 22.7,Democratic Republic of Congo 75.9, Rwanda 78.9, Senegal 80.4, Seychelles 45.6, Sierra Leone 24.3, South Africa 37.3, Swaziland 41.6, Togo 91.8, Uganda 51.5 | Schools | National | Quantitative | Cross-sectional | Self-administered questionnaire | No | No | No |
| 358 | Reddy PS, James S, Resnicow K, Sewpaul R, Masuka P, van den Borne B. | Prevalence and correlates of smokeless tobacco use among grade 8-11 school students in South Africa: a nationwide study. | South Africa | 2014 | 10270 | Adolescents | Multistage stratified cluster sampling | 16.2 | 49.15 | Schools | National | Quantitative | Cross-sectional | Self-administered questionnaire | No | Yes | No |
| 359 | Peltzer K, Pengpid S. | Tobacco use, beliefs and risk awareness in university students from 24 low, middle and emerging economy countries | - | 2014 | 16953 | Individuals aged 16 to 30 years | Convenience sampling to select universities and Stratified random sampling to select classes | 20.9 | 41% | Schools | Urban | Quantitative | Cross-sectional | Self-administered questionnaire | No | No | No |
| 360 | Agaku, I. T.; Alpert, H. R.; Vardavas, C. I.; Adisa, A. O. & Connolly, G. N. | Use of smokeless tobacco and cigarettes among Nigerian youths: implications for tobacco control policies in Africa | Nigeria | 2014 | 536 | Adolescents | Multistage cluster sampling | 17 | 69% | Schools | Both | Quantitative | Cross-sectional | Self-administered questionnaire | Yes | No | No |
| 361 | Lam E, Giovino GA, Shin M, Lee KA, Rolle I, Asma S. | Relationship between frequency and intensity of cigarette smoking and TTFC/C among students of the GYTS in select countries, 2007-2009. | South Africa | 2014 | 417 | Adolescents | Multistage cluster sampling | - | - | Schools | National | Quantitative | Cross-sectional | Self-administered questionnaire | No | Yes | Yes |
| 362 | Agaku IT, Filippidis FT. | Prevalence, determinants and impact of unawareness about the health consequences of tobacco use among 17,929 school personnel in 29 African countries. | Namibia, Swaziland, South Africa, Lesotho, Burkina Faso, Ghana, Mauritania, Niger, Senegal, Togo, Guinea Bissau, Sierra Leone, Cameroon, Democratic Republic of Congo, Republic of Congo, Central African Republic, Djibouti, Somalia, Malawi, Uganda, Eritea, Seychelles, Mauritius, Rwanda, Zimbabwe, and Sudan | 2014 | 17929 | Adults | Multistage cluster sampling | - | Namibia 44.4%, Swaziland 41.6, South Africa 37.3, Lesotho 33, Burkina Faso 70.1, Ghana 67.5, Mauritania 17, Niger 22.7, Senegal 80.4, Togo 91.8, Guinea Bissau 68.1, Sierra Leone 24.3, Cameroon 58.3, Democratic Republic of Congo 17.8, Republic of Congo 75.9, Central African Republic 78.4, Djibouti 24.6, Somalia 13.3, Malawi 59.9, Uganda 51.5, Eritea 63.6, Seychelles 45.6, Mauritius 44.4, Rwanda 78.9, Zimbabwe 33.9, Morocco 33.5, Tunisia 56.9, Libia 63.3, Sudan 42.7 | Schools | National | Quantitative | Cross-sectional | Self-administered questionnaire | No | No | No |
| 363 | Eticha T, Kidane F. | The prevalence of and factors associated with current smoking among College of Health Sciences students, Mekelle University in northern Ethiopia. | Ethiopia | 2014 | 193 | Young adults aged 18 to 28 years | Multistage stratified sampling | 21.2 | 49.70% | Schools | Urban | Quantitative | Cross-sectional | Self-administered questionnaire | No | Yes | No |
| 364 | Babalola E, Akinhanmi A, Ogunwale A. | Who guards the guards: drug use pattern among medical students in a nigerian university. | Nigeria | 2014 | 246 | Adults aged 22 to 40 years | Convenience sampling | 26.8 | 52.80% | Schools | Urban | Quantitative | Cross-sectional | Self-administered questionnaire | No | No | No |
| 365 | Azodo CC, Omili M. | Tobacco use, Alcohol Consumption and Self-rated Oral Health among Nigerian Prison Officials. | Nigeria | 2014 | 150 | Adults | Systematic sampling | 32.25 | 66.40% | Workplace | Both | Quantitative | Cross-sectional | Self-administered questionnaire | No | Yes | No |
| 366 | Odukoya OO, Odeyemi KA, Oyeyemi AS, Upadhyay RP. | The effect of a short anti-smoking awareness programme on the knowledge, attitude and practice of cigarette smoking among secondary school students in Lagos state, Nigeria. | Nigeria | 2014 | 973 | School going adolescents | Multistage cluster sampling | 14.2 | 47.50% | Schools | Urban | Quantitative | Non-randomized controlled intervention study | Self-administered questionnaire | No | Yes | No |
| 367 | Sitas F, Egger S, Bradshaw D, Groenewald P, Laubscher R, Kielkowski D, Peto R. | Differences among the coloured, white, black, and other South African populations in smoking-attributed mortality at ages 35-74 years: a case-control study of 481,640 deaths. | South Africa | 2014 | 481640 | Reported adult death at the age of 35 - 74 years | Purposive sampling | - | - | - | National | Quantitative | Case control | Administrative data/document review | No | No | No |
| 368 | Sreeramareddy CT, Pradhan PM, Sin S. | Prevalence, distribution, and social determinants of tobacco use in 30 sub-Saharan African countries. | Sub-Saharan Africa | 2014 | - | Inidividuals aged 15 to 49 years | Probability proportional cluster sampling | - | - | Community/household | National | Quantitative | Cross-sectional | Interview | No | No | No |
| 369 | Bosdriesz JR, Mehmedovic S, Witvliet MI, Kunst AE. | Socioeconomic inequalities in smoking in low and mid income countries: positive gradients among women? | Sub-Saharan Africa | 2014 | 233917 | Adults aged 18 years and more | Random sampling | - | - | Community/household | National | Quantitative | Cross-sectional | Interview | No | No | No |
| 370 | Madkour AS, Ledford EC, Andersen L, Johnson CC. | Tobacco advertising/promotions and adolescents' smoking risk in Northern Africa. | Sudan and other African countries | 2014 | 12329 | Adolescents aged 13 - 15 years | Multistage cluster sampling | 14.1 | 51.11% | Schools | National | Quantitative | Cross-sectional | Self-administered questionnaire | No | Yes | Yes |
| 371 | Caleyachetty, Rishi; Tait, Christopher A.; Kengne, Andre P.; Corvalan, Camila; Uauy, Ricardo & Echouff o-Tcheugui, Justin B E | Tobacco use in pregnant women: Analysis of data from Demographic and Health Surveys from 54 low-income and middle-income countries | Benin, Burkina Faso, Burundi, Cameroon, Congo, DR Congo, Cote d'Ivoire, Ethiopia, Gabon, Ghana, Guinea, Kenya, Lesotho, Liberia, Madagascar, Malawi, Mali, Mozambiqu, Namibia, NIger, Nigeria, and Rwanda | 2014 | - | - | Stratified random sampling | - | 0% | Community/household | National | Quantitative | Cross-sectional | Self-administered questionnaire | No | Yes | No |
| 372 | Poluyi EO, Odukoya OO, Aina B, Faseru B. | Tobacco related knowledge and support for smoke-free policies among community pharmacists in Lagos state, Nigeria. | Nigeria | 2015 | 212 | Adults | Multistage cluster sampling | 35.2 | 60.8 | Workplace | Urban | Mixed | Cross-sectional | Self-administered questionnaire and Focus group discussion | No | Yes | No |
| 373 | van Walbeek C, Shai L. | Are the tobacco industry's claims about the size of the illicit cigarette market credible? The case of South Africa. | South Africa | 2015 | - | - | - | - | - | - | National | Qualitative | Case study | Administrative data/document review | No | No | No |
| 374 | Onigbogi OO, Karatu D, Sanusi S, Pratt R, Okuyemi K. | Exploring cigarette use among male migrant workers in Nigeria. | Nigeria | 2015 | 24 | Adult smokers | Convenience sampling | - | - | Workplace | - | Qualitative | Thematic analysis | Focus group discussion | No | Yes | No |
| 375 | Ezeh VC, Mefoh P. | Stimulus Modality and Smoking Behavior: Moderating Role of Implicit Attitudes. | Nigeria | 2015 | 60 | Adults | Purposive sampling | 23.1 | 100% | Schools | Urban | Qualitative | Thematic analysis | Observation | No | Yes | No |
| 376 | Ayo-Yusuf OA, Agaku IT. | The association between smokers' perceived importance of the appearance of cigarettes/cigarette packs and smoking sensory experience: a structural equation model. | South Africa | 2015 | 633 | Individuals aged 16 years and more | Multistage stratified probability sampling | - | 71.9 | Community/household | National | Qualitative | Grounded theory | Self-administered questionnaire | No | Yes | No |
| 377 | Kidane A, Mduma J, Naho A, Hu TW. | Impact of Smoking on Nutrition and the Food Poverty Level in Tanzania. | Tanzania | 2015 | 9422 | Adults | - | - | - | Community/household | National | Quantitative | Cross-sectional | - | No | No | No |
| 378 | Kidane A, Mduma J, Naho A, Ngeh ET, Hu TW. | The Demand for Cigarettes in Tanzania and Implications for Tobacco Taxation Policy. | Tanzania | 2015 | 10486 | Adults | - | - | 74.05% | Community/household | National | Quantitative | Cross-sectional | Interview | No | No | No |
| 379 | Kanyoni M, Gishoma D, Ndahindwa V. | Prevalence of psychoactive substance use among youth in Rwanda. | Rwanda | 2015 | 2479 | Adolescents and adults aged 14 to 35 | Multistage cluster sampling | 23.2 | 56% | Community/household | National | Quantitative | Cross-sectional | Interview | No | No | No |
| 380 | Salloum RG, Goma F, Chelwa G, Cheng X, Zulu R, Kaai SC, Quah AC, Thrasher JF, Fong GT. | Cigarette price and other factors associated with brand choice and brand loyalty in Zambia: findings from the ITC Zambia Survey. | Zambia | 2015 | 846 | Adults | Multistage cluster sampling | - | - | Community/household | National | Quantitative | Cross-sectional | Interview | No | Yes | No |
| 381 | Achia TN. | Tobacco use and mass media utilization in sub-Saharan Africa. | Sub-Saharan Africa | 2015 | 159462 | Adults (Women aged 15-49 years and men aged 15-59 years) | Multistage cluster sampling | - | 36.46% | Community/household | National | Quantitative | Cross-sectional | Interview | No | No | No |
| 382 | Uguru NP, Mbachu C, Ibe OP, Uguru CC, Odukoya O, Okwuosa C, Onwujekwe O. | Investigating male tobacco use and expenditure patterns across socio-economic groups in Nigeria. | Nigeria | 2015 | 15486 | Individuals aged 15 to 59 years | Multistage cluster sampling | - | 100% | Community/household | National | Quantitative | Cross-sectional | Interview | No | Yes | No |
| 383 | Reddy P, Zuma K, Shisana O, Kim J, Sewpaul R. | Prevalence of tobacco use among adults in South Africa: Results from the first South African National Health and Nutrition Examination Survey. | South Africa | 2015 | 15401 | Adults aged 18 years and more | Multistage cluster sampling | - | 47.80% | Community/household | National | Quantitative | Cross-sectional | Interview | No | No | No |
| 384 | Lakew Y, Haile D. | Tobacco use and associated factors among adults in Ethiopia: further analysis of the 2011 Ethiopian Demographic and Health Survey. | Ethiopia | 2015 | 30625 | Adolescents and adults aged 15 to 59 years | Multistage stratified cluster sampling | 29 | 46.10% | Community/household | National | Quantitative | Cross-sectional | Interview | No | No | No |
| 385 | Kaleta D, Polanska K, Usidame B. | Smoke-Free Workplaces Are Associated with Protection from Second-Hand Smoke at Homes in Nigeria: Evidence for Population-Level Decisions. | Nigeria | 2015 | 9765 | Individuals aged 15 years and older | Multistage cluster sampling | - | 60.60% | Community/household | National | Quantitative | Cross-sectional | Interview | No | No | No |
| 386 | Kidane A, Hepelwa A, Ngeh ET, Hu TW. | Healthcare Cost of Smoking Induced Cardiovascular Disease in Tanzania. | Tanzania | 2015 | 123 | Adults | - | - | 51.60% | Health facility | Urban | Quantitative | Cross-sectional | Interview | No | No | No |
| 387 | Aguocha CM, Aguocha JK, Igwe M, Uwakwe RU, Onyeama GM. | Prevalence and correlates of cigarette smoking among patients with schizophrenia in southeast Nigeria. | Nigeria | 2015 | 367 | Adults | Simple random sampling | 34.1 | 48.50% | Health facility | Urban | Quantitative | Cross-sectional | Interview | No | Yes | No |
| 388 | Chivese T, Esterhuizen TM, Basson AR. | The Influence of Second-Hand Cigarette Smoke Exposure during Childhood and Active Cigarette Smoking on Crohn's Disease Phenotype Defined by the Montreal Classification Scheme in a Western Cape Population, South Africa. | South Africa | 2015 | 194 | Adults aged 18 to 70 years | Systematic sampling | 47 | 27.30% | Health facility | Urban | Quantitative | Cross-sectional | Interview | No | Yes | No |
| 389 | Obiora CC, Dim CC, Uzochukwu BS, Ezugwu FO. | Cigarette smoking and perception of its advertisement among antenatal clinic attendees in referral health facilities in Enugu, Nigeria. | Nigeria | 2015 | 200 | Adults | Systematic random sampling | 29.3 | 0% | Health facility | Urban | Quantitative | Cross-sectional | Interview | No | Yes | No |
| 390 | Oye-Adeniran BA, Aina OF, Gbadegesin A, Ekanem EE. | Substance use and sexual behaviour among female students in Nigerian universities. | Nigeria | 2015 | 2408 | Adults students aged 18 or more | Multistage stratified sampling | 21.6 | 0% | Schools | Urban | Quantitative | Cross-sectional | Interview | No | Yes | No |
| 391 | Onigbogi OO, Odukoya O, Onigbogi M, Sekoni O. | Knowledge and attitude toward smoke-free legislation and second-hand smoking exposure among workers in indoor bars, beer parlors and discotheques in Osun State of Nigeria. | Nigeria | 2015 | 154 | Individuals aged 16 to 80 years | Convenience sampling | 28.1 | 48.00% | Workplace | Urban | Quantitative | Cross-sectional | Interview | No | Yes | No |
| 392 | Sorsdahl K, Stein DJ, Corrigall J, Cuijpers P, Smits N, Naledi T, Myers B. | The efficacy of a blended motivational interviewing and problem solving therapy intervention to reduce substance use among patients presenting for emergency services in South Africa: A randomized controlled trial. | South Africa | 2015 | 335 | Adults | Purposive sampling | 28 | 65.50% | Health facility | Urban | Quantitative | Randomized control trial | Interview | Yes | Yes | No |
| 393 | Ande A, McArthur C, Ayuk L, Awasom C, Achu PN, Njinda A, Sinha N, Rao PS, Agudelo M, Nookala AR, Simon S, Kumar A, Kumar S. | Effect of mild-to-moderate smoking on viral load, cytokines, oxidative stress, and cytochrome P450 enzymes in HIV-infected individuals | Cameroon | 2015 | 32 | Adults aged 21 to 65 years | Purposive sampling | - | 56.25% | Health facility | Both | Quantitative | Cohort | Observation | Yes | Yes | No |
| 394 | Gray D, Czövek D, Smith E, Willemse L, Alberts A, Gingl Z, Hall GL, Zar HJ, Sly PD, Hantos Z. | Respiratory impedance in healthy unsedated South African infants: effects of maternal smoking. | South Africa | 2015 | 164 | Infants | - | - | 50% | Health facility | Both | Quantitative | Cross-sectional | Observation | No | No | No |
| 395 | Louwagie GM, Ayo-Yusuf OA. | Predictors of tobacco smoking abstinence among tuberculosis patients in South Africa. | South Africa | 2015 | 409 | Adults aged 18 years and more | Random sampling | 41.3 | 90% | Health facility | Urban | Quantitative | Randomized control trial | Observation | No | Yes | No |
| 396 | Asiki G, Baisley K, Kamali A, Kaleebu P, Seeley J, Newton R. | A prospective study of trends in consumption of cigarettes and alcohol among adults in a rural Ugandan population cohort, 1994-2011. | Uganda | 2015 | 2903 in 1994/95, 6673 in 2008/09, 7809 in 2010/11 | Adolescents and adults aged 13 and more | Convenience sampling | - | 48.1 in 1998/99, 40.7 in 2008/09, and 43.7 in 2010/11 | Community/household | Rural | Quantitative | Cohort | Interview | No | No | No |
| 397 | Kidane A, Mduma J, Naho A, Hu TW. | Impact of Smoking on Food Expenditure among Tanzanian Households. | Tanzania | 2015 | 10464 | Households | - | - | - | Community/household | National | Quantitative | Cross-sectional | Self-administered questionnaire | No | No | No |
| 398 | Perl R, Murukutla N, Occleston J, Bayly M, Lien M, Wakefield M, Mullin S. | Responses to antismoking radio and television advertisements among adult smokers and non-smokers across Africa: message-testing results from Senegal, Nigeria and Kenya. | Senegal, Nigeria, and Kenya | 2015 | 1078 | Adults | Convenience sampling | - | - | Community/household | Urban | Quantitative | Cross-sectional | Self-administered questionnaire | No | No | No |
| 399 | Ayo-Yusuf OA, Olutola BG, Agaku IT. | Cigarette Smoking Trends and Social Disparities Among South African Adults, 2003-2011. | South Africa | 2015 | 6927 | Adults | Multistage stratified sampling | - | 45.90% | Community/household | National | Quantitative | Cross-sectional | Self-administered questionnaire | No | No | No |
| 400 | Shirinde J, Wichmann J, Voyi K. | Environmental tobacco smoke and the risk of eczema symptoms among school children in South Africa: a cross-sectional study. | South Africa | 2015 | 3424 | Adolescents | Multistage cluster sampling | - | 47.70% | Schools | Urban | Quantitative | Cross-sectional | Self-administered questionnaire | No | Yes | No |
| 401 | Dania MG, Ozoh OB, Bandele EO. | Smoking habits, awareness of risks, and attitude towards tobacco control policies among medical students in Lagos, Nigeria. | Nigeria | 2015 | 250 | School going adults aged 16 to 33 years | Multistage stratified sampling | 21.4 | 53.20% | Schools | Urban | Quantitative | Cross-sectional | Self-administered questionnaire | Yes | No | No |
| 402 | Veeranki SP, Alzyoud S, Kheirallah KA, Pbert L. | Waterpipe Use and Susceptibility to Cigarette Smoking Among Never-Smoking Youth. | Comoros, Djibouti, Mauritania, Morocco, Somalia, and Sudan | 2015 | 39710 (Comoros: 811, Djibouti: 1096, Egypt: 3472, Libya: 1361, Mauritania: 1769, Morocco: 2106, Somalia: 897, Sudan: 950, and Tunisia: 1294) | Adolescents aged 13 to 15 years | - | - | - | Schools | National | Quantitative | Cross-sectional | Self-administered questionnaire | No | No | Yes |
| 403 | Agaku IT, Maliselo T, Ayo-Yusuf OA. | The relationship between secondhand smoke exposure, pro-tobacco social influences, and smoking susceptibility among nonsmoking Zambian adolescents. | Zambia | 2015 | 3377 | Adolescents | Multistage cluster sampling | - | 49.20% | Schools | National | Quantitative | Cross-sectional | Self-administered questionnaire | No | Yes | Yes |
| 404 | Mamudu HM, Veeranki SP, John RM, Kioko DM, Ogwell Ouma AE. | Secondhand Smoke Exposure Among Nonsmoking Adolescents in West Africa | Cape Verde, Cote d'Ivoire, Ghana, Guinea, Mali, Mauritania, Niger, Senegal, and Togo | 2015 | 12892 | Adolescents aged 13 to 15 years | Multistage cluster sampling | - | - | Schools | National | Quantitative | Cross-sectional | Self-administered questionnaire | No | Yes | Yes |
| 405 | Veeranki SP, Mamudu HM, Zheng S, John RM, Cao Y, Kioko D, Anderson J, Ouma AE | Secondhand smoke exposure among never-smoking youth in 168 countries | Benin, Botswana, Burkina Faso, Burundi, Cameroon, Cape Verde, Central African Republic- Bangui, Chad, Comoros, Congo, Côte d’Ivoire, Democratic Republic of Congo, Equatorial Guinea, Eritrea, Ethiopia, Gambia, Ghana, Guinea, Guinea- Bissau, Kenya, Lesotho, Liberia, Madagascar, Malawi, Mali, Mauritania, Mauritius, Mozambique, Namibia, Niger, Nigeria, Rwanda, Senegal, Seychelles, Sierra Leone, South Africa, Swaziland, Tanzania, Togo, Uganda, Zambia, Zimbabwe. | 2015 | 4E+05 | Adolescents aged 13 to 15 years | Multistage cluster sampling | - | 49.70% | Schools | National | Quantitative | Cross-sectional | Self-administered questionnaire | No | Yes | Yes |
| 406 | Veeranki SP, Mamudu HM, John RM, Ouma AE. | Prevalence and correlates of tobacco use among school-going adolescents in Madagascar | Madagascar | 2015 | 1184 | Adolescents aged 13 to 15 years | Multistage cluster sampling | - | 45.20% | Schools | National | Quantitative | Cross-sectional | Self-administered questionnaire | No | Yes | Yes |
| 407 | Odukoya OO, Chife JO, Odeyemi KA, Nwangwu GI. | Young peoples awareness and support for tobacco control legislation: A study among in-school youth in Lagos, Nigeria. | Nigeria | 2015 | 950 | School going adolescents | Multistage cluster sampling | 14.1 | 51.60% | Schools | Urban | Quantitative | Cross-sectional | Self-administered questionnaire | No | No | No |
| 408 | Uti O, Sofola O. | Impact of an educational intervention on smoking counseling practice among Nigerian dentists and dental students | Nigeria | 2015 | 136 | Adults | Convenience sampling | - | 52.20% | Workplace | Urban | Quantitative | Quasi-experimental | Self-administered questionnaire | No | No | No |
| 409 | Zhao L, Palipudi KM, Ramanandraibe N, Asma S. | Cigarette smoking and cigarette marketing exposure among students in selected African countries: Findings from the Global Youth Tobacco Survey. | Congo, Cote D'Ivore, Ghana, Mauritania, Malawi, Niger, South Africa, Swaziland, Uganda, and Zambia | 2015 | Congo: 1106, Cote D'Ivore: 1904, Ghana: 4098, Mauritania: 1755, Malawi:1284, Niger: 1305, South Africa: 3917, Swaziland: 1239, Uganda: 2020, and Zambia: 1935 | School going adolescents aged 13 to 15 years | Multistage cluster sampling | - | - | Schools | National | Quantitative | Cross-sectional | Self-administered questionnaire | No | No | Yes |
| 410 | Winkler V, Mangolo NJ, Becher H. | Lung cancer in South Africa: a forecast to 2025 based on smoking prevalence data. | South Africa | 2015 | 8090 | Adults | Multistage cluster sampling | - | - | Community/household | National | Quantitative | Cross-sectional | Interview | No | No | No |
| 411 | Lown EA, McDaniel PA, Malone RE. | Tobacco is "our industry and we must support it": Exploring the potential implications of Zimbabwe's accession to the Framework Convention on Tobacco Control. | Zimbabwe | 2016 | - | - | - | - | - | - | - | Qualitative | Case study | Administrative data/document review | No | No | No |
| 412 | Lencucha R, Drope J, Labonte R, Zulu R, Goma F. | Investment incentives and the implementation of the Framework Convention on Tobacco Control: evidence from Zambia. | Zambia | 2016 | 23 | Adult government employees | Purposive sampling | - | - | Workplace | National | Qualitative | Case study | Interview | No | No | No |
| 413 | Vellios N, van Walbeek C. | Determinants of regular smoking onset in South Africa using duration analysis. | South Africa | 2016 | 17691 | Individuals aged 15 to 48 years | Multistage cluster sampling | - | 43.90% | Community/household | National | Quantitative | Cross-sectional | - | No | No | No |
| 414 | Bronner Murrison L, Martinson N, Moloney RM, Msandiwa R, Mashabela M, Samet JM, Golub JE. | Tobacco Smoking and Tuberculosis among Men Living with HIV in Johannesburg, South Africa: A Case-Control Study. | South Africa | 2016 | 279 | Adults aged 25 years and more | Convenience sampling | 38 | 100% | Health facility | Urban | Quantitative | Case control | Interview | No | Yes | No |
| 415 | Sewram V, Sitas F, O'Connell D, Myers J. | Tobacco and alcohol as risk factors for oesophageal cancer in a high incidence area in South Africa. | South Africa | 2016 | 1858 | Adults | Purposive sampling | - | 51.39% | Health facility | Urban | Quantitative | Case control | Interview | No | Yes | No |
| 416 | Abiola A, Balogun O, Odukoya O, Olatona F, Odugbemi T, Moronkola R, Solanke A, Akintunde O, Fatoba O. | Age of initiation, Determinants and Prevalence of Cigarette Smoking among Teenagers in Mushin Local Government Area of Lagos State, Nigeria. | Nigeria | 2016 | 402 | Adolescents | Multistage cluster sampling | 16.4 | 63.40% | Community/household | Urban | Quantitative | Cross-sectional | Interview | No | No | No |
| 417 | Morojele NK, Brook JS, Brook DW. | Tobacco and alcohol use among adolescents in South Africa: shared and unshared risks. | South Africa | 2016 | 736 | Adolescents aged 12 to 17 years | Multistage cluster sampling | 14.6 | - | Community/household | Urban | Quantitative | Cross-sectional | Interview | No | Yes | No |
| 418 | Tee GH, Aris T, Rarick J, Irimie S. | Social Determinants of Health and Tobacco Use in Five Low and Middle-Income Countries - Results from the Global Adult Tobacco Survey (GATS), 2011 - 2012. | Nigeria | 2016 | 9765 | Individuals aged 15 years or more | Multistage cluster sampling | - | 50.00% | Community/household | National | Quantitative | Cross-sectional | Interview | No | No | No |
| 419 | Uwakwe R, Gureje O. | Sociodemographic correlates of continuing tobacco use - a descriptive report from the Nigerian Survey of Mental Health and Wellbeing. | Nigeria | 2016 | 1137 | Adults aged 18 years and more | Multistage cluster sampling | - | 95% | Community/household | Both | Quantitative | Cross-sectional | Interview | No | Yes | No |
| 420 | Akinyemi JO, Adedini SA, Wandera SO, Odimegwu CO. | Independent and combined effects of maternal smoking and solid fuel on infant and child mortality in sub-Saharan Africa. | Sub-saharan Africa (Burkina Faso, Burundi, Camoros, Cote D'Ivore, Gabon, Lesotho, Madagascar, Mozambique, Namibia, Niger, Rwanda, Sierra Leone, and Zambia) | 2016 | 143602 | Children aged less than 5 years | Multistage cluster sampling | - | - | Community/household | National | Quantitative | Cross-sectional | Interview | No | No | No |
| 421 | Kabwama SN, Ndyanabangi S, Mutungi G, Wesonga R, Bahendeka SK, Guwatudde D. | Tobacco use and associated factors among Adults in Uganda: Findings from a nationwide survey. | Uganda | 2016 | 3983 | Adults aged 18 and more | Multistage stratified sampling | - | 40.20% | Community/household | National | Quantitative | Cross-sectional | Interview | No | No | No |
| 422 | Kane JC, Murray LK, Bass JK, Johnson RM, Bolton P. | Validation of a substance and alcohol use assessment instrument among orphans and vulnerable children in Zambia using Audio Computer Assisted Self-Interviewing (ACASI). | Zambia | 2016 | 502 | Adolescents aged 13 to 17 years | Purposive sampling | 15 | 45.80% | Community/household | Urban | Quantitative | Cross-sectional | Interview | No | No | No |
| 423 | Odukoya OO, Dada MR, Olubodun T, Igwilo UA, Ayo-Yusuf OA. | Risk Perception and Correlates of Tobacco Use among Young People Outside of Formal School Settings in Lagos State, Nigeria. | Nigeria | 2016 | 326 | Young adults aged 15 to 24 years | Random sampling | 20.96 | 84.70% | Community/household | Both | Quantitative | Cross-sectional | Interview | No | Yes | No |
| 424 | Kirenga BJ, Jones R, Muhofa A, Nyakoojo G, Williams S. | Rapid assessment of the demand and supply of tobacco dependence pharmacotherapy in Uganda. | Uganda | 2016 | 56 | Adults | Purposive sampling | 57.5 | 71.00% | Health facility | Urban | Quantitative | Cross-sectional | Interview | No | Yes | No |
| 425 | Mwiru RS, Nagu TJ, Kaduri P, Mugusi F, Fawzi W. | Prevalence and patterns of cigarette smoking among patients co-infected with human immunodeficiency virus and tuberculosis in Tanzania. | Tanzania | 2016 | 518 | Individuals aged more than 15 years | Purposive sampling | 37 | 54.10% | Health facility | Urban | Quantitative | Cross-sectional | Interview | No | Yes | No |
| 426 | Davis GP, Tomita A, Baumgartner JN, Mtshemla S, Nene S, King H, Susser E, Burns JK. | Substance use and duration of untreated psychosis in KwaZulu-Natal, South Africa. | South Africa | 2016 | 87 | Adult psychiatric patients aged more than 21 years | Systematic random sampling | - | 56.30% | Health facility | Urban | Quantitative | Cross-sectional | Interview | No | Yes | No |
| 427 | Lasebikan VO, Ola BA. | Community-Based Screening, Brief Intervention, and Referral for Treatment for Unhealthy Tobacco Use: Single Arm Study Experience and Implementation Success in Rural and Semi-Rural Settings, South-West Nigeria. | Nigeria | 2016 | 1203 | Individuals aged 15 years or more | Multistage stratified sampling | 24.45 | 51.80% | Community/household | Rural | Quantitative | Non-randomized clinical trial | Interview | No | Yes | No |
| 428 | Du Plooy JL, Macharia M, Verster C. | Cigarette smoking, nicotine dependence, and motivation to quit smoking in South African male psychiatric inpatients. | South Africa | 2016 | 116 | Adults aged more than 18 years | Systematic random sampling | 30.3 | 100% | Health facility | Urban | Quantitative | Cross-sectional | Interview and Administrative data/document review | No | Yes | No |
| 429 | Okello S, Churchill C, Owori R, Nasasira B, Tumuhimbise C, Abonga CL, Mutiibwa D, Christiani DC, Corey KE. | Population attributable fraction of Esophageal squamous cell carcinoma due to smoking and alcohol in Uganda. | Uganda | 2016 | 209 | Adult patients aged 30 or more | Purposive sampling | 51 | 59% | Health facility | Urban | Quantitative | Case control | Observation | No | No | No |
| 430 | Elkhader BA, Abdulla AA, Ali Omer MA. | Correlation of Smoking and Myocardial Infarction Among Sudanese Male Patients Above 40 Years of Age. | Sudan | 2016 | 144 | Adult patients more than 40 years | Purposive sampling | - | 100.00% | Health facility | Urban | Quantitative | Cohort | Observation | No | Yes | No |
| 431 | Song MA, Marian C, Brasky TM, Reisinger S, Djordjevic M, Shields PG. | Chemical and toxicological characteristics of conventional and low-TSNA moist snuff tobacco products. | South Africa | 2016 | - | Snuff products | Purposive sampling | - | - | - | - | Quantitative | Cross-sectional | Observation | No | No | No |
| 432 | Uthman OA, Ekström AM, Moradi TT. | Influence of socioeconomic position and gender on current cigarette smoking among people living with HIV in sub-Saharan Africa: disentangling context from composition. | Sub-saharan Africa (Burkina Faso, Burundi, Cameroon, Cote D'Ivore, Ethiopia, Gabon, Ghana, Kenya, Lesotho, Liberia, Malawi, Niger, Rwanda, Sao Tome and Principe, Senegal, Sierra Leone, Swaziland, Zambia, and Zimbabwe) | 2016 | 31270 | Adults | Multistage stratified sampling | - | 42.40% | Community/household | National | Quantitative | Cross-sectional | Observation | No | No | No |
| 433 | Govind N, Ally MM, Tikly M, Anderson R, Hodkinson B, Meyer PW. | Pitfalls in the assessment of smoking status detected in a cohort of South African RA patients. | South Africa | 2016 | 218 | Adults | - | - | - | Health facility | - | Quantitative | Cross-sectional | Observation | No | No | No |
| 434 | Nemakhavhani TR, Akinsola HA. | Survey of bar-lounges and restaurants regarding compliance with the current smoke-free regulation in Thulamela Municipality, South Africa. | South Africa | 2016 | 56 | Bar-lounges and restaurants | Convenience sampling | - | - | Recreational facility | Rural | Quantitative | Cross-sectional | Observation | No | Yes | No |
| 435 | van Gemert F, Chavannes N, Kirenga B, Jones R, Williams S, Tsiligianni I, Vonk J, Kocks J, de Jong C, van der Molen T. | Socio-economic factors, gender and smoking as determinants of COPD in a low-income country of sub-Saharan Africa: FRESH AIR Uganda. | Uganda | 2016 | 588 | Adults aged more than 30 years | - | 44.9 | 49.90% | Community/household | Rural | Quantitative | Cross-sectional | Observation | No | No | No |
| 436 | Mbulo L, Ogbonna N, Olarewaju I, Musa E, Salandy S, Ramanandraibe N, Palipudi K; GATS collaborative group.. | Preventing tobacco epidemic in LMICs with low tobacco use - Using Nigeria GATS to review WHO MPOWER tobacco indicators and prevention strategies. | Nigeria | 2016 | 9765 | Individuals aged 15 years or more | Multistage cluster sampling | - | 50% | Community/household | National | Quantitative | Cross-sectional | Self-administered questionnaire | No | No | No |
| 437 | English LM, Hsia J, Malarcher A. | Tobacco advertising, promotion, and sponsorship (TAPS) exposure, anti-TAPS policies, and students' smoking behavior in Botswana and South Africa. | Botswana and South Africa | 2016 | 8733 | Adolescents aged 13-15 years old | Multistage cluster sampling | - | - | Schools | National | Quantitative | Cross-sectional | Self-administered questionnaire | No | Yes | Yes |
| 438 | Adebiyi AO, Uchendu OC, Bamgboye E, Ibitoye O, Omotola B. | Perceived effectiveness of graphic health warnings as a deterrent for smoking initiation among adolescents in selected schools in southwest Nigeria. | Nigeria | 2016 | 544 | Adolescents aged 13 to 17 years | Multistage cluster sampling | - | 44.70% | Schools | Rural | Quantitative | Cross-sectional | Self-administered questionnaire | No | No | No |
| 439 | Okagua J, Opara P, Alex-Hart BA. | Prevalence and determinants of cigarette smoking among adolescents in secondary schools in Port Harcourt, Southern Nigeria. | Nigeria | 2016 | 1120 | Adolescents aged 10 to 19 years | Multistage stratified sampling | 16.13 | 55.30% | Schools | Urban | Quantitative | Cross-sectional | Self-administered questionnaire | No | No | No |
| 440 | Osman T, Victor C, Abdulmoneim A, Mohammed H, Abdalla F, Ahmed A, Ali E, Mohammed W. | Epidemiology of Substance Use among University Students in Sudan. | Sudan | 2016 | 410 | Young adults | Proportional quota sampling | 19.6 | 29.80% | Schools | Urban | Quantitative | Cross-sectional | Self-administered questionnaire | No | No | No |
| 441 | Abbo C, Okello ES, Muhwezi W, Akello G, Ovuga E. | Alcohol, Substance Use and Psychosocial Competence of Adolescents in Selected Secondary Schools in Uganda: A Cross Sectional Survey. | Uganda | 2016 | 2902 | Adolescents and youths aged 12 to 24 years | Proportionate multistage sampling | 16.5 | 50% | Schools | Both | Quantitative | Cross-sectional | Self-administered questionnaire | No | No | No |
| 442 | Kruger L, van Walbeek C, Vellios N. | Waterpipe and Cigarette Smoking among University Students in the Western Cape, South Africa. | South Africa | 2016 | 4578 | Adults | Purposive sampling | - | 45.20% | Schools | Both | Quantitative | Cross-sectional | Self-administered questionnaire | No | No | No |
| 443 | Motamedi M, Caldwell L, Wegner L, Smith E, Jones D. | Girls Just Want to Know Where to Have Fun: Preventing Substance Use Initiation in an Under-Resourced Community in South Africa Through HealthWise. | South Africa | 2016 | 5610 | School going adolescents | Random sampling | 14 | - | Schools | Urban | Quantitative | Randomized control trial | Self-administered questionnaire | No | No | No |
| 444 | Lee KA, Palipudi KM, English LM, Ramanandraibe N, Asma S; GYTS collaborative group.. | Secondhand smoke exposure and susceptibility to initiating cigarette smoking among never-smoking students in selected African countries: Findings from the Global Youth Tobacco Survey. | Botswana, Burundi, Cape Verde, Chad, Comoros, Congo, Cote d'Ivoire, Equatorial Guinea, Eritrea, Ghana, Guinea, Kenya, Lesotho, Madagascar, Malawi, Mali, Mauritania, Mauritius, Namibia, Niger, Rwanda, Sao Tome and Principe, Senegal, Seychelles, South Africa, Swaziland, Togo, Uganda, and Zambia | 2016 | Botswana: 1611, Burundi: 1110, Cape Verde: 1188, Chad: 905, Comoros: 811, Congo: 1141, Cote d'Ivoire: 1920, Equatorial Guinea: 1345, Eritrea: 4813, Ghana: 4171, Guinea: 1305, Kenya: 6768, Lesotho: 1604, Madagascar: 1184, Malawi: 1293, Mali: 2135, Mauritania: 1769, Mauritius: 1286, Namibia: 1397, Niger: 1307, Rwanda: 688, Sao Tome and Principe: 3638, Senegal: 1611, Seychelles: 844, South Africa: 3947, Swaziland: 1239, Togo: 1947, Uganda: 2026, and Zambia: 1964 | School going adolescents aged 13 to 15 years | Multistage cluster sampling | - | - | Schools | National | Quantitative | Cross-sectional | Self-administered questionnaire | No | No | Yes |
| 445 | Chandora R, Song Y, Chaussard M, Palipudi KM, Lee KA, Ramanandraibe N, Asma S; GYTS collaborative group.. | Youth access to cigarettes in six sub-Saharan African countries. | Cote d'Ivore, Ghana, Republic of Congo, South Africa, Swaziland, and Uganda | 2016 | Cote d'Ivore: 1920, Ghana: 4171, Republic of Congo: 1141, South Africa: 3947, Swaziland: 1239, and Uganda: 2026 | School going adolescents aged 13 to 15 years | Multistage cluster sampling | - | - | Schools | National | Quantitative | Cross-sectional | Self-administered questionnaire | No | No | Yes |
| 446 | Egbe CO, Bialous SA, Glantz SA. | Avoiding "a massive spin-off effect in West Africa and beyond": The tobacco industry stymies tobacco control in Nigeria. | Nigeria | 2017 | - | - | - | - | - | - | - | Qualitative | Case study | Administrative data/document review | No | No | No |
| 447 | Odukoya O, Jamda M, Onigbogi O, Uguru N, Onigbogi M, James F, Faseru B, Leischow S, Ayo-Yusuf O. | Tobacco Cessation Interventions in Tertiary Hospitals in Nigeria: An Audit of Patient Records. | Nigeria | 2017 | 1588 | Adolescents and adults aged 12 or more | Systematic random sampling | 43 | 45% | Health facility | Both | Quantitative | Cross-sectional | Administrative data/document review | No | Yes | No |
| GYTS – Global Youth Tobacco Survey | | | | | | | | | | | | | | | | | |
